# Delivering precision oncology in metastatic breast cancer: Clinical impact of comprehensive genomic profiling—The CATCH experience

**DOI:** 10.1002/ijc.70208

**Published:** 2025-10-31

**Authors:** Mario Hlevnjak, Sabine Heublein, Verena Thewes, Lukas Wagener, Constantin Pixberg, Carlo Fremd, Laura Michel, Christian Maurer, Lars Buschhorn, Nicola Dikow, Fangyoumin Feng, Stefan Fröhling, Christel Herold‐Mende, Steffen Hirsch, Chen Hong, Daniel Hübschmann, Lena Jassowicz, Polina Kozyulina, Katrin Pfütze, Richard F. Schlenk, Hans‐Peter Sinn, Katharina Smetanay, Christoph Springfeld, Albrecht Stenzinger, Celina Wagner, Stephan Wolf, Andreas Trumpp, Dirk Jäger, Oliver Zivanovic, Marc Zapatka, Andreas Schneeweiss, Peter Lichter

**Affiliations:** ^1^ Molecular Precision Oncology Program, National Center for Tumor Diseases (NCT), NCT Heidelberg, A Partnership Between DKFZ, The University Hospital Heidelberg (UKHD), The Heidelberg Medical Faculty of the Heidelberg University, and The Thorax Clinic Heidelberg Heidelberg Germany; ^2^ Department of Gynecology and Obstetrics University Hospital Heidelberg Heidelberg Germany; ^3^ National Center for Tumor Diseases (NCT), NCT Heidelberg, A Partnership Between DKFZ, The University Hospital Heidelberg (UKHD), The Heidelberg Medical Faculty of the Heidelberg University, and The Thorax Clinic Heidelberg Heidelberg Germany; ^4^ Department of Gynecology and Obstetrics University Hospital Ulm Ulm Germany; ^5^ Division of Molecular Genetics, German Cancer Research Center (DKFZ) Heidelberg Germany; ^6^ Department of Medical Oncology National Center for Tumor Diseases (NCT), NCT Heidelberg, A Partnership Between DKFZ, The University Hospital Heidelberg (UKHD), The Heidelberg Medical Faculty of the Heidelberg University, and The Thorax Clinic Heidelberg Heidelberg Germany; ^7^ Department I of Internal Medicine Center for Integrated Oncology Aachen Bonn Cologne Düsseldorf, Faculty of Medicine and University Hospital Cologne, University of Cologne Cologne Germany; ^8^ Institute of Human Genetics, Heidelberg University Heidelberg Germany; ^9^ Division of Translational Medical Oncology, German Cancer Research Center (DKFZ) Heidelberg Germany; ^10^ German Cancer Consortium (DKTK) Heidelberg Germany; ^11^ Division of Experimental Neurosurgery, Department of Neurosurgery Medical Faculty Heidelberg, University of Heidelberg Heidelberg Germany; ^12^ Hopp Children's Cancer Center Heidelberg (KiTZ), Division of Pediatric Neurooncology DKFZ; German Cancer Consortium (DKTK), Partner Site Heidelberg Heidelberg Germany; ^13^ Computational Oncology Group (CO), Molecular Precision Oncology Program (MPOP), German Cancer Research Center (DKFZ) Heidelberg Germany; ^14^ Sample Processing Laboratory, German Cancer Research Center (DKFZ) and National Center for Tumor Diseases (NCT), NCT Heidelberg, A Partnership Between DKFZ, The University Hospital Heidelberg (UKHD), The Heidelberg Medical Faculty of the Heidelberg University, and The Thorax Clinic Heidelberg Heidelberg Germany; ^15^ NCT Clinical Trial Center, National Center for Tumor Diseases (NCT), NCT Heidelberg a Partnership Between DKFZ, The University Hospital Heidelberg (UKHD), The Heidelberg Medical Faculty of the HeidelbergUniversity, and The Thorax Clinic Heidelberg Heidelberg Germany; ^16^ Department of Internal Medicine V University Hospital Heidelberg Germany; ^17^ Institute for Pathology, University Hospital Heidelberg Heidelberg Germany; ^18^ Department of Obstetrics and Gynecology University Hospital, LMU Munich Germany; ^19^ Next Generation Sequencing Core Facility, German Cancer Research Center DKFZ Heidelberg Germany; ^20^ Division of Stem Cells and Cancer German Cancer Research Center (DKFZ) and DKFZ‐ZMBH Alliance Heidelberg Germany; ^21^ Heidelberg Institute for Stem Cell Technology and Experimental Medicine (HI‐STEM gGmbH) Heidelberg Germany

**Keywords:** breast cancer, precision oncology, real‐world study, whole genome sequencing, whole transcriptome sequencing

## Abstract

CATCH is a prospective precision oncology registry trial that exploits whole‐genome/exome‐ and RNA‐sequencing to enable actionable biomarker detection in metastatic breast cancer (mBC) patients of any subtype. We herein report long‐term follow‐up of the first 558 patients consecutively recruited into CATCH in a monocentric setting between June 2017 and October 2021. Main outcome measures were the rate of implementation of molecular tumor board (MTB) recommended treatments and treatment response as assessed by disease control rate, objective response rate and PFS ratio. Out of the recruited patients, 412 (54.9% HR+/HER2−, 31.3% TNBC, 6.8% HR−/HER2+ and 7.0% HR+/HER2+) were reviewed in the MTB. An appropriate molecularly guided anti‐cancer treatment as recommended by MTB was implemented in 183 (44.4%) patients. Gene expression and computationally derived composite biomarkers further expanded treatment options in up to every second patient as compared to genomic sequencing data alone. The outcome was assessed in 152 patients and showed a Disease Control Rate (DCR) of 58.6% and an Objective Response Rate (ORR) of 27.0%. One in three patients (32.8%) showed at least a 50% longer PFS with molecularly guided therapy compared to the previous standard therapy. Notably, 86.4% of the MTB‐driven implementations were off‐label. CATCH highlights the impact of whole‐genome/exome in combination with RNA sequencing to detect clinically relevant biomarkers in mBC. Omics‐guided targeted therapy in a real‐world setting allows high treatment implementation rates yielding outcome benefit for one‐third of the patients.

## Graphical abstract

What's new?

CATCH is a prospective precision oncology registry trial that harnesses whole‐genome/exome‐ and RNA‐sequencing to enable actionable biomarker detection in metastatic breast cancer patients of any subtype. The data of the first 412 consecutive patients with completed follow‐up in this real‐world, single‐center patient cohort provide robust evidence that comprehensive molecular profiling substantially improves treatment response and progression‐free survival. The integrated profiling approach broadened treatment recommendations beyond those from conventional single‐gene mutation scoring. The reported performance of the CATCH platform underscores the feasibility and clinical benefit of tailoring diagnostic and therapeutic strategies in metastatic breast cancer through precision oncology.

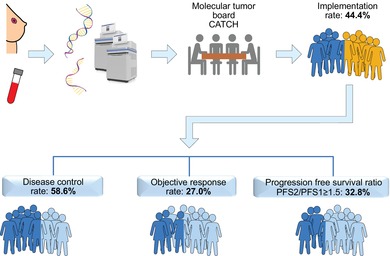

**Abbreviations**
CATCH: Comprehensive assessment of clinical features and biomarkers to identify patients with advanced or metastatic breast cancer for marker‐driven trials in humans
COGNITION: Comprehensive assessment of clinical features, genomics and further molecular markers to identify patients with early breast cancer for enrolment in marker‐driven trials
COGNITION‐GUIDE: Comprehensive assessment of clinical features, genomics and further molecular markers to identify patients with early breast cancer for enrolment on marker‐driven trials—genomics‐guided targeted post‐neoadjuvant therapy in patients with early breast cancer
CR: complete response
DCR: disease control rate
ECOG: Eastern Cooperative Oncology Group
EMA: European Medicines Agency
ER: estrogen receptor
ESCAT: ESMO Scale for clinical actionability of molecular targets
GHGA: The German Human Genome‐Phenome Archive
H&E: hematoxylin and eosin
HBOC: hereditary breast and ovarian cancer
HER2: human epidermal growth factor receptor 2
HR: hormone receptor
HRD: homologous recombination deficiency
ICF: informed consent form
IHC: immunohistochemistry
Indels: small insertions/deletions
IRB: institutional review board
LOH: loss of heterozygosity
LST: large scale state transition
mBC: metastatic breast cancer
MSI: microsatellite instability
MTB: molecular tumor board
NCT: National Center for Tumor Diseases
NGS: next‐generation sequencing
ORR: objective response rate
PD: progressive disease
PD‐L1: programmed death‐ligand 1
PFS: progression free survival
PR: partial response
QC: quality control
RCT: randomized controlled trial
RNA‐Seq: RNA sequencing
sCNAs: somatic copy number alteration
SD: stable disease
SNV: single nucleotide variant
SV: structural variant
TAI: telomeric allelic imbalance
TIL: tumor‐infiltrating lymphocyte
TMB: tumor mutational burden
TNBC: triple negative breast cancer
WES: whole exome sequencing
WGS: whole genome sequencing

## 1 INTRODUCTION

Metastatic breast cancer (mBC) still accounts for a dismal prognosis and constitutes a leading cause of tumor‐related disease burden among females with approximately 685.000 deaths per year worldwide.^1^ Significant improvements in the clinical management of mBC have been accomplished in the last decade with a prolongation of overall survival to 3–5 years largely attributed to novel drug classes such as CDK4/6 inhibitors and antibody‐drug‐conjugates.^2^, ^3^, ^4^ However, depending on prognostic factors, still 10–30% of all breast cancer patients do not survive the 10‐year period after diagnosis due to metastatic dissemination and the corresponding ongoing evolutionary drift driving therapy resistance.^5^

Conventional stratification schemes such as subtype classification by hormone receptor and human epidermal growth factor 2 (HER2) expression and TNM staging provide a robust clinical backbone. Several biomarker‐driven systemic therapies have recently received regulatory approval based on high‐level evidence from randomized controlled trials (e.g., biomarkers *BRCA1/2*, *PIK3CA/AKT1/PTEN*, *ESR1*).^6^, ^7^, ^8^, ^9^, ^10^, ^11^, ^12^, ^13^ Others—like *TACSTD2* (encoding TROP2)—are discussed as having predictive value for certain patient populations, even if they are not currently relevant for the label.^14^ However, despite serving as a precious source for broad clinical application, the respective trials deliver limited information on the highly heterogenous molecular basis of the disease. Comprehensive next‐generation sequencing (NGS) empowers broad and unbiased interrogation of the genome and transcriptome aiming at the identification of predictive biomarkers. Precision oncology concepts encompassing complex genomic testing allow tackling the multifactorial disease etiology and clinical outcomes.

Recently, the largest real‐world clinical whole genome sequencing (WGS) study uniquely provided descriptive integrated clinical and genomic data from 13.880 patients of the UK National Health Service within the 100.000 Genomes Project from 33 tumor entities.^15^ Beyond constituting a feasibility proof‐of‐principle for broad clinical adoption, the strategy unraveled pan‐genomic prognostic and predictive biomarkers. Beyond these translational research‐oriented analyses, further tumor‐agnostic prospective frameworks in advanced solid tumors, such as the MOSCATO‐01^16^ and NCT MASTER^17^ trials, underscore the considerable potential to improve progression‐free survival (PFS) by directing genome‐informed therapies into the clinics.

During the last decade, breast‐cancer‐directed studies, such as the non‐interventional, AURORA initiative (381 mBC patients), underlined the power of NGS‐based approaches to delineate enriched drivers in metastatic lesions.^6^ From the clinical perspective, the randomized SAFIR02‐BREAST phase II trial served as a conceptual blueprint comparing a set of targeted therapies matched to genomic biomarkers to standard‐of‐care chemotherapy in 238 HER2‐negative mBC patients demonstrating improved outcome.^18^ The protocol focused on patients in early treatment lines eligible for 1st/2nd line of chemotherapy with response after 6–8 cycles of induction chemotherapy. Within the evidence level framework of genome‐based actionability, targeted substances matched to genomic alterations in the ESMO Scale for Clinical Actionability of molecular Targets (ESCAT) category I/II, displayed superior progression‐free survival compared to standard maintenance chemotherapy (adjusted hazard ratio 0.41, 90% confidence interval 0.27–0.61, *p* < 0.001).

While these landmark initiatives emphasize the clinical utility and efficacy of personalized genomics‐guided frameworks in mBC, to date there is still a substantial gap of knowledge on the application of these approaches into the ‘real‐world’ clinical setting. How to define the optimal patient population, appropriate biomarkers or the timing of analysis in routine clinical care is largely unknown but of great clinical relevance. Further, there is an unmet need to ease access for broader breast cancer populations to evidence‐based precision oncology platforms encompassing both, broad diagnostics and targeted drug repertoires in respective health care structures.

Targeted compounds hold the potential to provide favorable outcomes in patient populations with specific biomarker profiles, requiring in‐depth tumor profiling to define the optimally stratified patient population. Recently, cumulative randomized, controlled trials (RCTs) have failed to show an advantage in favor of targeted treatment. Because these trials used broad inclusion criteria or lacked proper biomarker stratification, it remains unclear which patients, if any, will benefit from targeted therapy options.^19^, ^20^, ^21^, ^22^ Hence, there is a substantial requirement to identify within real‐world‐evidence frameworks patient subcohorts with positive and negative predictive biomarkers, which profit from personalized oncology approaches.

The CATCH (Comprehensive assessment of clinical features and biomarkers to identify patients with advanced or metastatic breast cancer for marker driven trials in humans) precision oncology platform incorporates comprehensive multidimensional molecular profiling of breast cancer metastases by whole‐genome‐/whole‐exome‐sequencing (WGS/WES) as well as transcriptome sequencing (RNA‐Seq) to identify biomarkers and actionable alterations in mBC patients irrespective of tumor subtype, therapy type and treatment line.^23^ Beyond setting up a prospective diagnostic platform on the basis of next‐generation sequencing, a major goal resides in co‐evolving medical treatment settings, which either encompass subsequent (i) interventional clinical trial enrolment, (ii) refinement of approved standard‐of‐care agents (*in‐label use*) or (iii) targeted substances approved outside the indication (*off‐label use*). We here present our experience with the first 412 patients with completed follow‐up in the CATCH trial.

## 2 MATERIALS AND METHODS

### 2.1 Patients and study

We here report data of the first 558 patients consecutively recruited into CATCH from June 2017 to October 2021 at the Division of Gynecological Oncology at the National Center for Tumor Diseases (NCT) Heidelberg. Patients were enrolled either at first diagnosis of primary metastasized BC or at the time of disease progression. Inclusion was independent of BC subtype, patient age, gender, treatment line and metastatic location. As this is a prospective real‐world study of consecutive patients in a single center, stratifying patients according to molecular profiles of their respective tumors, group assignment, randomization or power calculations do not apply.

Patients had to have an Eastern Cooperative Oncology Group (ECOG) score of 0 or 1 and a life expectancy of at least 3 months. At least one metastatic or unresectable locoregional tumor site accessible for biopsy at the time of enrolment was mandatory. In patients with progressive disease at both the locoregional site and at distant metastases, it was at the investigator's discretion to decide which site to biopsy. After enrolment, the CATCH tumor biopsy was performed in conjunction with a tumor biopsy required for standard treatment planning. During the diagnostic workup of the CATCH biopsy and discussion of the patient's case in MTB, standard treatment—hereafter also called bridging treatment—was administered and continued until it had to be discontinued for clinical reasons. Leading clinical reasons were disease progression or intolerable toxicity. Once standard (‘bridging’) treatment had to be stopped, the patient was assessed to see if a tumor‐specific therapy recommended by the MTB could be started instead of the next standard line of treatment. Only the first such implementation was analyzed for the study presented here. A patient was allowed to have received more than one bridging line of treatment before a therapy recommended by MTB was implemented. Formally, a bridging treatment was not mandatory but due to the nature of the disease, it was done for the vast majority of cases. Details of the clinical workflow were published previously.^23^

### 2.2 Analysis of clinical data and outcome measures

We here focused on three main clinically relevant outcome measures: treatment implementation rate, response rates to implemented targeted treatments, and intra‐patient PFS ratio—all of which are detailed in the following. The rate of implementation of MTB recommended treatments was calculated by dividing the number of patients, who started the treatment recommended by the MTB, by the total number of patients discussed in the MTB. In those cases that received more than one treatment recommended by the MTB only, the first one was counted. The further main outcome measure for the current analysis was treatment response. Response was assessed every 2–3 months by relevant imaging, tumor markers and/or clinical examination as judged by the treating physician. Response status (progressive disease (PD), stable disease (SD), partial response (PR) and complete response (CR)) was assessed at each restaging interval. To compare treatment outcomes, disease control rate (DCR; sum of SD, PR, CR divided by total number of patients) and objective response rate (ORR; sum of PR, CR divided by total number of patients) were calculated. The best response achieved at least once during the respective treatment line was used to calculate DCR and ORR. To compare the clinical outcome for molecular‐guided treatment versus the prior non‐genome‐guided treatment on a patient individual basis, we calculated the progression‐free survival ratio (PFS2/PFS1). PFS2 was defined as PFS on MTB‐recommended therapy while PFS1 represents PFS on the immediately prior standard‐of‐care treatment line of the same patient. In accordance with previous publications,^17^, ^24^ a PFS ratio of 1.5 (i.e., 50% longer on MTB‐recommend than on previous standard therapy) or larger was defined as positive.

The MTB considered both European Medicines Agency (EMA) approved (in‐label) and unapproved (off‐label) therapies for mBC or mBC subtype. As approval status might affect the probability of implementation and might change during the time from MTB to the next progression of disease, the approval status at the time of implementation was used for the analysis performed here.

We also distinguished whether a targeted therapy recommended by the MTB was ‘MTB‐driven’ or ‘same as clinical standard’. While an ‘MTB‐driven’ therapy would not have happened without testing within CATCH, an MTB recommendation categorised as ‘same as clinical standard’ may have happened at the time even without CATCH molecular profiling and MTB discussion. For example, the use of a PARP inhibitor in a patient with a somatic BRCA1 mutation detected by CATCH was treated as ‘MTB‐driven’ because somatic BRCA1 testing is not covered by health insurance in Europe but only approved for patients with a germline BRCA1/2 mutation. On the other hand, giving a PARP inhibitor to a (HER2‐negative) patient found to have a germline BRCA1 mutation in CATCH was judged to be ‘in line with the clinical standard’ as both testing and treatment are covered, approved and recommended by national and international health authorisation and guideline committees.

Investigators handling patient material or generating molecular profiles were fully blinded.

### 2.3 Genomic and transcriptomic analysis

Processing of tumor and control specimens as well as WGS/WES and RNA‐seq procedures are detailed in Hlevnjak et al. and Horak et al.^17^, ^23^ Quality statistics for whole genome sequencing, whole exome sequencing and RNA sequencing data are presented in Table S3. Given the urgent clinical need to analyze RNA samples in this real‐world study, we adopted less stringent quality criteria for sequencing than typically applied (e.g., RIN, 28S/18S ratio, or DV200). Instead, we included samples with ≥40 million uniquely mapped reads and performed rigorous variant assessment for libraries with complexities below 25 million reads (e.g., evaluating potential under expression or missed gene fusion events).

### 2.4 Bioinformatic and statistical analyses

WGS and WES data of tumor samples and blood (as a proxy for the germline situation) were analyzed to identify somatic genomic alterations including single nucleotide variants (SNVs), indels, structural variants (SVs), and somatic copy number alterations (sCNAs) using established bioinformatic pipelines (BWA‐mem (https://github.com/DKFZ-ODCF/AlignmentAndQCWorkflows), SAMtools/bcftools (https://github.com/DKFZ-ODCF/SNVCallingWorkflow), Platypus (https://github.com/DKFZ-ODCF/IndelCallingWorkflow), SOPHIA (https://github.com/DKFZ-ODCF/SophiaWorkflow), ACEseq (https://github.com/DKFZ-ODCF/ACEseqWorkflow), CNVkit). Genomic instability metrics (homologous recombination deficiency [HRD], loss of heterozygosity [LOH], large scale state transitions [LSTs] and telomeric allelic imbalances [TAIs]) were calculated from sCNA data. Microsatellite instability was assessed using MSISensor (RRID: SCR_006418), and mutational signatures were deconvolved with YAPSA (https://bioconductor.org/packages/YAPSA/). RNA‐seq data was aligned with STAR, gene expression quantified using a custom BEDtools‐based script (https://github.com/DKFZ-ODCF/RNAseqWorkflow), and gene fusions identified with Arriba (RRID: SCR_025854). Detailed pipeline parameters, version numbers, and data integration methods are described in Horak et al.^17^ The sequencing coverage and quality statistics for each sample are summarized in Table S3.

### 2.5 Prioritization of molecular findings

The variant classification in precision oncology published by Leichsenring et al. was applied.^25^ Of the four evidence‐based classes m1‐m4, we relied on the top priority levels m1 and m2 in the vast majority of cases.

## 3 RESULTS

### 3.1 Outline of the study

Between June 2017 and October 2021, the CATCH study recruited 558 patients with metastatic breast cancer (mBC) irrespective of subtype and therapy line. All information on patients' attrition is presented in Table 1 and further detailed in Figure S1 (drop outs prior to tissue acquisition [*n* = 34]: screening failure [*n* = 6], biopsy accessibility [*n* = 3], clinical deterioration [*n* = 7], death [*n* = 5], patient wish: no biopsy [*n* = 5], consent withdrawal [*n* = 5], diagnostic redirection [*n* = 1], quality control [*n* = 1], other [*n* = 1]; drop outs MTB [*n* = 112]: screening failure [*n* = 1], biopsy accessibility [*n* = 5], clinical deterioration [*n* = 8], death [*n* = 31], patient wish: no biopsy [*n* = 3], consent withdrawal [*n* = 1], diagnostic redirection [*n* = 19], tumor cell content/QC [*n* = 41], other [*n* = 3]). With 409 females and three males (see Table 1), gender distribution matched the value expected in any breast cancer patient cohort enrolling patients consecutively. The median age at first diagnosis and at study entry was 48 and 55 years, respectively (for details see Table 1: ranging from 23 to 80 years and 24 to 83 years, respectively).

**TABLE 1:** Description of patient cohort.

Patients	*n*	% or (range)
*MTB cohort* (*n = 412*)		
Age 1st diagnosis (median)	48	(23–80)
Age study entry (median)	55	(24–83)
Gender		
Female	409	99.3
Male	3	0.7
Menopausal status at study entry		
Pre/peri menopausal	121	29.4
Post‐menopausal	268	65.0
Unknown	23	5.6
ECOG at study entry		
ECOG 0 or 1	382	92.7
ECOG 2 or 3	22	5.3
Unknown	8	1.9
Subtype of primary breast cancer^a^		
HRpos HER2neg	211	51.2
TNBC	83	20.1
HER2pos	52	12.6
Missing	66	16.0
*Treatment implementation cohort (n = 183)*		
Menopausal status at study entry		
Pre/peri menopausal	61	33.3
Post‐menopausal	115	62.8
Unknown	7	3.8
ECOG at study entry		
ECOG 0 or 1	173	94.5
ECOG 2 or 3	7	3.8
Unknown	3	1.6
Subtype of metastatic lesion		
HRpos HER2neg	105	57.4
TNBC	62	33.8
HER2pos	16	8.7
Treatment line at trial entry		
Primary mBC or after 1st line	116	63.4
After 2nd or 3rd line	42	23.0
After 4th line	25	13.7
Treatment line at time of treatment implementation		
Primary mBC or after 1st line	3	1.6
After 2nd or 3rd line	103	56.3
After 4th line	77	42.1

^a^ For subtype of metastases see Figure 1C.

**FIGURE 1:**
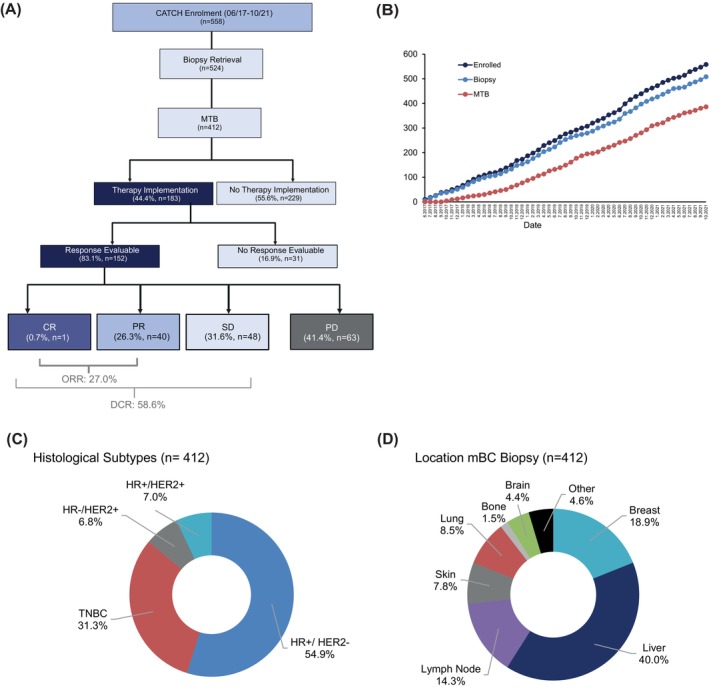
Patient cohort. (A) The consort diagram illustrates the streamlined patient recruitment (06/17 to 10/21) process to the molecular tumor board (MTB), treatment implementation rate and treatment outcome of the CATCH study cohort. (B) The number of patients who signed the Informed Consent Form (ICF), underwent tumor tissue sampling for molecular analysis and were discussed at the MTB is plotted over time until recruitment cut‐off (10/21). Ring plots of the MTB cohort (*n* = 412) illustrate their histological subtypes (C) and the distribution of anatomical sites from which tumor biopsies were obtained (D).

Tumor biopsies were collected from 524 participants from progressive lesions at initial diagnosis of metastasis or at the time point of progression (Figure 1A). A median of 10 patients was recruited per month, of which nine were biopsied and seven were discussed in the MTB (Figure 1B). A total of 112 patients dropped out, either because of poor health or because the tissue quality—predominantly attributed to low tumor cell content—was deemed too poor for molecular profiling assays. Finally, tumors from 412 patients (thereafter called MTB cohort) and matching blood as proxy for germline alterations were subjected to WGS/WES and, whenever possible, to RNA‐sequencing. Patients were discussed in the MTB of the National Center for Tumor Diseases (NCT) Heidelberg on a weekly basis to prioritize actionable alterations and biomarkers. Subsequent analyses refer to this MTB cohort (Table 1), unless otherwise stated. The median age of the patients at study entry was 55 years (range 24–83 years). Three male patients were included and about one third of the female patients were pre‐ or peri‐menopausal (*n* = 121, 29.4%). An ECOG performance status of 0 or 1 was assigned to 382 (92.7%) participants. At the time point of enrolment approximately one in two patients (*n* = 226; 54.9%) was diagnosed in the profiled lesion with HR‐positive/HER2‐negative (including HER2‐low), as determined by biopsy at study entry. Triple‐negative mBC (TNBC) and HER2‐positive mBC were diagnosed in 129 (31.3%) and 57 (13.8%) patients, respectively (Figure 1C).

After enrolment tumor tissue was obtained from a clinically relevant mBC metastasis. For patients with both, local recurrence and distant spread, and for patients with primary mBC, it was the responsibility of the treating physician to select either the local tumor or its distant spread for biopsy. Liver (*n* = 165; 40.0%), breast (*n* = 78; 18.9%) and lymph nodes (*n* = 59; 14.3%) were the most common anatomical sites sampled for the study (Figure 1D).

When patients had to change their standard anticancer treatment, following the next standard‐therapy line, mainly because of treatment‐related toxicity (*n* = 8) or disease progression (*n* = 162), 183 patients were switched to a molecular‐guided anticancer treatment resulting in an implementation rate of a molecular‐guided therapy of 44.4% (183/412). Treatment response was evaluable in 83.1% (152/183) patients. 89 showed a measurable response with one complete remission (CR), 40 partial responses (PR) and 48 disease stabilisations (SD) (Figure 1A). Overall, the disease control rate (DCR with CR + PR + SD) was 58.6% (89/152) and the objective response rate (ORR with CR + PR) was 27.0% (41/152).

### 3.2 Biomarkers used for prioritization in Molecular Tumor Boards

**FIGURE 2:**
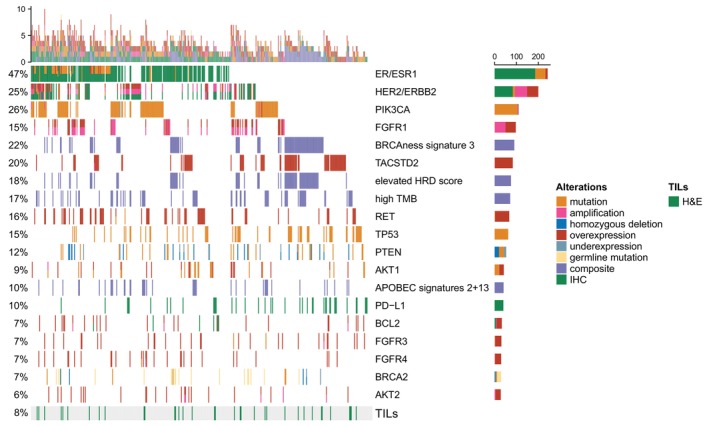
Landscape of molecular alterations constituting biomarkers for the molecular tumor boards. Shown are only biomarkers that were used for the top three recommendations and present in more than 5% of patients (*n* = 412). Each column represents an individual patient. Biomarkers are further split according to the exact type of sequencing alteration, including also those obtained by immunohistochemical staining. Composite alterations refer to DNA sequencing‐derived biomarkers which are not single gene‐based (tumor mutational burden, homologous‐recombination deficiency (HRD) score and single‐base substitution mutational signatures). Percentages on the left represent the fraction of patients with a particular biomarker; vertical bars on top depict the number of supporting biomarkers in an individual patient; horizontal bars on the right depict the number of patients with a given alteration type for the respective biomarker; bar at the bottom depicts tumor samples positive for tumor‐infiltrating lymphocytes based on hematoxylin and eosin staining. H&E, hematoxylin and eosin; HRD, homologous recombination deficiency; IHC, immunohistochemistry; SBS, single base substitution; TILs, tumor‐infiltrating lymphocytes; TMB, tumor mutational burden.

In addition to routinely accessible IHC staining of ER, HER2 and PD‐L1, the majority of biomarkers supporting clinical actionability were DNA and/or RNA sequencing‐based (Figure 2, showing biomarkers for top three recommendations per case). When split according to exact alteration type, disregarding copy number and expression‐based alterations, the most common actionable oncogenic mutations were found in *PIK3CA* (25.5%, *n* = 105), followed by those in *ESR1* (11.4%, *n* = 47), *AKT1* (5.1%, *n* = 21) and *ERBB2* (2.4%, *n* = 10) genes. With the exception of TP53 genomic loss‐of‐function alterations, which display only a limited predictive value and were not directly actionable in one patient (0.2.%), *PTEN* (11.7%, *n* = 48) and *BRCA2* (6.8%, *n* = 28) were the most frequently altered tumor suppressors in our cohort that could be successfully addressed clinically via AKT‐ or mTOR‐, and PARP‐inhibition, respectively.

**FIGURE 3:**
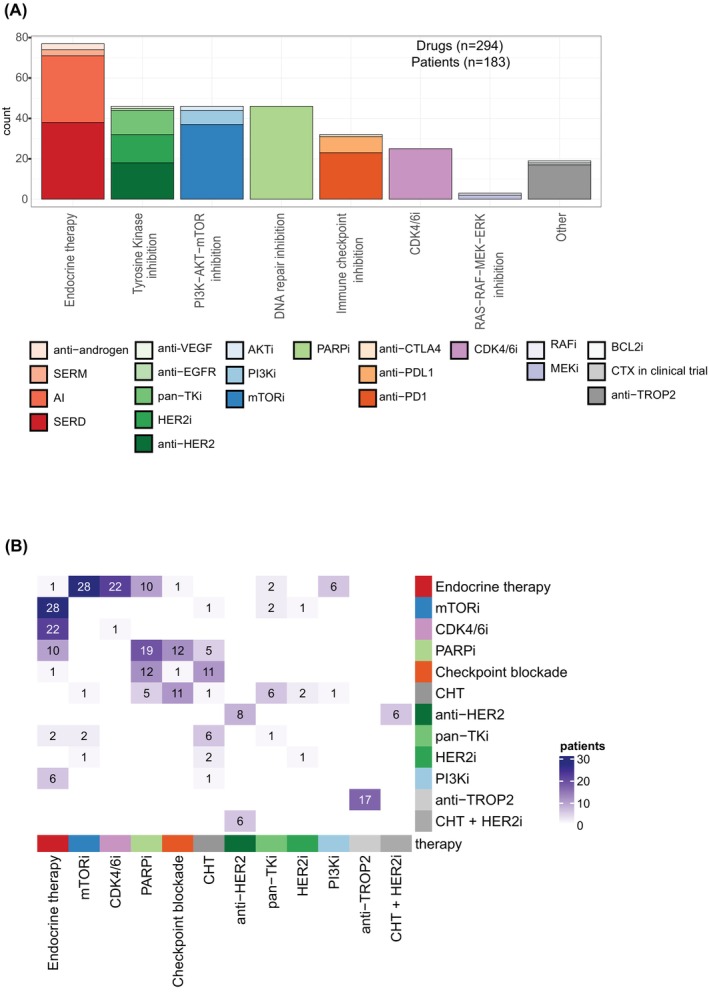
Allocation of the drugs implemented in CATCH. (A) Allocation of administered drugs (*n* = 294) with respect to affected pathways or therapy types, differentiating between “Endocrine therapy”, “Tyrosine kinases inhibition”, “PI3K‐AKT‐mTOR inhibition”, “DNA repair inhibition”, “Immune Checkpoint Inhibition”, “CDK4/6i”, “RAS‐RAF‐MEK‐ERK inhibition “and “Other”. In addition, the drugs have been subclassified into their specific mechanisms of action. (B) Heat map showing the frequency distribution of the drug classes used in mono‐ and combination therapies, both doublet (115/183 [62.8%]) and triplet (16/183 [8.8%]) combinations. Diagonal shows mono and combination therapies of the same therapy group. CHT, chemotherapy.

Overall, standard anti‐HER2 treatments driven by biomarkers (25%) (see Figure 2) were expectedly recommended based on (i) amplifications accompanied by mRNA overexpression and positivity on immunohistochemistry, (ii) HER2 positivity on IHC alone, and (iii) *ERBB2* mutations and amplifications. In cases with amplifications of *FGFR1* accompanied by mRNA overexpression (8.3%, *n* = 34) either multi‐kinase or FGFR‐specific inhibitors were recommended. RNA expression can be quite helpful in assessing the potential role of a given copy number alteration. Accordingly, we often used overexpression of actionable targets alone or in combination with consecutive IHC staining to support MTB recommendations of diverse treatment modalities. These range from antibody‐drug conjugates for *TACSTD2* (TROP2) overexpression, multi‐kinase inhibitors for *RET*, *FGFR3* and *FGFR4* overexpression, to small molecule inhibitors of anti‐apoptotic proteins for *BCL2* overexpression. Of note, many well‐described and frequently recurring oncogenic alterations, such as *MYC* amplification and/or overexpression, are absent from our biomarker summary oncoplot (see Figure 2), which is primarily due to lack of direct actionability and appropriate drugs, effectively resulting in sparsity of related recommendations. Apart from single‐gene based biomarkers and alterations, WGS and WES allowed us also to assess and quantify several computationally derived composite biomarkers such as mutational signatures (BRCAness signature 3, 21.8%, *n* = 90 recommendations; APOBEC signatures 2 + 13, 10.0%, *n* = 41 recommendations), homologous recombination deficiency (HRD) scores (18.2%, *n* = 75 recommendations) and tumor mutational burden (TMB) (17.2%, *n* = 71 recommendations), which further expanded treatment options for up to 36.9% of patients (*n* = 152) considering the top three treatment recommendations (see Figure 3).

We observed a remarkable rate of patients carrying constitutional pathogenic or likely pathogenic variants in established cancer predisposition genes (16.7%, *n* = 69). Of these, 56 were within the group of hereditary breast and ovarian cancer (HBOC) genes with *ATM* (*n* = 4), *BARD1* (*n* = 1), *BRCA1* (*n* = 16), *BRCA2* (*n* = 24), *CHEK2* (n = 7), *PALB2* (*n* = 3) and *RAD51C* (*n* = 1), while 13 were within other known tumor predisposition genes: *MLH1* (*n* = 1), *MN1* (*n* = 1), *PMS2* (*n* = 1) and *MUTYH* (het) (*n* = 10). All of these patients were referred to genetic counseling.

### 3.3 Implemented therapies

Among the 183 implemented therapies, 52 (28.4%) were mono‐ and 131 (71.6%) were combination therapies. Specifically, doublet combinations constituted 115 (62.8%), and triplet combinations accounted for 16 (8.7%) (see Figures 3B and S2). The 183 therapies included a total of 294 drugs of the following pathway categories: Endocrine therapy (77 drugs in 77 patients, 42.1%), tyrosine kinase inhibition (46 drugs in 39 patients, 21.3%), modulation of the PI3K‐AKT–mTOR signaling pathway (46 drugs in 46 patients, 25.1%), targeting of DNA repair mechanisms (46 drugs in 46 patients, 25.1%), immune checkpoint blockade (32 drugs in 31 patients, 16.9%), CDK4/6 inhibition (25 drugs in 25 patients, 13.7%), modulation of the RAS/RAF–MEK–ERK signaling pathway (3 drugs in 2 patients, 1.1%), other mechanisms (18 drugs in 18 patients, 9.8%) and one patient with chemotherapy in a clinical trial. Figure 3 shows the distribution of the different drugs. Figure 3 panel B depicts the drug classes used in therapies (for at least two patients), with monotherapies (52/183 [28.4%]), doublet (115/183 [62.8%]) and triplet (16/183 [8.7%]) combinations. Moreover, in some cases chemotherapy‐backbone was used as a combination partner in conjunction with targeted therapy (*n* = 36 patients, 19.7%, see Figure S2).

### 3.4 Clinical outcome

**FIGURE 4:**
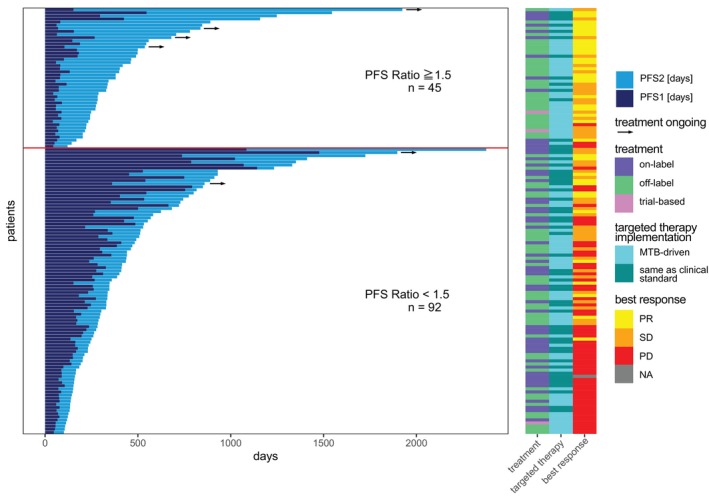
Clinical outcome assessed by progression‐free survival. Illustration of the progression‐free survival (PFS) ratios of 137 patients eligible for clinical outcome measurements. PFS ratio was defined as PFS2/PFS1 where PFS2 is PFS on molecular tumor board (MTB) recommended therapy and PFS1 for treatment immediately preceding MTB treatment. Patients are sorted by their descending PFS ratio, defined as ≥1.5 (*n* = 45) to be beneficial with data assessed in cumulative days for PFS1 and PFS2. Ongoing treatments are denoted by arrows. Adjacent to each patient entry, details on the treatment's labeling status (in‐label/off‐label/trial‐based), type of implemented targeted therapy (MTB‐driven/same as clinical standard), and best response observed (Partial Response [PR], Stable Disease [SD], Progressive Disease [PD], Not Applicable [NA]) are provided).

To compare the clinical outcome for molecular‐guided treatment versus the prior standard treatment, we calculated the PFS2/PFS1 ratio from 137 patients evaluable for clinical outcome measurements (15 were excluded due to incomplete information on therapy or progress, no therapy and/or short time interval [<6 weeks] prior to MTB) (see Figure 4). PFS2 was defined as PFS on MTB‐recommended therapy while PFS1 is PFS on the patient's immediately prior standard‐of‐care treatment line. In 32.8% of patients (*n* = 45) the PFS2 was at least 50% longer than PFS1 indicating a therapeutic benefit for MTB‐recommended treatment. Among patients with PFS ratio above 1.5, 93.3% (42/45) achieved clinical benefit (DCR, ORR), compared to only 40.7% (37/91) among those with PFS ratio below 1.5 (*p* = 8.4 × 10^−10^, two‐sided Fisher's exact test). Furthermore, six patients were still receiving MTB‐recommended treatment at the time of data cutoff (Figure 4, denoted with arrow).

### 3.5 Comparison of clinical outcomes between subgroups of implemented targeted therapies

**FIGURE 5:**
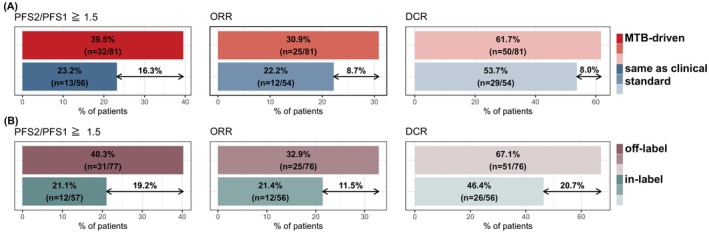
Comparison of outcomes between different subgroups of implemented targeted therapies (*n* = 137). Comparison of case numbers of MTB‐driven vs. the same as clinical standard (panel A), and off‐label vs. in‐label targeted therapies (panel B). Outcomes (left: Proportion of patients with PFS2/PFS1 ratio ≥1.5; middle: ORR; right: DCR; colored with increasing transparency). For statistical assessment see Table S1.

In‐label and/or clinical standard targeted therapy options were frequently recommended by the MTB, especially if patients were profiled very early on in the course of their metastatic disease. Details of respective therapy implementations and their outcomes are presented in Figure 5 and Table S1. Restricting our analyses to the patients with evaluable PFS ratios (*n* = 137) we distinguished targeted therapy implementations that were MTB‐driven and would not have been implemented in the clinical routine, from those which could have been implemented as clinical standard at the time even without CATCH molecular profiling and MTB discussion. Notably, 59.1% (*n* = 81) of our targeted implementations were MTB‐driven. While 57 (41.6%) patients were treated in‐label and 77 (56.2%) off‐label, three patients were enrolled in clinical trials. Most interestingly, 86.4% (70/81) of the MTB‐driven implementations were off‐label. More importantly, when comparing these groups with respect to different outcome measures, we found that targeted implementations that were exclusively MTB‐driven numerically outperformed those given as clinical standard across all outcome measures, with the strongest signal for the difference in proportion of patients with PFS2/PFS1 ratios above 1.5 (16.3% difference, *p* = 0.070, Figure 5A and Table S1). Similarly, comparison of off‐label versus in‐label targeted implementations expectedly showed the same trend; however, here even with statistically significant difference in both the proportion of patients with PFS2/PFS1 above 1.5 (19.2% difference, *p* = 0.030) and DCR (20.7% difference, *p* = 0.028, Figure 5B and Table S1).

## 4 DISCUSSION

To guide clinical decision‐making for patients with advanced breast cancer, we have established the CATCH molecular analysis platform. Within CATCH, comprehensive genomic and transcriptomic profiling of clinically relevant breast cancer metastasis upon disease progression is being performed. Almost every second patient (*n* = 183; 44.4%) out of the 412 mBC cases discussed in the MTB received a targeted treatment recommended by the MTB during the subsequent course of their disease. Overall, 152 cases were available for evaluation of treatment response in terms of DCR and ORR. The disease control rate was 58.6% and the objective response rate 27.0%. Approximately one in three (32.8%) patients benefited in terms of PFS ratio (PFS2/PFS1 ratio ≥1.5). In 16.7% (*n* = 69) of the cases analyzed, mutations in inherited cancer predisposition genes with known pathogenic relevance—the basis for recommending genetic counselling—were found. These findings are overall consistent with the data obtained at the initial phase of CATCH,^23^ but underline the power of broad genomic profiling to detect further variants given current literature evidence based on multigene panel sequencing detecting about 10% of germline variants.^26^ Although cross‐study comparisons are difficult due to differences in study populations and design, other solid tumor cohorts reported results similar to CATCH in terms of treatment response. For example, TOP‐GEAR and MASTER studies investigating molecularly guided therapy in solid tumors, including but not limited to breast cancer, found an ORR of 20% and 24%, respectively.^17^, ^27^ Potential measures to further optimize current molecular precision oncology settings are discussed in the following.

Therapy recommendations were informed by at least one genomic aberration or biomarker in 93.0% of patients (383/412), including mutations, amplifications, homozygous deletions, fusions, and composite biomarkers like mutational signatures, tumor mutational burden (TMB), microsatellite instability (MSI) and homologous recombination deficiency (HRD) scores. Recent studies based on the restricted explanatory power of selective mutation scoring, when using confined gene panels only, reported recommendations in 51% of the patients when applying comparable ESCAT tier I plus tier II scores in the AURORA trial^6^ and 44.2% when scoring patients within the screening platform for SAFIR02‐BREAST that presented a targetable genomic aberration.^18^ As expected from the literature, the most common actionable oncogenic mutations identified were in *PIK3CA*, *AKT1, ESR1, BRCA1/2* and *PTEN*, which are also covered by most commercially available diagnostic gene panels. However, as WGS and WES generate much more homogeneous data, scoring and quantifying of computationally derived composite biomarkers were greatly facilitated (homologous recombination deficiency (HRD) scores and tumor mutational burden (TMB)), or complemented by additional information (MSI scores, mutational signatures). Incorporating the information of composite markers into MTB recommendations led to additional treatment recommendations for 36.9% (152/412) of patients. By analyzing the transcriptome of biopsied metastases, we expanded the number of treatment options even further, analogous to previously published observations.^28^ The latter showed that the combination of WGS/WES and RNA‐Seq identified 1/3rd of biomarkers not covered by a panel. Gene expression data were utilized to guide treatment decisions in 53.8% of patients (300/558).^28^ Among patients receiving recommendations based on gene expression, 44.7% (678/1517 total recommendations) were directed to clinical trials.^28^

The low treatment implementation rate of many real‐world precision oncology programs makes data interpretation as well as comparability of trials and treatment outcomes more difficult. Through the CATCH platform, we were able to implement genomics‐guided treatment in 44.4% of patients. This rate is significantly higher than previously reported in the literature from other real‐world precision oncology registry trials. AURORA, one of the largest genomic registry trials in breast cancer, reported an implementation rate of 7% (25 out of 379 patients) regarding therapies matched to genomic alterations. However, this comparison needs to be made with caution, as CATCH, unlike AURORA, has been a single‐institution study and only became multicentric after the cohort reported here was fully recruited. In addition, when comparing international precision oncology trials, differences between the German and other healthcare systems need to be considered. Still, even when comparing CATCH to other German initiatives that tracked treatment implementation after their institutional MTBs, that is, 16%^29^ and 29%,^30^ our implementation rate is substantially higher. This may be due to the fact that CATCH included patients on earlier lines of treatment, whereas many other MTBs require standard lines of treatment to be exhausted in order to be reimbursed for molecular testing.

To assess the impact of precision oncology studies, it is of particular interest to which extent off‐label treatments became in‐label later on, demonstrating the implementation of improved treatments early on. In fact, 11.8% of the off‐label implementations in the here reported study period would be in‐label today. Moreover, if one considers also targeted treatments that are currently still off‐label in the EU, but were approved by the FDA, or for which clinical trials became available in Germany in the meantime, one would reach 33.3% of patients who might benefit from receiving treatments, for which molecular profiling is essential. Notable examples among these would be targeting HER2 mutations with trastuzumab + tucatinib ± fulvestrant,^31^ using pembrolizumab in tumors with high tumor mutational burden,^32^ or using PARP inhibitors in tumors with somatic BRCA1/2 or germline PALB2 mutations,^33^ as well as combining endocrine therapy with PARP inhibitors in BRCA‐mutated and hormone receptor positive tumors (currently, phase II ELEMENT study, NCT06201234).

Though PFS ratio as well as treatment response proved notably beneficial, we were not yet able to observe overall survival benefit for MTB‐guided treatment. On first sight, this appears to be due to the intrinsic heterogeneity of the study cohort consisting of different BC subtypes, treatment lines and metastatic patterns. Even in SAFIR‐02, one of the currently most comprehensive precision oncology intervention trials, prolongation of time to progression was only observed in subgroups such as those treated with olaparib.^18^ Secondly, the lack of availability of innovative but not yet approved drugs outside clinical trials and the legal and financial constraints associated with off‐label use are considered to be the main barriers to translating precision oncology into clinical practice.

Though 412 patients were analyzed, homogeneous patient cohorts with the same biomarker‐informed treatment are still too small to fully judge the long‐term clinical benefit of a particular setting including overall survival rates. Exploration of biomarker‐specific treatment response was, however, conceivable for one implementation subcohort of substantial size, that is, the patients receiving PARP inhibitors (46 patients; 34 were evaluable for PFS ratio, resulting in 26.5% of patients with PFS ratio ≥1.5; 39 patients were evaluable for ORR [30.8%] and DCR [56.4%]). Importantly, PARP inhibitors were implemented as monotherapies in only 41.3% of the cases, whereas the majority are treated by combinations mostly with checkpoint inhibitors or endocrine therapy. We could aggregate predictive biomarkers for PARP inhibitors into two groups: “established” ones (i.e., germline or somatic BRCA1/2 and germline PALB2 inactivating alterations, *n* = 22) that are known to be strongly predictive, and “emerging” ones (excluding germline or somatic BRCA1/2 and PALB2, which are then mostly HRD or BRCAness signature positive, *n* = 24). Comparison of the proportion of patients with PFS ratio ≥1.5 in these two groups revealed 33.3% in the “established” and 18.8% in the “emerging” biomarker group. We observed similar trends for the objective response rate (45.5% vs. 11.8%) and the disease control rate (77.3% vs. 29.4%). Importantly, it seems that some benefit can still be derived from PARP inhibitors even in tumors exhibiting “less” predictive biomarkers only (see Table S2).

The increasingly growing number of recommendations for combinatorial treatments with many different categories as well as the lack of toxicity data for many rational therapy combinations currently further narrows down the implementation rate of genomics‐guided therapies in clinical practice. To overcome such practical limitations, the initiation of novel clinical intervention trials, enrolling patients upon a sequencing‐based stratification, is warranted. The increase in the accessible trial portfolio will improve the recruitment rate and also help to clarify the real impact of molecularly guided therapies on patient outcomes and help to overcome the lack of comparable data reported from the growing number of institutional molecular tumor boards. In addition, by using registry trials like CATCH as a screening platform, recruitment based on early biomarker restricted clinical trials can be accelerated and substantially diminish screening failures based on non‐conclusive biomarker profiles. Such strategies are currently being established worldwide. The multi‐arm interventional trial COGNITION‐GUIDE for the biomarker‐driven treatment of early breast cancer patients^34^ serves as an example in the field of breast cancer. This trial recruits patients upon molecular stratification within the COGNITION registry platform.^35^

## 5 CONCLUSIONS

The reported performance of the CATCH platform underscores the feasibility and clinical benefit of tailoring diagnostic and therapeutic algorithms in metastatic breast cancer. This long‐term, real‐world precision oncology study provides robust evidence that comprehensive molecular profiling can improve treatment response and progression‐free survival. Notably, the integration of whole genome and transcriptome profiling substantially broadened treatment recommendations beyond conventional single gene mutation scoring. These findings collectively affirm the value of precision oncology strategies in optimizing outcomes for patients with metastatic breast cancer.

## AUTHOR CONTRIBUTIONS

**Mario Hlevnjak:** Data curation; formal analysis; methodology; investigation; supervision; writing – review and editing; validation; conceptualization. **Sabine Heublein:** Conceptualization; data curation; formal analysis; visualization; writing – original draft; methodology; investigation; validation; writing – review and editing. **Verena Thewes:** Conceptualization; data curation; formal analysis; visualization; writing – original draft; methodology; investigation; supervision; project administration; writing – review and editing; funding acquisition; validation. **Lukas Wagener:** Conceptualization; data curation; formal analysis; writing – original draft; methodology; investigation; writing – review and editing; validation. **Constantin Pixberg:** Conceptualization; data curation; formal analysis; methodology; investigation; validation; writing – original draft; writing – review and editing. **Carlo Fremd:** Formal analysis; investigation; supervision; writing – review and editing. **Laura Michel:** Formal analysis; investigation; supervision; writing – review and editing. **Christian Maurer:** Data curation; formal analysis; investigation; methodology; validation; writing – review and editing. **Lars Buschhorn:** Data curation; formal analysis; methodology; investigation; writing – review and editing; validation. **Nicola Dikow:** Writing – review and editing; supervision; investigation; formal analysis. **Fangyoumin Feng:** Data curation; formal analysis; methodology; investigation; writing – review and editing; software; validation. **Stefan Fröhling:** Writing – review and editing; methodology; resources. **Christel Herold‐Mende:** Resources; supervision; validation; writing – review and editing. **Steffen Hirsch:** Formal analysis; investigation; writing – review and editing; data curation. **Chen Hong:** Data curation; formal analysis; methodology; investigation; software; validation. **Daniel Hübschmann:** Supervision; writing – review and editing; resources; software. **Lena Jassowicz:** Supervision; writing – review and editing; validation; resources. **Polina Kozyulina:** Software; validation; methodology; data curation; formal analysis; writing – review and editing. **Katrin Pfütze:** Resources; writing – review and editing; project administration; formal analysis. **Richard F. Schlenk:** Formal analysis; writing – review and editing; supervision; resources. **Hans‐Peter Sinn:** Formal analysis; writing – review and editing; methodology. **Katharina Smetanay:** Supervision; writing – review and editing; formal analysis; investigation. **Christoph Springfeld:** Resources; methodology; writing – review and editing. **Albrecht Stenzinger:** Formal analysis; methodology; writing – review and editing. **Celina Wagner:** Data curation; formal analysis; methodology; investigation; writing – review and editing; validation. **Stephan Wolf:** Resources; formal analysis; methodology. **Andreas Trumpp:** Resources; funding acquisition; writing – review and editing. **Dirk Jäger:** Resources; writing – review and editing. **Oliver Zivanovic:** Resources; writing – review and editing. **Marc Zapatka:** Conceptualization; data curation; formal analysis; visualization; writing – original draft; methodology; investigation; supervision; writing – review and editing; software; validation. **Andreas Schneeweiss:** Conceptualization; formal analysis; supervision; writing – review and editing; investigation; funding acquisition; resources. **Peter Lichter:** Conceptualization; formal analysis; writing – original draft; methodology; investigation; supervision; writing – review and editing; resources; funding acquisition.

## FUNDING INFORMATION

This work was supported by the National Center for Tumor Diseases (NCT) Heidelberg, Molecular Precision Oncology Program, NCT proof of concept program, the One NCT Initiative funded by the Bundesministerium für Bildung und Forschung (BMBF), SATURN3 (BMBF, #01KD2206), INTEGRATE‐TN (German Cancer Aid, #70113450) and LITT‐Met‐SMILE (German Cancer Aid, #70116282) projects. LJ was supported by the Faculty of Medicine and the Marsilius‐Kolleg of Heidelberg University.

## CONFLICT OF INTEREST STATEMENT

Mario Hlevnjak declares stock ownership in the following companies: AbbVie, Agilent Technologies, Amgen, Arvinas, Astellas Pharma, AstraZeneca, Bayer, BioInvent International, BioNTech, Bristol‐Myers Squibb, Daiichi Sankyo, Eisai, Eli Lilly, Exelixis, Genmab, Gilead Sciences, GSK, Halozyme Therapeutics, Ideaya Biosciences, Immatics, Immutep, Incyte, Ipsen, IQVIA, Illumina, Johnson and Johnson, Kura Oncology, Merck, Merus, Molecular Partners, MSD, Myriad Genetics, Novartis, Nykode Therapeutics, Otsuka, Pfizer, PharmaMar, PMV Pharmaceuticals, Puma Biotechnology, Qiagen, Relay Therapeutics, Roche, Sanofi, Takeda, Tempus AI, Thermo Fisher Scientific. Sabine Heublein reports advisory activity to MSD, Novartis, Eisai, Lilly, GSK; has received travel grants from Astra Zeneca, Novartis, GSK, Pfizer, Gilead; industry research funding by Novartis, SAGA; as well as honoraria by Roche, Pfizer, Astra Zeneca, GSK, Novartis, MSD, Clovis, Immunogen, Abbvie, Daichi Sankyo. Verena Thewes declares a travel grant from Gilead Sciences. Constantin Pixberg has received speakers fee from Merck Sharp and Dohme (MSD). Carlo Fremd received honoraria from Roche/Genentech, Pfizer, Astra Zeneca, GSK, Novartis, Veracyte, MSD, Eisai, Gilead, Lilly and research support from Roche/Genentech and Veracyte. Laura Michel received fees for speaking engagements and accommodation expenses in connection with congress participations from Roche, Pfizer, Lilly Pharma, Daiichi Sankyo, AstraZeneca, Gilead. Christian Maurer declares travel grants from Mundipharma, Amgen, Servier Deutschland GmbH, Abbvie, and consulting fees from Abbvie, Celgene/BMS and Pfizer. He received honoraria for lectures from Daiichi Sankyo, Novartis and Lilly. Stefan Fröhling reports consultancy fees from Illumina. Daniel Hübschmann reports stocks in Platomics GmbH. Katharina Smetanay received honoraria and travel grants from Daiichi Sankyo, Gilead, Lilly, MSD, Pfizer, Astra Zeneca. Richard F. Schlenk reports consulting fees/advisory boards: Abbvie, Daiichi Sankyo, Jazz, Pfizer Data Safety Monitoring boards: BerGen Bio, Novartis Steering Committee: Daiichi Sankyo Research funding: Abbvie, AstraZeneca, Boehringer Ingelheim, Daiichi Sankyo, PharmaMar, Pfizer, Roche, Recordati, Oncoheroes. Christoph Springfeld has served on Advisory boards at Astra Zeneca, Bayer, Incite, BMS, Revolution Medicines, Taiho and Servier. Albrecht Stenzinger reports advisory board/speaker's bureau for Agilent, Aignostics, Amgen, Astellas, Astra Zeneca, Bayer, BMS, Eli Lilly, Illumina, Incyte, Janssen, MSD, Novartis, Pfizer, Qlucore, QuiP, Roche, Sanofi, Seagene, Servier, Takeda, Thermo Fisher as well as grants by Bayer, BMS, MSD, Chugai, Incyte. Celina Wagner visited Astra Zeneca supported trainings. Dirk Jäger declares consulting fees by Roche Pharma AG, OncoOne Research & Development Research GmbH, Amgen Inc., CureVac AG; payments or honoraria for lectures, presentations, speaker's bureaus, manuscript writing or educational events by BMS GmbH & Co KGaA, MSD, Gruppe 4 Filmproduktion GmbH, The Norwegian Cancer Society, Dept. of Radiation Medicine, Univ. of Kentucky, Centrum für Integrierte Onkologie Bonn, Center for Modern Hospital Management Beijing; payment for expert testimony: Expert opinions for courts; support for attending meetings and/or travel by CureVac AG, Centrum für Integrierte Onkologie Bonn, BMS GmbH & Co KGaA, Berlin Institute of Health, Bioevents-congress.com., Oncogenics Inc. Parexel, FortTra GmbH BCPPC, Pancreas Center Karolinska Institute Sweden, AMGEN Inc. He participated on a Data Safety Monitoring Board or Advisory Boards from CureVac AG, Definiens, OncoOne Research & Development Research GmbH, Amgen Inc., Roche Pharma AG; leadership or fiduciary role in other boards, societies, committees, or advocacy groups paid or unpaid: BMS Stiftung Immunonkologie. All other authors declare no potential conflicts of interest.

## ETHICS STATEMENT

Enrolment was conducted under the ethics approval (by Institutional Review Board, IRB) of the CATCH study‐protocol (S‐164/2017; ClinicalTrials.gov Identifier: NCT05652569, https://clinicaltrials.gov/study/NCT05652569) at National Center for Tumor Diseases (NCT) Heidelberg, Germany. Patients' informed consent was collected from each individual taking part in the study.

## Supporting information


**FIGURE S1.** Details of patient cohort composition.
**FIGURE S2.** Implemented single agent and combined therapies.
**TABLE S1.** Statistics of therapy implementations across different outcome measures.
**TABLE S2.** Linking therapy response of PARP inhibitor treated patients to established and emerging predictive biomarkers.
**TABLE S3.** Sequencing coverage and quality statistics.

## Data Availability

Sequencing data are deposited at GHGA (The German Human Genome‐Phenome Archive) under accession number GHGAS52546615103987. Data will be available according to the established procedure of the Data Access Committee of Heidelberg Center for Personalized Oncology. Other data that support the findings of this study are available from the corresponding author upon reasonable request.
