# Supplementary material for: Delivering precision oncology in metastatic breast cancer: Clinical impact of comprehensive genomic profiling—The CATCH experience
Source: Int J Cancer. 2025 Oct 31;158(6):1675–89. doi: 10.1002/ijc.70208 (PMC12811195; doi:10.1002/ijc.70208)
Supplement: Supplementary file 1 — FIGURE S1. Details of patient cohort composition. FIGURE S2. Implemented single agent and combined therapies. TABLE S1. Statistics of therapy implementations across different outcome measures. TABLE S2. Linking therapy response of PARP inhibitor treated patients to established and emerging predictive biomarkers. TABLE S3. Sequencing coverage and quality statistics. [file IJC-158-1675-s001.pdf]

Supplementary Material for

Delivering precision oncology in metastatic breast cancer: Clinical impact of comprehensive genomic profiling – the CATCH experience

Mario Hlevnjak, Sabine Heublein, Verena Thewes, Lukas Wagener, Constantin Pixberg, Carlo Fremd, Laura Michel, Christian Maurer, Lars Buschhorn, Nicola Dikow, Fangyoumin Feng, Stefan Fröhling, Christel Herold-Mende, Steffen Hirsch, Chen Hong, Daniel Hübschmann, Lena Jassowicz, Polina Kozyulina, Katrin Pfütze, Richard F. Schlenk, Hans-Peter Sinn, Katharina Smetanay, Christoph Springfeld, Albrecht Stenzinger, Celina Wagner, Stephan Wolf, Andreas Trumpp, Dirk Jäger, Oliver Zivanovic, Marc Zapatka, Andreas Schneeweiss, Peter Lichter

Table of contents:

Supplementary Figure 1: Details of patient cohort composition.

Supplementary Figure 2: Implemented single agent and combined therapies.

Supplementary Table 1: Statistics of therapy implementations across different outcome measures

Supplementary Table 2: Linking therapy response of PARP inhibitor treated patients to established and emerging predictive biomarkers

Supplementary Table 3: Sequencing coverage and quality statistics

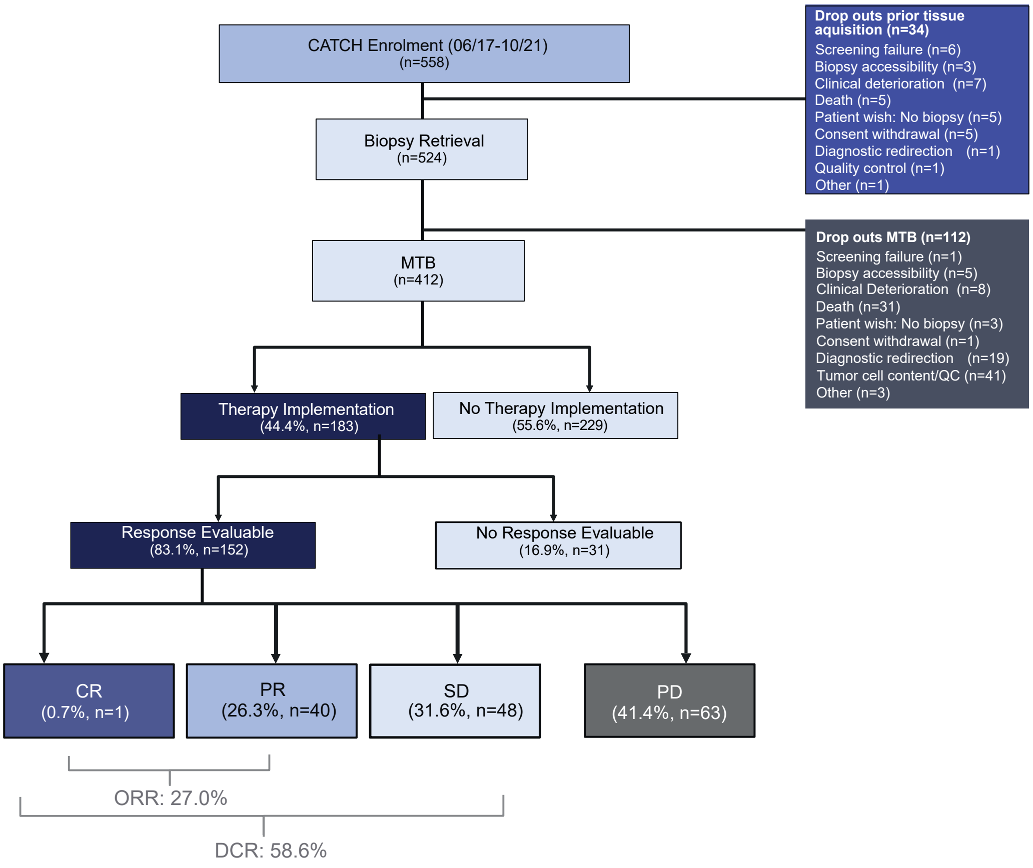

Supplementary Figure 1 Details of patient cohort composition.
Extended version of Figure 1, also including details of patient drop outs depicting patients, who did not undergo biopsy (n=34) and patients, who were not eligible for molecular tumor board analyses (n=112).

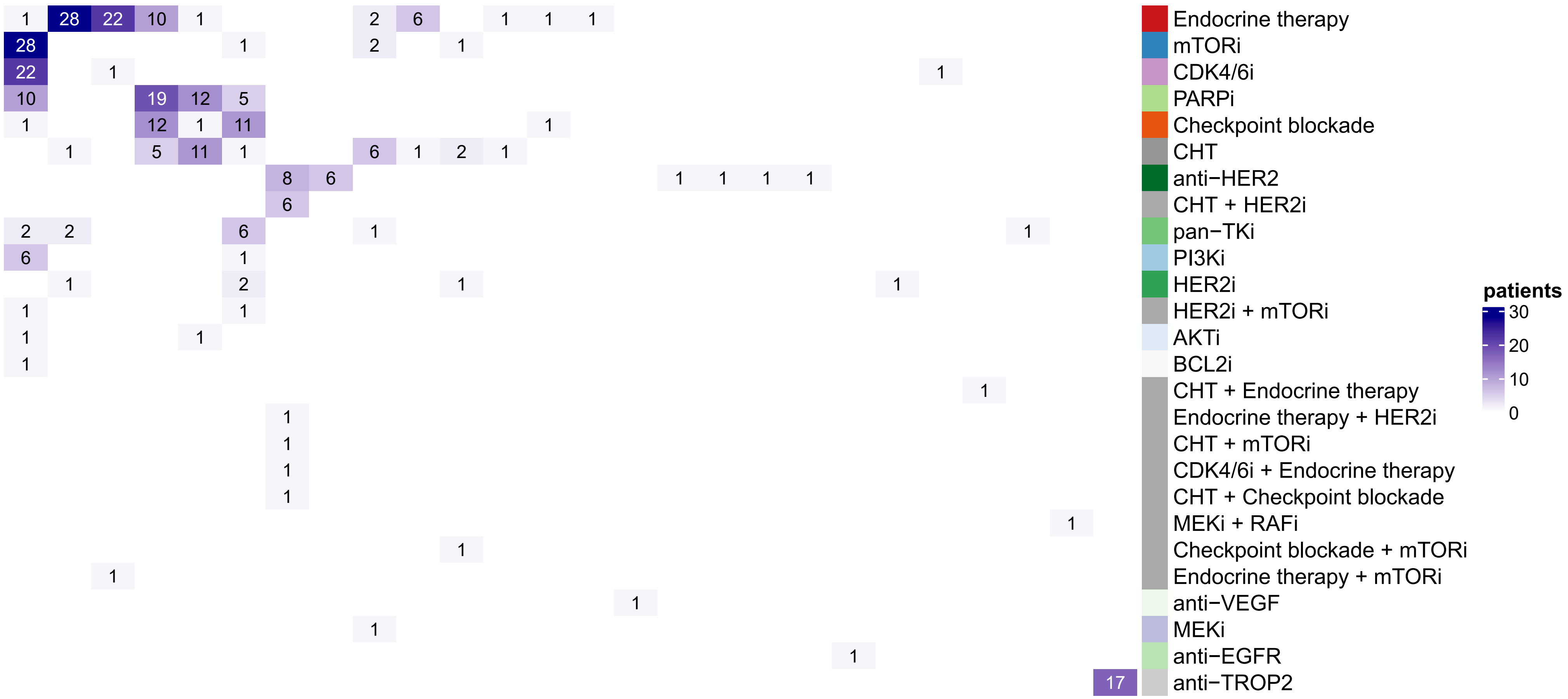

Supplementary Figure 2
Implemented single agent and combined therapies.

Extended version of Figure 3B showing heatmap with the detailed frequency distribution of monotherapies (n=52) and combination therapies (n=131) including doublet (n=115) and triplet combinations (n=16) regarding the different mechanisms of action

Supplementary Table 1. Targeted therapy implementations (see Figure 5): Statistics on difference in percentages between MTB-driven vs. clinical standard and off-label vs. in-label across different outcome measures (PFS2/PFS1≥1.5, ORR and DCR, 2-sample test for equality of proportions with continuity correction).

	%(MTB-driven - clinical standard)	95% CI	p-value	
PFS2/PFS1 ≥1.5	16.29%	[-0.57%, 33.15%]	0.070	
ORR	8.64%	[-7.9%, 25.16%]	0.365	
DCR	8.02%	[-10.52%, 26.57%]	0.454	
	%(off-label - in-label)	95% CI	p-value	
PFS2/PFS1 ≥1.5	19.21%	[2.45%, 35.97%]	0.030	*
ORR	11.47%	[-5.15%, 28.09%]	0.210	
DCR	20.68%	[2.33%, 39.03%]	0.028	*

Supplementary Table 2: Linking therapy response of PARP inhibitor treated patients to established and emerging predictive biomarkers

Patient group	PFS ratio ≥ 1.5<br><br>(evaluable n=34)	Objective response rate<br>(evaluable n=39)	Disease control rate<br>(evaluable n=39)
Patients with established biomarkers (germline or somatic BRCA1/2 and germline PALB2 inactivating alterations), n=22	6/18<br>33.3%	10/22<br>45.5%	17/22<br>77.3%
Patients with emerging biomarkers (excluding germline or somatic BRCA1/2 and PALB2 alterations), n=24	3/16<br>18.8%	2/17<br>11.8%	5/17<br>29.4%

Supplementary Table 3: Sequencing coverage and quality statistics
Whole Genome Sequencing

Sample ID	Total number of sequenced reads	Total number of uniquely mapped non duplicate reads	Total number of covered bases	Median coverage (and range) per base	Percentage of targeted bases with coverage >=10
**A26K-3F54SR_buffy-coat1-01**	1.96E+09	1.71E+09	2.28E+11	80 (0-641585)	90.396
**H021-3W9ZP5_blood**	2.03E+09	1.75E+09	2.27E+11	79 (0-330960)	90.4362
**H021-3W9ZP5_metastasis031-01**	2.01E+09	1.78E+09	2.37E+11	97 (0-386305)	90.3901
**H021-3W9ZP5_blood**	2.03E+09	1.75E+09	2.27E+11	79 (0-330960)	90.4362
**K26K-11UKEH_buffy-coat2**	1.96E+09	1.67E+09	2.24E+11	78 (0-166028)	90.3962
**K26K-11UKEH_tumor12**	1.93E+09	1.67E+09	2.22E+11	74 (0-175987)	90.4084
**K26K-13FTNS_buffy-coat2-01**	2.2E+09	1.93E+09	2.45E+11	86 (0-209582)	90.3842
**K26K-13FTNS_metastasis12-01**	2.15E+09	1.87E+09	2.47E+11	89 (0-560952)	90.3586
**K26K-19AFN7_buffy-coat2-01**	2.08E+09	1.78E+09	2.36E+11	82 (0-312381)	90.4129
**K26K-19AFN7_tumor13-01**	2.13E+09	1.88E+09	2.46E+11	82 (0-498757)	90.3034
**K26K-1ER5BG_buffy-coat2**	2.13E+09	1.81E+09	2.4E+11	84 (0-1115394)	90.3932
**K26K-1ER5BG_metastasis11**	2.14E+09	1.82E+09	2.44E+11	82 (0-276857)	90.396
**K26K-1F29VR_buffy-coat1-01-p**	2.79E+08	2.48E+08	2.23E+10	7 (0-45290)	28.6181
**K26K-1F29VR_metastasis13-01-p**	2.92E+08	2.47E+08	2.22E+10	6 (0-52701)	26.0761
**K26K-1GUTR3_buffy-coat2**	1.81E+09	1.65E+09	2.22E+11	77 (0-261593)	90.4439
**K26K-1GUTR3_metastasis11**	1.82E+09	1.66E+09	2.25E+11	74 (0-238805)	90.3581
**K26K-1K6GZP_buffy-coat1-01**	2.17E+09	1.83E+09	2.45E+11	85 (0-804251)	90.3309
**K26K-1K6GZP_metastasis11-01**	2.12E+09	1.84E+09	2.39E+11	82 (0-408067)	90.3589
**K26K-1MDU5E_buffy-coat2-01**	2.14E+09	1.82E+09	2.44E+11	85 (0-594573)	90.309
**K26K-1MDU5E_metastasis11-01**	2.1E+09	1.79E+09	2.39E+11	77 (0-469561)	90.2612
**K26K-1NV3L2_buffy-coat1-01**	2.04E+09	1.74E+09	2.33E+11	81 (0-340077)	90.3759
**K26K-1NV3L2_metastasis11-01**	2.04E+09	1.77E+09	2.39E+11	75 (0-290687)	90.2841
**K26K-1TZC25_buffy-coat1-01**	1.92E+09	1.67E+09	2.22E+11	77 (0-276243)	90.4266
**K26K-1TZC25_metastasis11-01**	2E+09	1.7E+09	2.27E+11	71 (0-253586)	90.3502
**K26K-1UGE3K_buffy-coat2-01**	2.1E+09	1.78E+09	2.38E+11	83 (0-805099)	90.3744
**K26K-1UGE3K_metastasis11-01**	1.99E+09	1.75E+09	2.32E+11	76 (0-232553)	90.3416
**K26K-1YV1NR_buffy-coat2**	2.11E+09	1.84E+09	2.46E+11	86 (0-319051)	90.4076
**K26K-1YV1NR_metastasis11**	2.09E+09	1.84E+09	2.44E+11	79 (0-287674)	90.393
**K26K-1YXDJQ_buffy-coat1**	2.2E+09	1.85E+09	2.48E+11	87 (0-452215)	90.4821
**K26K-1YXDJQ_metastasis11**	1.81E+09	1.61E+09	2.16E+11	75 (0-162253)	90.4074

**K26K-25C1GA_buffy-coat1-01**	2.06E+09	1.82E+09	2.44E+11	85 (0-647045)	90.3555
**K26K-25C1GA_metastasis11-01**	2.19E+09	1.93E+09	2.59E+11	88 (0-828010)	90.3733
**K26K-2ALFPG_buffy-coat2-01**	2.1E+09	1.72E+09	2.29E+11	80 (0-382072)	90.4442
**K26K-2ALFPG_tumor11-01**	2.07E+09	1.73E+09	2.25E+11	72 (0-353010)	90.4106
**K26K-2HQFG6_buffy-coat2**	1.93E+09	1.69E+09	2.32E+11	81 (0-150006)	90.436
**K26K-2HQFG6_metastasis11**	1.86E+09	1.67E+09	2.25E+11	76 (0-156709)	90.1244
**K26K-2LSYUD_buffy-coat1**	2.01E+09	1.79E+09	2.39E+11	84 (0-170161)	90.4542
**K26K-2LSYUD_tumor11**	1.89E+09	1.69E+09	2.25E+11	70 (0-431658)	90.2079
**K26K-2WDLYE_buffy-coat2**	2.09E+09	1.75E+09	2.31E+11	81 (0-1270577)	90.3494
**K26K-2WDLYE_tumor11**	2.12E+09	1.78E+09	2.37E+11	76 (0-406771)	90.319
**K26K-2X1DPV_buffy-coat1-01**	1.89E+09	1.67E+09	2.18E+11	76 (0-236832)	90.3309
**K26K-2X1DPV_metastasis11-01**	1.85E+09	1.63E+09	2.21E+11	76 (0-183196)	90.3003
**K26K-372K7Q_buffy-coat2**	2E+09	1.72E+09	2.28E+11	80 (0-843174)	90.3652
**K26K-372K7Q_metastasis11**	1.96E+09	1.71E+09	2.3E+11	71 (0-795241)	90.2326
**K26K-38J27P_buffy-coat1**	2.18E+09	1.92E+09	2.57E+11	90 (0-303862)	90.482
**K26K-38J27P_metastasis11**	2.11E+09	1.81E+09	2.42E+11	83 (0-317477)	90.4443
**K26K-38JWK8_buffy-coat1**	1.99E+09	1.74E+09	2.34E+11	82 (0-175959)	90.4466
**K26K-38JWK8_metastasis11**	2.05E+09	1.77E+09	2.35E+11	80 (0-173050)	90.4479
**K26K-3GWRN9_buffy-coat1-01**	1.94E+09	1.73E+09	2.29E+11	80 (0-201409)	90.3228
**K26K-3GWRN9_metastasis11-01**	1.95E+09	1.73E+09	2.25E+11	68 (0-219870)	90.2598
**K26K-3J6HU5_buffy-coat2**	2.04E+09	1.81E+09	2.47E+11	86 (0-244536)	90.4683
**K26K-3J6HU5_tumor11**	2.06E+09	1.79E+09	2.42E+11	84 (0-241431)	90.4558
**K26K-3SMV2Y_buffy-coat1-01**	2.06E+09	1.81E+09	2.47E+11	86 (0-403591)	90.3557
**K26K-3SMV2Y_metastasis11-01**	1.9E+09	1.69E+09	2.25E+11	79 (0-187382)	90.2614
**K26K-3SV5GJ_buffy-coat2**	2E+09	1.71E+09	2.28E+11	80 (0-1328406)	90.4099
**K26K-3SV5GJ_metastasis11**	1.96E+09	1.72E+09	2.3E+11	77 (0-159046)	90.4222
**K26K-3TN2B7_buffy-coat1-01**	1.88E+09	1.52E+09	2.04E+11	71 (0-351790)	90.383
**K26K-3TN2B7_tumor11-01**	1.85E+09	1.55E+09	2.06E+11	68 (0-231631)	90.3065
**K26K-3UL22G_buffy-coat1-01**	1.6E+09	1.43E+09	1.93E+11	66 (0-303853)	90.2849
**K26K-3UL22G_metastasis11-01**	2.07E+09	1.85E+09	2.49E+11	82 (0-476546)	90.2656
**K26K-3VGWL4_buffy-coat2-01**	2.13E+09	1.88E+09	2.46E+11	86 (0-464791)	90.4827
**K26K-3VGWL4_metastasis11-01**	2.16E+09	1.88E+09	2.45E+11	77 (0-464732)	90.3252
**K26K-3Y6UFG_buffy-coat1**	1.97E+09	1.73E+09	2.28E+11	80 (0-239059)	90.4383
**K26K-3Y6UFG_metastasis11**	2.01E+09	1.77E+09	2.3E+11	77 (0-248297)	90.3834
**K26K-3Z85N5_buffy-coat1-01**	2.41E+09	2.19E+09	2.92E+11	102 (0-423069)	90.4792
**K26K-3Z85N5_tumor11-01**	2.39E+09	2.16E+09	2.86E+11	98 (0-451972)	90.4671
**K26K-43JZGQ_buffy-coat2**	1.82E+09	1.56E+09	2.08E+11	72 (0-503966)	90.3425
**K26K-43JZGQ_tumor12**	1.69E+09	1.51E+09	2.01E+11	67 (0-152498)	90.3214
**K26K-49T9ZE_buffy-coat2**	2E+09	1.71E+09	2.27E+11	79 (0-216855)	90.3524

**K26K-49T9ZE_metastasis11**	1.86E+09	1.63E+09	2.16E+11	66 (0-191206)	90.2408
**K26K-4AUP7Y_buffy-coat1**	2.04E+09	1.75E+09	2.35E+11	82 (0-272249)	90.3477
**K26K-4AUP7Y_tumor11**	2.08E+09	1.85E+09	2.46E+11	79 (0-229204)	90.3197
**K26K-4JNSU8_buffy-coat1-01**	1.96E+09	1.7E+09	2.29E+11	80 (0-294314)	90.3414
**K26K-4JNSU8_metastasis11-01**	1.93E+09	1.67E+09	2.2E+11	78 (0-308842)	90.1675
**K26K-4L12DA_buffy-coat1-01**	2.13E+09	1.82E+09	2.43E+11	85 (0-531695)	90.3526
**K26K-4L12DA_metastasis11-01**	2.18E+09	1.85E+09	2.42E+11	77 (0-2735667)	90.329
**K26K-4MUH3K_buffy-coat2-01**	2.19E+09	1.82E+09	2.34E+11	82 (0-390826)	90.3434
**K26K-4MUH3K_metastasis12-01**	2.06E+09	1.75E+09	2.28E+11	72 (0-392003)	90.3036
**K26K-4PEUSL_buffy-coat1**	1.85E+09	1.69E+09	2.28E+11	80 (0-195162)	90.387
**K26K-4PEUSL_metastasis11**	1.87E+09	1.69E+09	2.26E+11	71 (0-162989)	90.2819
**K26K-4RJ5UK_buffy-coat1**	1.83E+09	1.61E+09	2.17E+11	75 (0-190408)	90.2488
**K26K-4RJ5UK_metastasis12**	1.81E+09	1.61E+09	2.13E+11	69 (0-197096)	90.2594
**K26K-4UEVHA_buffy-coat1-01**	2.07E+09	1.77E+09	2.37E+11	83 (0-313893)	90.3188
**K26K-4UEVHA_tumor12-01**	2.09E+09	1.81E+09	2.46E+11	76 (0-285341)	90.2487
**K26K-4V9MRS_buffy-coat2-01**	2.11E+09	1.84E+09	2.28E+11	80 (0-443259)	90.4502
**K26K-4V9MRS_metastasis11-01**	2.11E+09	1.87E+09	2.37E+11	77 (0-473439)	90.4189
**K26K-4VP5ZM_buffy-coat1-01**	2.1E+09	1.86E+09	2.49E+11	86 (0-438481)	90.4055
**K26K-4VP5ZM_metastasis22-01**	2.23E+09	2E+09	2.68E+11	83 (0-455537)	90.3702
**K26K-52RKM3_buffy-coat1-01**	2.1E+09	1.84E+09	2.48E+11	87 (0-666853)	90.357
**K26K-52RKM3_metastasis11-01**	2.09E+09	1.82E+09	2.44E+11	77 (0-233327)	90.1943
**K26K-53KLXE_buffy-coat2**	2.15E+09	1.86E+09	2.46E+11	86 (0-1352099)	90.4679
**K26K-53KLXE_metastasis12**	2.17E+09	1.89E+09	2.54E+11	86 (0-234435)	90.4805
**K26K-54UFHM_buffy-coat1**	1.99E+09	1.74E+09	2.27E+11	79 (0-224395)	90.4562
**K26K-54UFHM_metastasis12**	1.99E+09	1.72E+09	2.24E+11	77 (0-205938)	90.4496
**K26K-595J2V_buffy-coat1-01**	2.26E+09	2.03E+09	2.74E+11	95 (0-741481)	90.3465
**K26K-595J2V_metastasis12-01**	2.17E+09	1.96E+09	2.63E+11	91 (0-574282)	90.3303
**K26K-59LEYZ_buffy-coat1**	1.82E+09	1.66E+09	2.12E+11	74 (0-208096)	90.3664
**K26K-59LEYZ_metastasis11**	1.89E+09	1.69E+09	2.2E+11	70 (0-145508)	90.3242
**K26K-5AJQY4_buffy-coat1-01**	2.18E+09	1.99E+09	2.67E+11	93 (0-708306)	90.3377
**K26K-5AJQY4_metastasis11-01**	1.87E+09	1.71E+09	2.29E+11	76 (0-523124)	90.2535
**K26K-5AYB4Y_buffy-coat1-01**	2.04E+09	1.81E+09	2.38E+11	84 (0-194878)	90.955
**K26K-5AYB4Y_metastasis11-01**	1.99E+09	1.76E+09	2.33E+11	77 (0-572307)	90.888
**K26K-5ELXUS_buffy-coat1-01**	2.18E+09	1.85E+09	2.41E+11	84 (0-381951)	90.3734
**K26K-5ELXUS_metastasis11-01**	2.14E+09	1.85E+09	2.48E+11	83 (0-832135)	90.388
**K26K-5JM4B6_buffy-coat2**	2.09E+09	1.74E+09	2.34E+11	82 (0-328514)	90.3649
**K26K-5JM4B6_metastasis12**	1.96E+09	1.72E+09	2.29E+11	76 (0-420177)	90.1585
**K26K-5M96VM_buffy-coat1-01-p**	2.85E+08	2.57E+08	2.3E+10	7 (0-47168)	31.3961

**K26K-5M96VM_metastasis13-01-p**	3.53E+08	2.77E+08	2.44E+10	7 (0-58885)	32.5263
**K26K-5NNPD2_buffy-coat1**	1.75E+09	1.58E+09	2.12E+11	74 (0-117608)	90.4007
**K26K-5NNPD2_metastasis12**	1.79E+09	1.59E+09	2.13E+11	68 (0-173563)	90.3574
**K26K-5QQNGF_buffy-coat1-01**	2.1E+09	1.91E+09	2.56E+11	89 (0-472649)	90.3749
**K26K-5QQNGF_metastasis22-01**	2.08E+09	1.9E+09	2.55E+11	81 (0-401856)	90.3471
**K26K-5S8AT2_buffy-coat1-01**	2.09E+09	1.83E+09	2.38E+11	83 (0-307222)	90.4442
**K26K-5S8AT2_metastasis11-01**	2.07E+09	1.8E+09	2.38E+11	82 (0-290601)	90.4476
**K26K-5XRAND_buffy-coat2-01**	2.17E+09	1.86E+09	2.45E+11	86 (0-460592)	90.387
**K26K-5XRAND_metastasis11-01**	2.15E+09	1.84E+09	2.46E+11	85 (0-466377)	90.4002
**K26K-62B38Y_buffy-coat1-01**	1.86E+09	1.69E+09	2.27E+11	79 (0-659504)	90.379
**K26K-62B38Y_metastasis12-01**	2.01E+09	1.81E+09	2.45E+11	84 (0-398558)	90.2563
**K26K-65NG9T_buffy-coat1-01**	2.22E+09	1.99E+09	2.68E+11	93 (0-590942)	90.3857
**K26K-65NG9T_tumor11-01**	1.72E+09	1.57E+09	2.12E+11	69 (0-341413)	90.2878
**K26K-6AR69Z_buffy-coat2-01**	1.91E+09	1.7E+09	2.21E+11	77 (0-397311)	90.4466
**K26K-6AR69Z_metastasis12-01**	1.92E+09	1.7E+09	2.21E+11	75 (0-412332)	90.431
**K26K-6DAZ5U_buffy-coat2**	2.09E+09	1.79E+09	2.39E+11	83 (0-244572)	90.3989
**K26K-6DAZ5U_metastasis12**	2.03E+09	1.74E+09	2.31E+11	75 (0-207944)	90.298
**K26K-6FAWPW_buffy-coat1**	2.23E+09	1.82E+09	2.34E+11	82 (0-375020)	90.4834
**K26K-6FAWPW_metastasis11**	2.18E+09	1.79E+09	2.38E+11	79 (0-264644)	90.446
**K26K-6FX6FM_buffy-coat1-02**	3.12E+09	2.77E+09	3.72E+11	129 (0-1114523)	90.4705
**K26K-6FX6FM_metastasis11-01**	2.34E+09	2.12E+09	2.85E+11	96 (0-959158)	90.3703
**K26K-6GJXKU_buffy-coat2**	2.1E+09	1.78E+09	2.4E+11	84 (0-290787)	90.4472
**K26K-6GJXKU_metastasis11**	2.14E+09	1.75E+09	2.35E+11	81 (0-231652)	90.4345
**K26K-6LFEUV_buffy-coat1-01**	1.97E+09	1.72E+09	2.33E+11	81 (0-248156)	90.4487
**K26K-6LFEUV_tumor12-01**	1.8E+09	1.58E+09	2.14E+11	69 (0-193363)	90.3707
**K26K-6PJ115_buffy-coat1**	1.91E+09	1.69E+09	2.31E+11	80 (0-120334)	90.4301
**K26K-6PJ115_metastasis11**	1.81E+09	1.63E+09	2.15E+11	75 (0-123963)	90.4169
**K26K-6PKB4F_buffy-coat2-01**	1.97E+09	1.72E+09	2.32E+11	81 (0-329974)	90.4112
**K26K-6PKB4F_metastasis11-01**	2.03E+09	1.77E+09	2.39E+11	78 (0-305046)	90.3852
**K26K-6QT98R_buffy-coat1-01**	1.98E+09	1.75E+09	2.33E+11	81 (0-289154)	90.4103
**K26K-6QT98R_metastasis11-01**	1.87E+09	1.65E+09	2.2E+11	75 (0-244268)	90.392
**K26K-6S8M2L_buffy-coat2-01**	2E+09	1.68E+09	2.22E+11	78 (0-274403)	90.3639
**K26K-6S8M2L_tumor11-01**	2.01E+09	1.71E+09	2.28E+11	81 (0-687043)	90.3549
**K26K-6SJWVZ_buffy-coat1-01**	2.56E+09	2.29E+09	3.08E+11	107 (0-588918)	90.3935
**K26K-6SJWVZ_tumor11-01**	2.09E+09	1.89E+09	2.54E+11	80 (0-534712)	90.3302
**K26K-6SKXE7_buffy-coat2-01**	2.07E+09	1.79E+09	2.37E+11	83 (0-238606)	90.3884
**K26K-6SKXE7_metastasis11-01**	2.06E+09	1.79E+09	2.39E+11	78 (0-318883)	90.3354
**K26K-6T2U2T_buffy-coat1-01**	2.02E+09	1.77E+09	2.34E+11	81 (0-299738)	90.325
**K26K-6T2U2T_metastasis11-01**	2.12E+09	1.88E+09	2.52E+11	83 (0-439201)	90.3458

**K26K-6TUAKJ_buffy-coat2**	2.04E+09	1.7E+09	2.29E+11	80 (0-215007)	90.3436
**K26K-6TUAKJ_tumor11**	2.02E+09	1.75E+09	2.3E+11	79 (0-279396)	90.2434
**K26K-6Z62PR_buffy-coat2-01**	2.02E+09	1.77E+09	2.22E+11	78 (0-397356)	90.3886
**K26K-6Z62PR_metastasis11-01**	1.88E+09	1.67E+09	2.18E+11	69 (0-307243)	90.375
**K26K-79C7RG_buffy-coat2-01**	1.97E+09	1.71E+09	2.28E+11	80 (0-308499)	90.3825
**K26K-79C7RG_metastasis11-01**	1.88E+09	1.63E+09	2.14E+11	64 (0-218028)	90.2813
**K26K-7ARCJQ_buffy-coat1-01**	2.08E+09	1.73E+09	2.33E+11	82 (0-347657)	90.8919
**K26K-7ARCJQ_metastasis12-01**	2.04E+09	1.74E+09	2.34E+11	78 (0-302400)	90.881
**K26K-7BQYTL_buffy-coat1-01**	1.62E+09	1.51E+09	2E+11	70 (0-462554)	90.3228
**K26K-7BQYTL_metastasis11-01**	1.89E+09	1.66E+09	2.23E+11	67 (0-130133)	90.2627
**K26K-7FVDMP_buffy-coat1-01**	1.97E+09	1.71E+09	2.32E+11	81 (0-388292)	90.3098
**K26K-7FVDMP_metastasis11-01**	2.01E+09	1.7E+09	2.23E+11	73 (0-1618024)	90.2739
**K26K-7GHTVB_buffy-coat1-01**	2E+09	1.73E+09	2.33E+11	81 (0-165027)	90.3797
**K26K-7GHTVB_metastasis11-01**	2.04E+09	1.75E+09	2.36E+11	78 (0-175330)	90.3303
**K26K-7K2ESR_buffy-coat2**	2.01E+09	1.73E+09	2.29E+11	80 (0-308231)	90.3544
**K26K-7K2ESR_metastasis11**	1.94E+09	1.71E+09	2.2E+11	76 (0-289910)	90.3529
**K26K-7Q6MXJ_buffy-coat2-01**	2.18E+09	1.89E+09	2.52E+11	88 (0-199438)	90.3816
**K26K-7Q6MXJ_tumor11-01**	2.03E+09	1.78E+09	2.31E+11	75 (0-1279064)	90.3233
**K26K-7TXP85_buffy-coat2-01**	2.12E+09	1.87E+09	2.48E+11	87 (0-243768)	90.3964
**K26K-7TXP85_metastasis12-01**	2.1E+09	1.78E+09	2.37E+11	79 (0-294869)	90.3688
**K26K-7UH38T_buffy-coat2**	2.08E+09	1.84E+09	2.44E+11	85 (0-232136)	90.3915
**K26K-7UH38T_tumor11**	2.07E+09	1.83E+09	2.34E+11	80 (0-223813)	90.3659
**K26K-7VJE74_buffy-coat1-01**	2.77E+09	2.39E+09	3.24E+11	113 (0-901442)	90.3915
**K26K-7VJE74_metastasis11-01**	2.53E+09	2.28E+09	3.03E+11	93 (0-744334)	90.2572
**K26K-82VGF5_buffy-coat1-01**	2.44E+09	2.23E+09	2.97E+11	105 (0-888711)	89.8988
**K26K-82VGF5_metastasis12-01**	2.77E+09	2.49E+09	3.34E+11	104 (0-740653)	89.6581
**K26K-89CAXB_buffy-coat2**	1.97E+09	1.7E+09	2.28E+11	80 (0-205986)	90.3945
**K26K-89CAXB_tumor11**	2.03E+09	1.74E+09	2.32E+11	76 (0-444905)	90.3507
**K26K-8S2LMY_buffy-coat1-01-p**	2.59E+08	2.32E+08	2.07E+10	6 (0-53064)	24.3318
**K26K-8S2LMY_metastasis13-01-p**	2.72E+08	2.24E+08	1.96E+10	6 (0-61025)	19.5525
**K26K-8SNWXL_buffy-coat2**	1.9E+09	1.69E+09	2.23E+11	78 (0-440754)	90.3358
**K26K-8SNWXL_metastasis11**	1.93E+09	1.71E+09	2.25E+11	72 (0-430007)	90.3149
**K26K-8VYLK7_buffy-coat1-01-p**	3.82E+08	3.5E+08	3.14E+10	10 (0-48839)	55.3786
**K26K-8VYLK7_metastasis11-01-p**	2.88E+08	1.8E+08	1.56E+10	4 (0-78128)	12.914
**K26K-92LDBN_buffy-coat2**	1.79E+09	1.64E+09	2.24E+11	78 (0-239002)	90.4324
**K26K-92LDBN_metastasis11**	1.82E+09	1.63E+09	2.19E+11	75 (0-216241)	90.391

**K26K-96DFP9_buffy-coat1**	1.89E+09	1.68E+09	2.22E+11	77 (0-202281)	90.297
**K26K-96DFP9_tumor11**	1.99E+09	1.68E+09	2.25E+11	78 (0-441507)	90.3003
**K26K-9EASMF_buffy-coat1-01**	2.16E+09	1.92E+09	2.53E+11	88 (0-541270)	90.4644
**K26K-9EASMF_metastasis11-01**	2.03E+09	1.77E+09	2.37E+11	79 (0-476456)	90.3974
**K26K-9G8979_buffy-coat2**	2.01E+09	1.7E+09	2.31E+11	81 (0-173263)	90.4416
**K26K-9G8979_metastasis11**	2.03E+09	1.69E+09	2.29E+11	73 (0-174782)	90.3626
**K26K-9KDVFY_buffy-coat1-01**	2.38E+09	2.13E+09	2.86E+11	100 (0-775000)	90.3595
**K26K-9KDVFY_metastasis11-01**	1.99E+09	1.83E+09	2.46E+11	76 (0-624299)	90.2983
**K26K-9LGPEA_buffy-coat1-01**	2.03E+09	1.77E+09	2.34E+11	81 (0-337335)	90.3413
**K26K-9LGPEA_metastasis21-01**	1.98E+09	1.74E+09	2.34E+11	81 (0-272738)	90.3125
**K26K-9NVTNQ_buffy-coat2**	2.03E+09	1.72E+09	2.31E+11	81 (0-390282)	90.3339
**K26K-9NVTNQ_metastasis22**	2.18E+09	1.85E+09	2.46E+11	82 (0-1174972)	90.353
**K26K-A1FE3E_buffy-coat2**	1.99E+09	1.76E+09	2.33E+11	81 (0-464683)	90.3206
**K26K-A1FE3E_metastasis11**	2.05E+09	1.82E+09	2.44E+11	80 (0-444839)	90.2644
**K26K-A3YEMT_buffy-coat1-01**	2.08E+09	1.79E+09	2.4E+11	84 (0-341311)	90.333
**K26K-A3YEMT_metastasis12-01**	2.05E+09	1.76E+09	2.32E+11	78 (0-3216441)	90.3071
**K26K-A96VU1_buffy-coat2-01**	2.19E+09	1.9E+09	2.5E+11	87 (0-505079)	90.3658
**K26K-A96VU1_metastasis12-01**	2.2E+09	1.92E+09	2.58E+11	80 (0-434036)	90.2853
**K26K-AEC16Q_buffy-coat1-01**	2.56E+09	2.27E+09	3.06E+11	107 (0-569143)	90.3648
**K26K-AEC16Q_metastasis12-01**	2.03E+09	1.83E+09	2.45E+11	84 (0-494731)	90.3027
**K26K-AHDG8P_buffy-coat1-01**	1.99E+09	1.7E+09	2.25E+11	79 (0-488602)	90.3997
**K26K-AHDG8P_metastasis11-01**	1.92E+09	1.68E+09	2.18E+11	71 (0-343710)	90.3442
**K26K-ALSG3K_buffy-coat2-01**	1.89E+09	1.73E+09	2.3E+11	81 (0-560341)	89.7876
**K26K-ALSG3K_metastasis21-01**	2.33E+09	2.15E+09	2.86E+11	99 (0-831481)	89.8779
**K26K-AN7W62_buffy-coat2**	2.13E+09	1.79E+09	2.33E+11	81 (0-395337)	90.3568
**K26K-AN7W62_metastasis11**	2.13E+09	1.83E+09	2.43E+11	85 (0-460249)	90.2971
**K26K-ARB5RQ_buffy-coat1-01**	1.92E+09	1.72E+09	2.32E+11	80 (0-449176)	90.3292
**K26K-ARB5RQ_metastasis11-01**	1.64E+09	1.48E+09	1.98E+11	65 (0-721159)	90.2476
**K26K-AT59U7_buffy-coat1-01-p**	3.09E+08	2.73E+08	2.46E+10	8 (0-48677)	35.5354
**K26K-AT59U7_metastasis13-01-p**	2.47E+08	2.07E+08	1.76E+10	5 (0-49377)	15.6129
**K26K-AVQZ6J_buffy-coat1-01**	2.01E+09	1.72E+09	2.3E+11	80 (0-270977)	90.3356
**K26K-AVQZ6J_tumor11-01**	1.96E+09	1.67E+09	2.24E+11	70 (0-247590)	90.2159
**K26K-B21ADR_buffy-coat1-01-p**	3.25E+08	2.89E+08	2.59E+10	8 (0-47437)	40.2717
**K26K-B21ADR_tumor11-01-p**	2.56E+08	2.18E+08	1.93E+10	5 (0-53679)	21.4851
**K26K-B3P4PS_buffy-coat2**	1.95E+09	1.65E+09	2.22E+11	78 (0-165002)	90.3985
**K26K-B3P4PS_tumor12**	2.03E+09	1.71E+09	2.29E+11	76 (0-176647)	90.3945
**K26K-B5D94J_buffy-coat1-01-p**	2.57E+08	2.27E+08	2.03E+10	6 (0-42732)	23.0332

**K26K-B5D94J_metastasis11-01-p**	4.54E+08	3.73E+08	3.31E+10	10 (0-56128)	54.8805
**K26K-B5VPJQ_buffy-coat1-01**	2.36E+09	2.1E+09	2.82E+11	98 (0-736886)	90.3781
**K26K-B5VPJQ_metastasis11-01**	2.34E+09	2.07E+09	2.77E+11	95 (0-641180)	90.3646
**K26K-B8MUBJ_buffy-coat1**	1.81E+09	1.63E+09	2.16E+11	76 (0-170177)	90.3851
**K26K-B8MUBJ_metastasis12**	1.78E+09	1.6E+09	2.12E+11	66 (0-149758)	90.3485
**K26K-BF6J54_buffy-coat1-01-p**	2.21E+08	1.89E+08	1.7E+10	5 (0-40003)	14.5674
**K26K-BF6J54_tumor12-01-p**	2.76E+08	2.25E+08	1.94E+10	5 (0-50070)	17.2389
**K26K-BHSR57_buffy-coat2-01**	2.12E+09	1.87E+09	2.51E+11	88 (0-917508)	90.3756
**K26K-BHSR57_metastasis11-01**	2.09E+09	1.87E+09	2.49E+11	83 (0-432954)	90.3568
**K26K-BJVSF7_buffy-coat2-01**	2.04E+09	1.72E+09	2.27E+11	79 (0-272561)	90.3809
**K26K-BJVSF7_metastasis11-01**	2.06E+09	1.75E+09	2.32E+11	80 (0-263970)	90.346
**K26K-BQAVRQ_buffy-coat2**	1.92E+09	1.68E+09	2.25E+11	79 (0-280331)	90.4036
**K26K-BQAVRQ_metastasis12**	1.92E+09	1.68E+09	2.19E+11	75 (0-258444)	90.3945
**K26K-BRET3U_buffy-coat1**	1.78E+09	1.61E+09	2.13E+11	74 (0-179515)	90.3187
**K26K-BRET3U_metastasis11**	1.83E+09	1.62E+09	2.16E+11	68 (0-181082)	90.2392
**K26K-BUNZZM_buffy-coat2-01**	2.12E+09	1.82E+09	2.43E+11	85 (0-256283)	90.3835
**K26K-BUNZZM_metastasis11-01**	2.11E+09	1.79E+09	2.42E+11	80 (0-289626)	90.3353
**K26K-BY9U8C_buffy-coat1-01**	2.05E+09	1.68E+09	2.25E+11	79 (0-304072)	90.4176
**K26K-BY9U8C_metastasis11-01**	2.1E+09	1.72E+09	2.31E+11	79 (0-318597)	90.3901
**K26K-BZC91B_buffy-coat1-01**	2.17E+09	1.84E+09	2.48E+11	86 (0-831994)	90.4054
**K26K-BZC91B_metastasis11-01**	2.18E+09	1.87E+09	2.53E+11	83 (0-324377)	90.3899
**K26K-C1DQWG_buffy-coat1-01**	3.07E+09	2.67E+09	3.6E+11	126 (0-919718)	90.449
**K26K-C1DQWG_tumor12-01**	2.29E+09	2.02E+09	2.74E+11	88 (0-965185)	90.329
**K26K-C34LTG_buffy-coat1-01-p**	1.25E+08	1.16E+08	1.04E+10	3 (0-34684)	2.04207
**K26K-C34LTG_metastasis12-01-p**	2.17E+08	2E+08	1.79E+10	6 (0-40215)	15.8301
**K26K-CAG4DA_buffy-coat2**	2.03E+09	1.8E+09	2.4E+11	84 (0-306827)	90.4845
**K26K-CAG4DA_tumor11**	1.98E+09	1.75E+09	2.32E+11	78 (0-303172)	90.455
**K26K-CEZTWE_buffy-coat1-01**	2.05E+09	1.89E+09	2.53E+11	88 (0-859723)	90.3126
**K26K-CEZTWE_metastasis12-01**	1.9E+09	1.75E+09	2.33E+11	78 (0-697651)	90.2681
**K26K-CHHDF2_buffy-coat1**	2.09E+09	1.85E+09	2.49E+11	87 (0-575909)	90.3672
**K26K-CHHDF2_metastasis11**	2.1E+09	1.85E+09	2.5E+11	80 (0-426242)	90.2487
**K26K-CK5DRG_buffy-coat2-01**	2.16E+09	1.85E+09	2.5E+11	87 (0-392106)	90.3776
**K26K-CK5DRG_metastasis11-01**	2.07E+09	1.76E+09	2.35E+11	76 (0-352307)	90.316
**K26K-CRA69B_buffy-coat1-01-p**	2.52E+08	2.29E+08	2.06E+10	7 (0-47587)	22.9392
**K26K-CRA69B_metastasis13-01-p**	3.76E+08	2.75E+08	2.34E+10	7 (0-71261)	28.8097
**K26K-CTD3KC_buffy-coat1-01**	2.11E+09	1.88E+09	2.53E+11	88 (0-568633)	90.3406
**K26K-CTD3KC_metastasis11-01**	1.9E+09	1.72E+09	2.32E+11	72 (0-484786)	90.2655
**K26K-CVQCDD_buffy-coat1-01-p**	1.24E+08	1.12E+08	1E+10	3 (0-36693)	2.12945

**K26K-CVQCDD_metastasis11-01-p**	1.63E+08	1.41E+08	1.27E+10	3 (0-45610)	10.9182
**K26K-CYERP8_buffy-coat1-01**	2.47E+09	2.2E+09	2.96E+11	103 (0-503189)	90.3658
**K26K-CYERP8_tumor11-01**	2.46E+09	2.22E+09	2.98E+11	96 (0-579190)	90.3349
**K26K-D63727_buffy-coat2**	1.97E+09	1.75E+09	2.31E+11	81 (0-355141)	90.0615
**K26K-D63727_tumor11**	1.92E+09	1.68E+09	2.24E+11	74 (0-437367)	90.3005
**K26K-D7A6KB_buffy-coat2-01**	2.19E+09	1.84E+09	2.28E+11	80 (0-492589)	90.3242
**K26K-D7A6KB_metastasis11-01**	2.12E+09	1.78E+09	2.32E+11	79 (0-456656)	90.2578
**K26K-DA2KDN_buffy-coat1-01**	2.33E+09	2.04E+09	2.75E+11	96 (0-636256)	90.3444
**K26K-DA2KDN_metastasis12-01**	2.54E+09	2.32E+09	3.03E+11	91 (0-1030227)	90.3156
**K26K-DDJVUJ_buffy-coat2**	1.99E+09	1.72E+09	2.34E+11	82 (0-172579)	90.3761
**K26K-DDJVUJ_metastasis12**	2.08E+09	1.78E+09	2.42E+11	78 (0-171732)	89.4002
**K26K-DLDYB3_buffy-coat1-01**	2.16E+09	1.86E+09	2.53E+11	86 (0-300117)	80.2786
**K26K-DLDYB3_tumor11-01**	2.05E+09	1.78E+09	2.4E+11	80 (0-257739)	90.3415
**K26K-DMU6TK_buffy-coat2**	2.13E+09	1.85E+09	2.47E+11	81 (0-275746)	69.76
**K26K-DMU6TK_tumor11**	2.17E+09	1.82E+09	2.39E+11	77 (0-312635)	90.2934
**K26K-DX72G5_buffy-coat2-01**	2.08E+09	1.79E+09	2.43E+11	85 (0-510297)	90.3366
**K26K-DX72G5_metastasis11-01**	2.08E+09	1.78E+09	2.4E+11	80 (0-400802)	77.9379
**K26K-E59Y51_buffy-coat2-01**	2.01E+09	1.73E+09	2.3E+11	76 (0-455541)	70.6766
**K26K-E59Y51_metastasis11-01**	1.86E+09	1.65E+09	2.17E+11	76 (0-434346)	90.293
**K26K-E6Q28L_buffy-coat1-01**	2.11E+09	1.81E+09	2.42E+11	85 (0-343649)	90.3438
**K26K-E6Q28L_tumor11-01**	2.14E+09	1.86E+09	2.47E+11	86 (0-1286908)	90.3376
**K26K-E6QE8X_buffy-coat1-01**	2.18E+09	1.85E+09	2.47E+11	81 (0-433416)	68.2812
**K26K-E6QE8X_metastasis11-01**	2.1E+09	1.8E+09	2.39E+11	80 (0-346529)	90.3547
**K26K-E84U1B_buffy-coat1-01**	2.06E+09	1.81E+09	2.42E+11	84 (0-233177)	90.3834
**K26K-E84U1B_metastasis12-01**	2.12E+09	1.84E+09	2.39E+11	73 (0-198707)	90.2205
**K26K-EJL1KW_buffy-coat2-01**	2.04E+09	1.74E+09	2.35E+11	82 (0-293915)	90.3326
**K26K-EJL1KW_metastasis11-01**	1.85E+09	1.63E+09	2.15E+11	74 (0-216125)	90.285
**K26K-EK5C3C_buffy-coat2-01**	2E+09	1.77E+09	2.36E+11	82 (0-350109)	90.4482
**K26K-EK5C3C_metastasis12-01**	1.97E+09	1.74E+09	2.31E+11	78 (0-306895)	90.4118
**K26K-ELSFBE_buffy-coat2-01**	2.24E+09	1.88E+09	2.42E+11	84 (0-594550)	90.3598
**K26K-ELSFBE_metastasis11-01**	2.21E+09	1.83E+09	2.41E+11	78 (0-574264)	90.2594
**K26K-EPTTB9_buffy-coat2-01**	2.13E+09	1.83E+09	2.46E+11	86 (0-399694)	90.3427
**K26K-EPTTB9_metastasis11-01**	2.24E+09	1.96E+09	2.57E+11	90 (0-1582394)	90.3532
**K26K-EQ9XTQ_buffy-coat2-01**	2.09E+09	1.75E+09	2.29E+11	80 (0-397154)	90.4284
**K26K-EQ9XTQ_metastasis13-01**	2.12E+09	1.83E+09	2.32E+11	76 (0-487992)	90.4
**K26K-ES3KU3_buffy-coat1-01**	2.16E+09	1.9E+09	2.5E+11	87 (0-374285)	90.4313
**K26K-ES3KU3_tumor11-01**	2.12E+09	1.84E+09	2.45E+11	79 (0-357954)	90.3816

**K26K-ETBARK_buffy-coat1-02**	2.6E+09	2.31E+09	3.1E+11	108 (0-638453)	90.4677
**K26K-ETBARK_tumor11-01**	2.62E+09	2.38E+09	3.2E+11	104 (0-859878)	90.4367
**K26K-EUWQV4_buffy-coat1**	1.89E+09	1.69E+09	2.27E+11	79 (0-172529)	90.4043
**K26K-EUWQV4_tumor11**	1.82E+09	1.63E+09	2.18E+11	74 (0-93114)	90.291
**K26K-EVQJ85_buffy-coat1**	2.05E+09	1.76E+09	2.38E+11	83 (0-202756)	90.3215
**K26K-EVQJ85_tumor11**	2.04E+09	1.78E+09	2.37E+11	77 (0-740269)	90.2851
**K26K-EVVHM1_buffy-coat1**	1.94E+09	1.68E+09	2.17E+11	77 (0-573771)	90.2584
**K26K-EVVHM1_metastasis12**	2.12E+09	1.69E+09	2.21E+11	79 (0-700752)	90.258
**K26K-F4BWZA_buffy-coat2**	1.91E+09	1.68E+09	2.22E+11	78 (0-759140)	90.3587
**K26K-F4BWZA_tumor11**	1.91E+09	1.66E+09	2.25E+11	74 (0-551816)	90.0883
**K26K-F5V95D_buffy-coat2**	1.97E+09	1.71E+09	2.26E+11	79 (0-479686)	90.3285
**K26K-F5V95D_metastasis11**	1.98E+09	1.71E+09	2.31E+11	77 (0-448167)	90.2941
**K26K-F61AM4_buffy-coat2-01**	2.11E+09	1.81E+09	2.45E+11	85 (0-263953)	90.3565
**K26K-F61AM4_metastasis12-01**	2.1E+09	1.81E+09	2.43E+11	84 (0-267493)	90.3453
**K26K-F7Z1YC_buffy-coat1**	2.06E+09	1.8E+09	2.27E+11	79 (0-1082779)	90.3491
**K26K-F7Z1YC_metastasis12**	2.05E+09	1.77E+09	2.31E+11	72 (0-566066)	90.2995
**K26K-FAN1MB_buffy-coat1-01**	2.08E+09	1.87E+09	2.52E+11	87 (0-445208)	90.3621
**K26K-FAN1MB_metastasis11-01**	2.12E+09	1.87E+09	2.51E+11	85 (0-348112)	90.3513
**K26K-FCKMTM_buffy-coat1-01**	1.94E+09	1.75E+09	2.36E+11	82 (0-571080)	90.3239
**K26K-FCKMTM_metastasis12-01**	1.89E+09	1.69E+09	2.27E+11	75 (0-665365)	90.2683
**K26K-FDGLB6_buffy-coat1-01**	2E+09	1.83E+09	2.47E+11	86 (0-535371)	90.318
**K26K-FDGLB6_tumor12-01**	2.14E+09	1.96E+09	2.64E+11	92 (0-612623)	90.3197
**K26K-FDYVED_buffy-coat2-01**	2.12E+09	1.83E+09	2.42E+11	84 (0-172268)	90.369
**K26K-FDYVED_metastasis12-01**	2.1E+09	1.8E+09	2.39E+11	83 (0-177069)	90.3821
**K26K-FHFEVH_buffy-coat2-01-p**	3.01E+08	2.58E+08	2.28E+10	7 (0-52318)	31.5118
**K26K-FHFEVH_tumor11-01-p**	3.08E+08	2.22E+08	1.95E+10	5 (0-96640)	23.074
**K26K-FQZN9K_buffy-coat1-01**	2.48E+09	2.16E+09	2.9E+11	101 (0-720209)	90.3904
**K26K-FQZN9K_metastasis11-01**	2.11E+09	1.86E+09	2.5E+11	78 (0-575581)	90.2811
**K26K-FXNF6U_buffy-coat1-01**	2.05E+09	1.79E+09	2.4E+11	84 (0-287740)	90.4463
**K26K-FXNF6U_metastasis11-01**	2.03E+09	1.79E+09	2.4E+11	80 (0-295219)	90.4157
**K26K-FZ3PWP_buffy-coat1**	2.05E+09	1.77E+09	2.31E+11	81 (0-562644)	90.3585
**K26K-FZ3PWP_metastasis11**	2.03E+09	1.79E+09	2.32E+11	74 (0-2086540)	90.3232
**K26K-G1K1C8_buffy-coat1**	1.83E+09	1.64E+09	2.18E+11	76 (0-215956)	90.3133
**K26K-G1K1C8_metastasis11**	1.91E+09	1.7E+09	2.26E+11	75 (0-216543)	90.3139
**K26K-G4S7NK_buffy-coat1**	1.96E+09	1.7E+09	2.33E+11	81 (0-143370)	90.4438
**K26K-G4S7NK_metastasis12**	1.9E+09	1.67E+09	2.25E+11	71 (0-147694)	90.3808
**K26K-G7Q3UN_buffy-coat1-01**	2.01E+09	1.82E+09	2.43E+11	84 (0-734075)	90.3543

**K26K-G7Q3UN_tumor11-01**	2.07E+09	1.87E+09	2.51E+11	84 (0-675783)	90.3201
**K26K-G7RGRH_buffy-coat1**	1.92E+09	1.72E+09	2.32E+11	81 (0-172016)	90.4655
**K26K-G7RGRH_metastasis11**	1.9E+09	1.69E+09	2.29E+11	68 (0-133733)	90.3198
**K26K-G8PLW1_buffy-coat1-01**	3.2E+09	2.81E+09	3.74E+11	131 (0-999523)	90.4023
**K26K-G8PLW1_metastasis12-01**	3.49E+09	3.04E+09	4.12E+11	143 (0-1027624)	90.4333
**K26K-GBZY7E_buffy-coat1-01**	2.01E+09	1.72E+09	2.32E+11	81 (0-248443)	90.4238
**K26K-GBZY7E_metastasis12-01**	1.91E+09	1.65E+09	2.23E+11	72 (0-212264)	90.3561
**K26K-GE6LK5_buffy-coat1-01**	1.97E+09	1.62E+09	2.18E+11	76 (0-241298)	90.3896
**K26K-GE6LK5_tumor12-01**	1.81E+09	1.53E+09	2.05E+11	66 (0-193258)	90.3267
**K26K-GEBVMR_buffy-coat1-01**	1.99E+09	1.75E+09	2.31E+11	81 (0-602189)	90.3468
**K26K-GEBVMR_metastasis12-01**	1.97E+09	1.74E+09	2.33E+11	79 (0-313882)	90.3193
**K26K-GG93LJ_buffy-coat2-01**	2.06E+09	1.78E+09	2.38E+11	83 (0-751396)	90.3376
**K26K-GG93LJ_metastasis22-01**	2.01E+09	1.77E+09	2.36E+11	78 (0-236066)	90.2384
**K26K-GGT9WV_buffy-coat1**	2.06E+09	1.68E+09	2.24E+11	78 (0-495217)	90.3576
**K26K-GGT9WV_metastasis11**	2.07E+09	1.82E+09	2.32E+11	77 (0-1012778)	90.2788
**K26K-GP1DEZ_buffy-coat2**	2.05E+09	1.7E+09	2.3E+11	80 (0-177185)	90.3494
**K26K-GP1DEZ_metastasis12**	2.1E+09	1.75E+09	2.35E+11	73 (0-193881)	90.2917
**K26K-GQ6XGU_buffy-coat2**	1.92E+09	1.7E+09	2.3E+11	80 (0-422938)	90.339
**K26K-GQ6XGU_metastasis22**	1.94E+09	1.69E+09	2.28E+11	77 (0-425448)	90.3321
**K26K-GR7U4J_buffy-coat1**	2.14E+09	1.88E+09	2.51E+11	88 (0-604319)	90.3824
**K26K-GR7U4J_metastasis11**	2.17E+09	1.86E+09	2.5E+11	85 (0-570398)	90.3528
**K26K-GRKG3V_buffy-coat2-01**	2.07E+09	1.82E+09	2.45E+11	86 (0-491855)	90.3175
**K26K-GRKG3V_metastasis11-01**	2.07E+09	1.84E+09	2.43E+11	77 (0-396712)	90.2153
**K26K-H63HRT_buffy-coat2**	1.76E+09	1.58E+09	2.09E+11	73 (0-191992)	90.4117
**K26K-H63HRT_tumor11**	1.8E+09	1.61E+09	2.15E+11	71 (0-189478)	90.3991
**K26K-H9FT8X_buffy-coat1-01**	2.09E+09	1.81E+09	2.39E+11	83 (0-427018)	90.4386
**K26K-H9FT8X_metastasis11-01**	2.12E+09	1.86E+09	2.46E+11	83 (0-423102)	90.3837
**K26K-HANJG1_buffy-coat1-01**	2.44E+09	2.21E+09	2.98E+11	104 (0-529187)	90.3908
**K26K-HANJG1_metastasis11-01**	2.5E+09	2.22E+09	2.98E+11	97 (0-677684)	90.2226
**K26K-HGTZ3B_buffy-coat3-01**	1.59E+09	1.43E+09	1.92E+11	67 (0-324352)	90.2489
**K26K-HGTZ3B_metastasis11-01**	1.53E+09	1.4E+09	1.86E+11	64 (0-318210)	90.225
**K26K-HGX6HZ_metastasis11-01**	1.88E+09	1.64E+09	2.21E+11	76 (0-352449)	90.3307
**K26K-HGXQVM_buffy-coat2**	2.24E+09	1.91E+09	2.37E+11	83 (0-284149)	90.3397
**K26K-HGXQVM_tumor11**	2.1E+09	1.75E+09	2.3E+11	79 (0-236832)	90.345
**K26K-HH4Q3M_buffy-coat1**	1.94E+09	1.75E+09	2.17E+11	76 (0-238475)	90.2958
**K26K-HH4Q3M_tumor12**	2.02E+09	1.75E+09	2.34E+11	79 (0-280438)	90.3044
**K26K-HJDPFX_buffy-coat1-01**	2.06E+09	1.81E+09	2.43E+11	85 (0-360736)	90.3601
**K26K-HJDPFX_metastasis11-01**	2.04E+09	1.79E+09	2.41E+11	82 (0-373836)	90.3512

**K26K-HKH6GS_buffy-coat1**	1.79E+09	1.62E+09	2.17E+11	76 (0-153441)	90.3912
**K26K-HKH6GS_metastasis11**	1.84E+09	1.67E+09	2.21E+11	68 (0-153752)	90.3011
**K26K-HL95W4_buffy-coat1-01**	2.07E+09	1.83E+09	2.41E+11	84 (0-238641)	90.4755
**K26K-HL95W4_metastasis11-01**	2.07E+09	1.85E+09	2.43E+11	81 (0-234585)	90.4666
**K26K-HNE5A5_buffy-coat1-01**	2.19E+09	1.96E+09	2.63E+11	92 (0-584015)	90.3973
**K26K-HNE5A5_metastasis11-01**	2.6E+09	2.31E+09	3.09E+11	101 (0-752899)	90.4115
**K26K-HPGSLN_buffy-coat1-01**	2.06E+09	1.84E+09	2.46E+11	86 (0-357977)	90.3386
**K26K-HPGSLN_metastasis11-01**	2.28E+09	2.03E+09	2.72E+11	91 (0-563483)	90.3276
**K26K-HQ88ZT_buffy-coat1-01**	2.06E+09	1.84E+09	2.49E+11	86 (0-537484)	90.3558
**K26K-HQ88ZT_metastasis11-01**	1.79E+09	1.61E+09	2.17E+11	72 (0-721160)	90.3062
**K26K-HRURBQ_buffy-coat1-01**	2.04E+09	1.78E+09	2.4E+11	84 (0-310077)	90.4431
**K26K-HRURBQ_buffy-coat1-02**	2.12E+09	1.93E+09	2.58E+11	90 (0-561598)	90.3664
**K26K-HRURBQ_metastasis11-01**	1.96E+09	1.69E+09	2.26E+11	76 (0-271314)	90.427
**K26K-HTWSX6_buffy-coat1-01**	2.13E+09	1.81E+09	2.4E+11	84 (0-379081)	90.4339
**K26K-HTWSX6_metastasis11-01**	2.08E+09	1.75E+09	2.34E+11	79 (0-344748)	90.2928
**K26K-HUGYN6_buffy-coat1-01**	2.3E+09	2.1E+09	2.81E+11	97 (0-830181)	90.3936
**K26K-HUGYN6_tumor11-01**	2.78E+09	2.44E+09	3.25E+11	112 (0-920490)	90.4186
**K26K-HV34DF_buffy-coat1-01**	2.41E+09	2.18E+09	2.91E+11	101 (0-811711)	90.3609
**K26K-HV34DF_metastasis12-01**	2.06E+09	1.88E+09	2.52E+11	83 (0-633326)	90.2525
**K26K-J1JGYX_buffy-coat1-01-p2**	1.2E+08	1.04E+08	9.26E+09	3 (0-43395)	1.53043
**K26K-J1JGYX_metastasis1-01-p2**	3.14E+08	2.49E+08	2.21E+10	7 (0-49164)	29.4512
**K26K-J54JQM_buffy-coat1-01**	1.86E+09	1.65E+09	2.22E+11	78 (0-321835)	90.4193
**K26K-J54JQM_metastasis11-01**	1.97E+09	1.71E+09	2.26E+11	72 (0-305975)	90.3114
**K26K-J8FA7A_buffy-coat1-01**	1.97E+09	1.77E+09	2.38E+11	83 (0-281713)	90.4441
**K26K-J8FA7A_metastasis11-01**	1.98E+09	1.78E+09	2.39E+11	74 (0-264616)	90.3588
**K26K-JCDPJQ_buffy-coat1-01**	2.11E+09	1.83E+09	2.41E+11	84 (0-1217545)	90.3733
**K26K-JCDPJQ_metastasis11-01**	2.08E+09	1.79E+09	2.42E+11	74 (0-321736)	90.3171
**K26K-JCDSWD_buffy-coat1-01**	1.87E+09	1.66E+09	2.22E+11	77 (0-258466)	90.4244
**K26K-JCDSWD_tumor11-01**	2.01E+09	1.74E+09	2.3E+11	77 (0-261862)	90.4228
**K26K-JEQKXE_buffy-coat2-01**	2.63E+09	2.36E+09	3.17E+11	110 (0-791769)	90.4328
**K26K-JEQKXE_metastasis31-01**	2.28E+09	2.05E+09	2.75E+11	93 (0-699704)	90.3942
**K26K-JF2LWZ_buffy-coat2**	1.84E+09	1.64E+09	2.2E+11	76 (0-183380)	90.4368
**K26K-JF2LWZ_metastasis11**	1.83E+09	1.54E+09	2.06E+11	71 (0-148488)	90.315
**K26K-JG8K8P_buffy-coat2**	1.73E+09	1.55E+09	2.09E+11	73 (0-169125)	90.4108
**K26K-JG8K8P_metastasis11**	1.83E+09	1.65E+09	2.22E+11	73 (0-168625)	90.4212
**K26K-JHFXVG_buffy-coat2-01-p**	2.73E+08	2.38E+08	2.14E+10	7 (0-42795)	25.4524
**K26K-JHFXVG_metastasis13-01-p**	2.91E+08	1.94E+08	1.67E+10	5 (0-61036)	14.7698

**K26K-JJERC1_buffy-coat2-01**	1.96E+09	1.75E+09	2.28E+11	79 (0-438642)	90.2941
**K26K-JJERC1_tumor11-01**	1.9E+09	1.7E+09	2.27E+11	77 (0-367583)	90.3037
**K26K-JPH1C6_buffy-coat1-01**	1.97E+09	1.69E+09	2.28E+11	80 (0-471598)	90.323
**K26K-JPH1C6_metastasis11-01**	1.89E+09	1.61E+09	2.2E+11	72 (0-568069)	90.2892
**K26K-JS49GN_buffy-coat1-01**	2.1E+09	1.7E+09	2.28E+11	80 (0-610621)	90.3218
**K26K-JS49GN_metastasis11-01**	2.08E+09	1.75E+09	2.38E+11	76 (0-128061)	90.3047
**K26K-JSP6N7_buffy-coat2-01-p**	1.05E+08	93184414	8.38E+09	2 (0-38388)	0.876661
**K26K-JSP6N7_metastasis11-01-p**	2.25E+08	1.67E+08	1.37E+10	4 (0-58809)	9.24641
**K26K-JTXSFQ_buffy-coat1-01**	2.09E+09	1.8E+09	2.4E+11	84 (0-240897)	90.3416
**K26K-JTXSFQ_tumor12-01**	2.03E+09	1.75E+09	2.36E+11	80 (0-236545)	90.3102
**K26K-JYXU3U_buffy-coat1-01**	2.04E+09	1.87E+09	2.51E+11	87 (0-122987)	90.414
**K26K-JYXU3U_metastasis12-01**	2.1E+09	1.87E+09	2.49E+11	86 (0-131323)	90.3832
**K26K-KAN58L_buffy-coat2**	2.17E+09	1.86E+09	2.54E+11	89 (0-205160)	90.4694
**K26K-KAN58L_metastasis11**	2.18E+09	1.85E+09	2.5E+11	85 (0-184918)	90.4348
**K26K-KBMSKG_buffy-coat1**	1.91E+09	1.66E+09	2.18E+11	76 (0-298648)	90.2767
**K26K-KBMSKG_tumor12**	1.89E+09	1.71E+09	2.18E+11	69 (0-264849)	90.1937
**K26K-KDGJQR_buffy-coat2**	2.08E+09	1.73E+09	2.32E+11	81 (0-262316)	90.4879
**K26K-KDGJQR_metastasis11**	2.07E+09	1.74E+09	2.34E+11	79 (0-265494)	90.4885
**K26K-KGYV17_buffy-coat1-01**	1.96E+09	1.78E+09	2.4E+11	83 (0-469187)	90.3691
**K26K-KGYV17_metastasis11-01**	2.44E+09	1.96E+09	2.62E+11	85 (0-370007)	90.3633
**K26K-KM1V83_buffy-coat2-01**	2.15E+09	1.91E+09	2.48E+11	87 (0-276603)	90.428
**K26K-KM1V83_metastasis11-01**	2.13E+09	1.87E+09	2.46E+11	83 (0-303142)	90.3974
**K26K-KN2BCS_buffy-coat1-01-p**	2.67E+08	2.35E+08	2.11E+10	7 (0-49890)	24.6141
**K26K-KN2BCS_metastasis13-01-p**	3.3E+08	2.78E+08	2.41E+10	7 (0-62704)	31.5834
**K26K-KQYMSV_buffy-coat2**	1.86E+09	1.67E+09	2.25E+11	79 (0-194086)	90.4173
**K26K-KQYMSV_metastasis12**	1.83E+09	1.63E+09	2.13E+11	71 (0-182520)	90.403
**K26K-KS8MHJ_buffy-coat1-01**	2.07E+09	1.81E+09	2.43E+11	85 (0-216764)	90.3614
**K26K-KS8MHJ_metastasis11-01**	1.98E+09	1.78E+09	2.42E+11	79 (0-166716)	90.3308
**K26K-KUNL2W_buffy-coat1-01**	2.92E+09	2.61E+09	3.51E+11	122 (0-627056)	90.4269
**K26K-KUNL2W_metastasis11-01**	2.39E+09	2.18E+09	2.91E+11	98 (0-788317)	90.3721
**K26K-KV9VSG_buffy-coat2-01**	2.18E+09	1.9E+09	2.56E+11	90 (0-471150)	90.3558
**K26K-KV9VSG_tumor11-01**	2.2E+09	1.91E+09	2.51E+11	85 (0-404195)	90.3419
**K26K-KX19MG_buffy-coat1-01**	1.77E+09	1.62E+09	2.16E+11	75 (0-536576)	90.3412
**K26K-KX19MG_tumor12-01**	2.16E+09	1.95E+09	2.6E+11	87 (0-677035)	90.3781
**K26K-KYLKAW_buffy-coat1-01**	1.98E+09	1.78E+09	2.39E+11	83 (0-468306)	90.3561
**K26K-KYLKAW_metastasis12-01**	2.16E+09	1.96E+09	2.62E+11	91 (0-536747)	90.3826
**K26K-L31YS4_buffy-coat2-01**	2.15E+09	1.84E+09	2.46E+11	86 (0-355103)	90.4253
**K26K-L31YS4_metastasis11-01**	2.15E+09	1.81E+09	2.4E+11	82 (0-338510)	90.401

**K26K-L3MWGQ_buffy-coat2-01-p**	3E+08	2.62E+08	2.34E+10	7 (0-46671)	32.5375
**K26K-L3MWGQ_metastasis11-01-p**	2.6E+08	2.04E+08	1.62E+10	4 (0-58166)	13.3356
**K26K-L5XH9B_buffy-coat2**	1.83E+09	1.63E+09	2.19E+11	76 (0-232452)	90.4095
**K26K-L5XH9B_metastasis11**	1.86E+09	1.66E+09	2.23E+11	75 (0-218967)	90.4146
**K26K-L5Z7JE_buffy-coat1-01**	2.1E+09	1.81E+09	2.42E+11	84 (0-315881)	90.4442
**K26K-L5Z7JE_tumor11-01**	2.12E+09	1.82E+09	2.44E+11	75 (0-327575)	90.3659
**K26K-LA3ZXV_buffy-coat1-01-p**	2.64E+08	2.36E+08	2.13E+10	7 (0-46284)	25.0171
**K26K-LA3ZXV_tumor13-01-p**	2.37E+08	2.12E+08	1.89E+10	6 (0-45084)	17.8339
**K26K-LDBKHW_buffy-coat2-01**	2.24E+09	1.86E+09	2.39E+11	84 (0-5376993)	90.3276
**K26K-LDBKHW_tumor11-01**	2.23E+09	1.91E+09	2.44E+11	85 (0-542481)	90.2879
**K26K-LEVSEC_buffy-coat2-01**	2.14E+09	1.9E+09	2.49E+11	87 (0-534238)	90.3851
**K26K-LEVSEC_metastasis11-01**	2.1E+09	1.87E+09	2.45E+11	81 (0-456548)	90.3249
**K26K-LG21QX_buffy-coat2-01**	2.12E+09	1.83E+09	2.36E+11	82 (0-360191)	90.3335
**K26K-LG21QX_metastasis11-01**	2.04E+09	1.77E+09	2.32E+11	77 (0-656983)	90.3635
**K26K-LHW5P8_buffy-coat2**	1.91E+09	1.72E+09	2.28E+11	80 (0-396200)	90.3655
**K26K-LHW5P8_metastasis12**	1.9E+09	1.68E+09	2.23E+11	76 (0-385019)	90.3571
**K26K-LKHUJ5_buffy-coat1-01**	1.76E+09	1.6E+09	2.16E+11	75 (0-540583)	90.3022
**K26K-LKHUJ5_tumor11-01**	2.07E+09	1.85E+09	2.49E+11	84 (0-471185)	90.3341
**K26K-LKKMR2_buffy-coat2**	1.84E+09	1.63E+09	2.18E+11	76 (0-520977)	90.3416
**K26K-LKKMR2_metastasis11**	1.85E+09	1.66E+09	2.22E+11	74 (0-486025)	90.3287
**K26K-LKP6WZ_buffy-coat1-01**	2.11E+09	1.82E+09	2.43E+11	85 (0-358267)	90.442
**K26K-LKP6WZ_metastasis12-01**	2.14E+09	1.85E+09	2.41E+11	82 (0-385404)	90.4116
**K26K-LKZDBN_buffy-coat1-01**	2.22E+09	2E+09	2.68E+11	93 (0-566411)	90.3662
**K26K-LKZDBN_metastasis11-01**	2.21E+09	1.99E+09	2.65E+11	85 (0-535453)	90.3143
**K26K-LPBX5B_buffy-coat2-01**	2.13E+09	1.71E+09	2.29E+11	80 (0-123172)	90.356
**K26K-LPBX5B_metastasis12-01**	2.12E+09	1.74E+09	2.33E+11	73 (0-133360)	90.3329
**K26K-LSCN6M_buffy-coat1-01**	2.09E+09	1.82E+09	2.43E+11	85 (0-457773)	90.3594
**K26K-LSCN6M_metastasis11-01**	2.06E+09	1.77E+09	2.39E+11	70 (0-355930)	90.2462
**K26K-LUMBC5_buffy-coat2**	1.97E+09	1.7E+09	2.32E+11	81 (0-206371)	90.4377
**K26K-LUMBC5_metastasis11**	1.95E+09	1.7E+09	2.3E+11	78 (0-187261)	90.4396
**K26K-LW7WC8_buffy-coat1-01**	1.93E+09	1.7E+09	2.3E+11	80 (0-289028)	90.4234
**K26K-LW7WC8_metastasis13-01**	1.95E+09	1.73E+09	2.32E+11	80 (0-296084)	90.4372
**K26K-LXFJ6Q_buffy-coat2-01**	2.02E+09	1.77E+09	2.31E+11	81 (0-480988)	90.3348
**K26K-LXFJ6Q_tumor12-01**	1.83E+09	1.62E+09	2.14E+11	74 (0-529719)	90.3243
**K26K-LYG3BY_buffy-coat1-01**	2.26E+09	2.05E+09	2.76E+11	96 (0-462878)	90.3403
**K26K-LYG3BY_tumor11-01**	2.21E+09	1.99E+09	2.67E+11	89 (0-660027)	90.3207
**K26K-M61EFR_buffy-coat1-01**	2.07E+09	1.77E+09	2.39E+11	83 (0-275300)	90.4444
**K26K-M61EFR_metastasis11-01**	2.07E+09	1.8E+09	2.44E+11	84 (0-260107)	90.3774
**K26K-MAEFZL_buffy-coat2-01**	2.01E+09	1.78E+09	2.4E+11	84 (0-295016)	90.4278

**K26K-MAEFZL_metastasis11-01**	1.86E+09	1.66E+09	2.23E+11	72 (0-337072)	90.3917
**K26K-MF6NDW_buffy-coat2-01**	1.95E+09	1.75E+09	2.35E+11	82 (0-340135)	90.4321
**K26K-MF6NDW_tumor12-01**	1.86E+09	1.65E+09	2.17E+11	75 (0-441733)	90.375
**K26K-MGTYZH_buffy-coat2-01**	2.11E+09	1.81E+09	2.45E+11	85 (0-388161)	90.3341
**K26K-MGTYZH_metastasis12-01**	2.15E+09	1.83E+09	2.46E+11	83 (0-393532)	90.3099
**K26K-MK6UTZ_buffy-coat2-01**	2.11E+09	1.79E+09	2.41E+11	84 (0-250286)	90.3742
**K26K-MK6UTZ_metastasis11-01**	2.03E+09	1.72E+09	2.3E+11	78 (0-213777)	90.3552
**K26K-MLPAW4_buffy-coat2-01-p**	1.27E+08	1.07E+08	9.48E+09	3 (0-40120)	2.49219
**K26K-MLPAW4_tumor11-01-p**	1.4E+08	1.09E+08	9.57E+09	2 (0-48842)	6.90392
**K26K-MMXGSX_buffy-coat1-01-p**	2.97E+08	2.52E+08	2.27E+10	7 (0-50146)	29.5356
**K26K-MMXGSX_metastasis13-01-p**	2.35E+08	2.02E+08	1.79E+10	5 (0-50064)	15.686
**K26K-MNVK2W_buffy-coat2**	2.12E+09	1.85E+09	2.5E+11	87 (0-543037)	90.4178
**K26K-MNVK2W_metastasis11**	1.91E+09	1.67E+09	2.26E+11	75 (0-434517)	90.2926
**K26K-MXSQTG_buffy-coat2-01**	2.06E+09	1.83E+09	2.44E+11	85 (0-389606)	90.3609
**K26K-MXSQTG_tumor11-01**	1.89E+09	1.69E+09	2.28E+11	77 (0-381627)	90.3053
**K26K-MYECCH_buffy-coat1-01**	1.94E+09	1.72E+09	2.26E+11	79 (0-297374)	90.3756
**K26K-MYECCH_metastasis11-01**	2.15E+09	1.82E+09	2.35E+11	79 (0-278506)	90.373
**K26K-N2D9XV_buffy-coat2**	2E+09	1.79E+09	2.31E+11	81 (0-330156)	90.4173
**K26K-N2D9XV_metastasis11**	2.1E+09	1.78E+09	2.4E+11	82 (0-297953)	90.3462
**K26K-N3VNSA_buffy-coat2-01**	2.07E+09	1.81E+09	2.44E+11	85 (0-379360)	90.4139
**K26K-N3VNSA_metastasis11-01**	2.12E+09	1.86E+09	2.41E+11	83 (0-407929)	90.4165
**K26K-N422KN_buffy-coat2**	2.02E+09	1.73E+09	2.32E+11	81 (0-202315)	90.3385
**K26K-N422KN_metastasis11**	2.09E+09	1.79E+09	2.41E+11	83 (0-230058)	90.309
**K26K-N9SB5N_buffy-coat1-01**	1.85E+09	1.64E+09	2.2E+11	77 (0-246702)	90.4232
**K26K-N9SB5N_tumor11-01**	1.89E+09	1.69E+09	2.28E+11	72 (0-231852)	90.3657
**K26K-NBST9B_buffy-coat1-01**	2.05E+09	1.78E+09	2.39E+11	83 (0-259170)	90.3305
**K26K-NBST9B_metastasis12-01**	1.93E+09	1.7E+09	2.25E+11	69 (0-947777)	90.2682
**K26K-NDDF92_buffy-coat1**	2E+09	1.75E+09	2.33E+11	81 (0-595414)	90.3995
**K26K-NDDF92_metastasis11**	2.05E+09	1.78E+09	2.38E+11	76 (0-542737)	90.3567
**K26K-NEG3MQ_buffy-coat1-01**	1.99E+09	1.79E+09	2.4E+11	83 (0-602088)	90.3469
**K26K-NEG3MQ_metastasis12-01**	2.22E+09	1.99E+09	2.67E+11	86 (0-638321)	90.2968
**K26K-NKAGPR_buffy-coat1**	1.87E+09	1.65E+09	2.23E+11	77 (0-230382)	90.2731
**K26K-NKAGPR_metastasis11**	1.88E+09	1.66E+09	2.16E+11	71 (0-258175)	90.3024
**K26K-NNVS4U_buffy-coat1-01**	2.03E+09	1.72E+09	2.33E+11	81 (0-286392)	90.2856
**K26K-NNVS4U_tumor12-01**	1.97E+09	1.69E+09	2.25E+11	75 (0-234505)	90.3038
**K26K-NPC5CB_metastasis11-01**	2.45E+09	2.19E+09	2.96E+11	92 (0-569125)	90.2723
**K26K-NQH6T5_buffy-coat2-01**	2.09E+09	1.79E+09	2.43E+11	85 (0-402957)	90.3102
**K26K-NQH6T5_metastasis21-01**	2.08E+09	1.74E+09	2.35E+11	73 (0-424531)	90.2251

**K26K-NUUDDJ_buffy-coat2-01**	2.11E+09	1.87E+09	2.52E+11	88 (0-220911)	90.339
**K26K-NUUDDJ_tumor32-01**	2.12E+09	1.85E+09	2.49E+11	82 (0-230153)	90.3353
**K26K-NV29AR_buffy-coat1-01**	1.98E+09	1.76E+09	2.36E+11	82 (0-222591)	90.4452
**K26K-NV29AR_metastasis12-01**	2.09E+09	1.83E+09	2.43E+11	78 (0-902151)	90.3485
**K26K-NYMP4W_buffy-coat1-01**	2.14E+09	1.86E+09	2.35E+11	82 (0-1564962)	90.3835
**K26K-NYMP4W_metastasis12-01**	2.09E+09	1.86E+09	2.46E+11	79 (0-331169)	90.2785
**K26K-P6W7FU_buffy-coat2-01**	1.79E+09	1.59E+09	2.1E+11	73 (0-242217)	90.3375
**K26K-P6W7FU_metastasis11-01**	2.71E+09	2.31E+09	2.98E+11	97 (0-386305)	90.3901
**K26K-P76N2B_buffy-coat2-01**	1.93E+09	1.72E+09	2.27E+11	79 (0-284107)	90.3173
**K26K-P76N2B_metastasis12-01**	2.16E+09	1.88E+09	2.48E+11	73 (0-367096)	90.2255
**K26K-P7V4JK_buffy-coat1-01**	2.01E+09	1.76E+09	2.38E+11	83 (0-272245)	90.3531
**K26K-P7V4JK_tumor11-01**	2.04E+09	1.75E+09	2.36E+11	72 (0-236891)	90.2888
**K26K-P8EEDV_buffy-coat1**	1.93E+09	1.73E+09	2.23E+11	78 (0-150674)	90.3833
**K26K-P8EEDV_tumor11**	1.89E+09	1.71E+09	2.19E+11	75 (0-186485)	90.374
**K26K-PAZNZP_buffy-coat2**	2.07E+09	1.82E+09	2.33E+11	81 (0-262520)	90.3715
**K26K-PAZNZP_metastasis11**	2.15E+09	1.9E+09	2.38E+11	79 (0-2820648)	90.3087
**K26K-PCA7BN_buffy-coat2**	1.86E+09	1.66E+09	2.19E+11	76 (0-230801)	90.435
**K26K-PCA7BN_metastasis12**	1.89E+09	1.69E+09	2.25E+11	72 (0-166145)	90.4049
**K26K-PDJUSD_buffy-coat2**	1.79E+09	1.63E+09	2.16E+11	75 (0-407363)	90.4464
**K26K-PDJUSD_metastasis11**	1.82E+09	1.63E+09	2.15E+11	68 (0-178751)	90.4119
**K26K-PGV6SL_buffy-coat2-01**	2.11E+09	1.78E+09	2.33E+11	81 (0-533244)	90.3028
**K26K-PGV6SL_metastasis11-01**	2.14E+09	1.79E+09	2.43E+11	78 (0-495120)	90.2846
**K26K-PJV57M_buffy-coat1-01**	2.24E+09	2.06E+09	2.76E+11	96 (0-781601)	90.3702
**K26K-PJV57M_metastasis11-01**	2.07E+09	1.9E+09	2.55E+11	82 (0-571508)	90.3075
**K26K-PN66E8_buffy-coat1-01**	2.08E+09	1.78E+09	2.36E+11	82 (0-174901)	90.3588
**K26K-PN66E8_metastasis21-01**	2.12E+09	1.78E+09	2.34E+11	76 (0-218458)	90.3603
**K26K-PPGH4E_buffy-coat1**	2.02E+09	1.8E+09	2.41E+11	84 (0-174022)	90.4672
**K26K-PPGH4E_metastasis12**	2.06E+09	1.78E+09	2.38E+11	81 (0-119763)	90.4553
**K26K-PT3E2X_buffy-coat1-01**	2.05E+09	1.76E+09	2.35E+11	82 (0-228506)	90.4313
**K26K-PT3E2X_metastasis11-01**	1.87E+09	1.61E+09	2.15E+11	71 (0-153489)	90.3487
**K26K-PYFRR7_buffy-coat2-01**	2.09E+09	1.78E+09	2.36E+11	82 (0-370467)	90.4404
**K26K-PYFRR7_metastasis11-01**	2.08E+09	1.77E+09	2.36E+11	82 (0-405370)	90.4431
**K26K-Q25AYS_buffy-coat1-01**	1.97E+09	1.7E+09	2.31E+11	81 (0-134154)	90.3885
**K26K-Q25AYS_metastasis11-01**	2.01E+09	1.8E+09	2.43E+11	88 (0-362993)	90.1964
**K26K-Q4JZ17_buffy-coat3**	1.96E+09	1.74E+09	2.36E+11	82 (0-162312)	90.4385
**K26K-Q4JZ17_metastasis21**	1.97E+09	1.73E+09	2.34E+11	76 (0-141254)	90.4186
**K26K-Q5MCEW_buffy-coat1-01**	1.85E+09	1.65E+09	2.21E+11	77 (0-406054)	90.4023
**K26K-Q5MCEW_tumor12-01**	1.79E+09	1.6E+09	2.11E+11	70 (0-344107)	79.8524
**K26K-Q6TE19_buffy-coat2-01**	1.65E+09	1.45E+09	1.91E+11	67 (0-374764)	90.3417

**K26K-Q6TE19_metastasis12-01**	1.8E+09	1.58E+09	2.05E+11	73 (0-345938)	90.2889
**K26K-Q7QMGK_buffy-coat1-01-p**	3.91E+08	3.57E+08	3.13E+10	10 (0-51375)	55.1734
**K26K-Q7QMGK_metastasis13-01-p**	3.88E+08	1.95E+08	1.56E+10	4 (0-295016)	13.9321
**K26K-Q99D4V_buffy-coat1-01**	2.04E+09	1.84E+09	2.45E+11	82 (0-281694)	72.9669
**K26K-Q99D4V_metastasis14-01**	2.05E+09	1.82E+09	2.39E+11	79 (0-262587)	70.9973
**K26K-QA7GAW_buffy-coat1**	2.2E+09	1.78E+09	2.36E+11	83 (0-173096)	90.4428
**K26K-QA7GAW_metastasis11**	2.21E+09	1.76E+09	2.25E+11	76 (0-207119)	90.4088
**K26K-QBQ2UL_buffy-coat1-01**	1.87E+09	1.64E+09	2.2E+11	77 (0-203827)	90.4104
**K26K-QBQ2UL_tumor11-01**	1.78E+09	1.6E+09	2.15E+11	73 (0-178854)	90.3865
**K26K-QHUFXS_buffy-coat1-01**	1.81E+09	1.61E+09	2.16E+11	75 (0-559314)	90.2779
**K26K-QHUFXS_tumor11-01**	2.26E+09	2.03E+09	2.73E+11	74 (0-706627)	63.1454
**K26K-QL2LML_buffy-coat1-01**	1.99E+09	1.76E+09	2.29E+11	69 (0-451554)	57.3047
**K26K-QL2LML_metastasis21-01**	1.96E+09	1.7E+09	2.26E+11	61 (0-399058)	63.1775
**K26K-QRC8PF_buffy-coat1-01-p**	3E+08	2.64E+08	2.35E+10	8 (0-54288)	32.5372
**K26K-QRC8PF_tumor3-01-p**	3.05E+08	2.6E+08	2.32E+10	7 (0-56197)	31.4448
**K26K-QSDNXA_buffy-coat2**	1.93E+09	1.72E+09	2.3E+11	80 (0-357069)	90.3474
**K26K-QSDNXA_tumor11**	1.92E+09	1.72E+09	2.22E+11	0 (0-413918)	22.1724
**K26K-QVWA2W_buffy-coat2-01**	1.85E+09	1.66E+09	2.17E+11	76 (0-402796)	90.4297
**K26K-QVWA2W_metastasis11-01**	1.92E+09	1.73E+09	2.27E+11	73 (0-1392395)	90.3012
**K26K-QXX4LD_buffy-coat1**	2.02E+09	1.76E+09	2.32E+11	81 (0-464994)	90.359
**K26K-QXX4LD_metastasis12**	1.92E+09	1.71E+09	2.16E+11	72 (0-453671)	90.3301
**K26K-R3SK9S_buffy-coat2-01**	2.18E+09	1.88E+09	2.41E+11	84 (0-1453732)	90.3182
**K26K-R3SK9S_metastasis11-01**	2.2E+09	1.89E+09	2.42E+11	0 (0-663414)	42.555
**K26K-R4ATRT_buffy-coat2**	2.08E+09	1.77E+09	2.42E+11	0 (0-300609)	42.5757
**K26K-R4ATRT_tumor12**	2E+09	1.74E+09	2.36E+11	0 (0-264187)	43.0319
**K26K-R5DVUL_buffy-coat1-01**	2E+09	1.73E+09	2.33E+11	81 (0-269621)	90.4367
**K26K-R5DVUL_metastasis11-01**	1.98E+09	1.73E+09	2.32E+11	79 (0-211039)	90.3734
**K26K-R8A4NH_buffy-coat2-01**	1.98E+09	1.64E+09	2.2E+11	77 (0-361949)	90.3427
**K26K-R8A4NH_metastasis11-01**	1.72E+09	1.44E+09	1.91E+11	63 (0-283581)	90.2764
**K26K-R8KRU9_buffy-coat2**	2E+09	1.79E+09	2.42E+11	0 (0-203857)	11.3426
**K26K-R8KRU9_metastasis11**	1.95E+09	1.75E+09	2.34E+11	0 (0-191237)	12.5977
**K26K-RA678B_buffy-coat1-01**	2.47E+09	2.14E+09	2.86E+11	100 (0-718025)	90.3581
**K26K-RA678B_metastasis11-01**	2.75E+09	2.41E+09	3.23E+11	0 (0-742227)	10.8317
**K26K-RCX5HZ_buffy-coat1-01**	1.94E+09	1.71E+09	2.3E+11	80 (0-379725)	90.4238
**K26K-RCX5HZ_metastasis11-01**	2.01E+09	1.77E+09	2.39E+11	0 (0-324673)	14.6099
**K26K-RDH1KP_buffy-coat1-01**	1.97E+09	1.71E+09	2.29E+11	0 (0-278046)	11.1582
**K26K-RDH1KP_metastasis12-01**	1.9E+09	1.65E+09	2.19E+11	0 (0-322593)	9.9208

**K26K-RG33TL_buffy-coat1-01**	1.99E+09	1.76E+09	2.38E+11	82 (0-402873)	90.3404
**K26K-RG33TL_metastasis11-01**	1.98E+09	1.77E+09	2.38E+11	0 (0-14376)	7.075
**K26K-RJEPF5_buffy-coat1-01**	1.97E+09	1.75E+09	2.34E+11	82 (0-228341)	90.3551
**K26K-RJEPF5_metastasis11-01**	1.96E+09	1.76E+09	2.34E+11	71 (0-207115)	90.289
**K26K-RPNFY9_buffy-coat1-01**	1.97E+09	1.74E+09	2.35E+11	82 (0-162535)	90.3247
**K26K-RPNFY9_metastasis11-01**	1.93E+09	1.66E+09	2.21E+11	71 (0-140222)	90.2332
**K26K-RSNUH1_buffy-coat1-01**	1.91E+09	1.61E+09	2.16E+11	75 (0-279888)	90.4225
**K26K-RSNUH1_metastasis12-01**	1.91E+09	1.62E+09	2.19E+11	73 (0-269686)	90.4039
**K26K-RSYYVH_buffy-coat1-01**	2.07E+09	1.77E+09	2.34E+11	81 (0-656329)	90.3498
**K26K-RSYYVH_metastasis11-01**	2.1E+09	1.74E+09	2.29E+11	76 (0-266325)	90.327
**K26K-RWBB37_buffy-coat2**	2.14E+09	1.79E+09	2.44E+11	85 (0-220088)	90.4538
**K26K-RWBB37_metastasis11**	2.09E+09	1.79E+09	2.43E+11	84 (0-224888)	90.4346
**K26K-RX8HVN_buffy-coat1-01**	2.25E+09	1.99E+09	2.66E+11	93 (0-865567)	90.3016
**K26K-RX8HVN_metastasis12-01**	2.09E+09	1.86E+09	2.48E+11	85 (0-701420)	90.3084
**K26K-S2A6CJ_buffy-coat2-01**	1.89E+09	1.64E+09	2.21E+11	77 (0-350741)	90.319
**K26K-S2A6CJ_metastasis12-01**	1.99E+09	1.67E+09	2.2E+11	69 (0-352190)	90.1677
**K26K-S3CZ8N_buffy-coat1-01**	2.35E+09	2.11E+09	2.83E+11	99 (0-723189)	90.3439
**K26K-S3CZ8N_buffy-coat1-01-p**	2.94E+08	2.57E+08	2.3E+10	7 (0-61515)	30.9
**K26K-S3CZ8N_tumor11-01**	1.9E+09	1.73E+09	2.31E+11	78 (0-616481)	90.2798
**K26K-S3FTVP_buffy-coat1-01-p**	2.48E+08	2.22E+08	1.99E+10	6 (0-41429)	23.4412
**K26K-S3FTVP_tumor21-01-p**	3.45E+08	2.73E+08	2.29E+10	6 (0-57906)	25.1845
**K26K-SAUCL3_buffy-coat1-01**	2.37E+09	2.11E+09	2.81E+11	98 (0-971547)	90.3919
**K26K-SAUCL3_metastasis11-01**	2.1E+09	1.88E+09	2.48E+11	83 (0-957364)	90.2954
**K26K-SD4V5Q_buffy-coat1-01**	1.98E+09	1.68E+09	2.28E+11	80 (0-326426)	90.3207
**K26K-SD4V5Q_tumor12-01**	2E+09	1.71E+09	2.25E+11	76 (0-375979)	90.3068
**K26K-SJGG4M_buffy-coat2**	1.92E+09	1.72E+09	2.33E+11	81 (0-176916)	90.4395
**K26K-SJGG4M_metastasis11**	1.77E+09	1.59E+09	2.13E+11	70 (0-151256)	90.3493
**K26K-SKGL7S_buffy-coat2-01-p**	2.75E+08	2.49E+08	2.23E+10	7 (0-50522)	29.2524
**K26K-SKGL7S_metastasis13-01-p**	2.67E+08	2.33E+08	2.06E+10	6 (0-57163)	23.9506
**K26K-SMQCAH_buffy-coat1-01**	2.54E+09	2.26E+09	3.03E+11	105 (0-599052)	90.4006
**K26K-SMQCAH_metastasis11-01**	2.31E+09	2.07E+09	2.79E+11	83 (0-825828)	90.244
**K26K-SMQDUN_buffy-coat2-01**	2.1E+09	1.76E+09	2.38E+11	83 (0-258752)	90.3252
**K26K-SMQDUN_metastasis11-01**	2E+09	1.68E+09	2.22E+11	68 (0-232303)	90.2506
**K26K-SNG1R3_buffy-coat1-01**	2.14E+09	1.79E+09	2.3E+11	80 (0-2087663)	90.3681
**K26K-SNG1R3_metastasis11-01**	2.05E+09	1.79E+09	2.4E+11	81 (0-371343)	90.3844
**K26K-SNVUBF_buffy-coat2-01**	1.91E+09	1.78E+09	2.39E+11	83 (0-412094)	90.3268
**K26K-SNVUBF_metastasis21-01**	1.23E+09	1.16E+09	1.55E+11	52 (0-376208)	90.1716

**K26K-SSBAF2_buffy-coat1**	1.84E+09	1.66E+09	2.23E+11	78 (0-102108)	90.4006
**K26K-SSBAF2_metastasis11**	1.86E+09	1.67E+09	2.22E+11	71 (0-136334)	90.3478
**K26K-SU166F_buffy-coat2**	1.93E+09	1.67E+09	2.25E+11	79 (0-450211)	90.3409
**K26K-SU166F_metastasis11**	1.97E+09	1.73E+09	2.31E+11	75 (0-477480)	90.339
**K26K-SU24BN_buffy-coat1-01**	2.14E+09	1.88E+09	2.49E+11	86 (0-265584)	90.3469
**K26K-SU24BN_metastasis11-01**	2.19E+09	1.91E+09	2.53E+11	82 (0-2030375)	90.286
**K26K-SV7281_buffy-coat2-01**	1.74E+09	1.52E+09	2.03E+11	71 (0-202958)	90.2929
**K26K-SV7281_metastasis11-01**	1.91E+09	1.69E+09	2.28E+11	76 (0-225935)	90.2704
**K26K-SWYTUR_buffy-coat1-01**	2.2E+09	1.96E+09	2.63E+11	92 (0-683532)	90.3676
**K26K-SWYTUR_metastasis11-01**	2.53E+09	2.24E+09	3E+11	96 (0-833883)	90.3279
**K26K-SXFM78_buffy-coat2-01**	2.18E+09	1.88E+09	2.45E+11	86 (0-2357078)	90.3036
**K26K-SXFM78_metastasis12-01**	2.15E+09	1.9E+09	2.55E+11	86 (0-318255)	90.3042
**K26K-T3FB3U_buffy-coat2-01**	2.04E+09	1.7E+09	2.26E+11	79 (0-352699)	90.355
**K26K-T3FB3U_tumor11-01**	2.07E+09	1.69E+09	2.27E+11	70 (0-406426)	90.2589
**K26K-T7EFJK_buffy-coat1-01**	2.13E+09	1.94E+09	2.6E+11	91 (0-921356)	90.3788
**K26K-T7EFJK_metastasis11-01**	2.31E+09	2.07E+09	2.77E+11	94 (0-1256996)	90.3896
**K26K-TABQ88_buffy-coat1-01**	2.01E+09	1.73E+09	2.33E+11	81 (0-278915)	90.3427
**K26K-TABQ88_metastasis11-01**	1.99E+09	1.71E+09	2.29E+11	78 (0-216869)	90.3232
**K26K-TBG1FN_buffy-coat1-01**	2.14E+09	1.84E+09	2.47E+11	86 (0-440662)	90.3469
**K26K-TBG1FN_metastasis11-01**	2.09E+09	1.81E+09	2.48E+11	84 (0-396298)	90.3212
**K26K-TF7NJ1_buffy-coat1-01**	1.95E+09	1.74E+09	2.28E+11	78 (0-324500)	90.3967
**K26K-TF7NJ1_metastasis12-01**	1.89E+09	1.68E+09	2.19E+11	63 (0-277508)	66.2117
**K26K-TG5T5N_buffy-coat1**	1.89E+09	1.68E+09	2.24E+11	78 (0-398518)	90.3024
**K26K-TG5T5N_metastasis11**	1.89E+09	1.69E+09	2.24E+11	77 (0-715348)	90.2833
**K26K-TL7317_buffy-coat1**	1.92E+09	1.72E+09	2.26E+11	79 (0-241454)	90.4522
**K26K-TL7317_metastasis11**	1.87E+09	1.65E+09	2.17E+11	72 (0-206419)	90.3964
**K26K-TMX9UN_buffy-coat2**	1.98E+09	1.69E+09	2.26E+11	79 (0-264799)	90.399
**K26K-TMX9UN_tumor1**	1.99E+09	1.69E+09	2.26E+11	72 (0-210526)	90.3424
**K26K-TTTSQQ_buffy-coat2-01**	2.13E+09	1.83E+09	2.49E+11	87 (0-618445)	90.3527
**K26K-TTTSQQ_metastasis11-01**	2.12E+09	1.8E+09	2.44E+11	84 (0-610585)	90.3345
**K26K-U13K48_buffy-coat2**	2.11E+09	1.8E+09	2.39E+11	83 (0-345144)	90.5057
**K26K-U13K48_metastasis11**	2.08E+09	1.53E+09	1.99E+11	68 (0-320177)	90.4182
**K26K-U1CUNE_buffy-coat1-01**	1.98E+09	1.75E+09	2.35E+11	82 (0-443535)	90.3645
**K26K-U1CUNE_metastasis11-01**	2.16E+09	1.83E+09	2.41E+11	83 (0-410924)	90.3797
**K26K-U3ERTU_buffy-coat2-01**	1.79E+09	1.58E+09	2.14E+11	75 (0-369672)	90.3269
**K26K-U3ERTU_metastasis11-01**	2.06E+09	1.76E+09	2.37E+11	77 (0-383312)	90.2989
**K26K-U48257_buffy-coat2**	2.03E+09	1.75E+09	2.32E+11	81 (0-1163983)	90.3762
**K26K-U48257_tumor11**	2.01E+09	1.72E+09	2.33E+11	70 (0-178868)	90.2673

**K26K-U9L51W_buffy-coat1-01-p**	2.46E+08	2.2E+08	1.98E+10	6 (0-43814)	20.3913
**K26K-U9L51W_metastasis13-01-p**	2.71E+08	2.31E+08	2.07E+10	6 (0-47035)	23.2554
**K26K-UGZH2U_buffy-coat2-01**	1.99E+09	1.7E+09	2.23E+11	78 (0-368005)	90.3049
**K26K-UGZH2U_metastasis11-01**	1.88E+09	1.66E+09	2.22E+11	76 (0-651506)	90.2499
**K26K-ULPXJ3_buffy-coat1-02**	1.9E+09	1.75E+09	2.35E+11	82 (0-436989)	90.3489
**K26K-ULPXJ3_metastasis22-01**	2.03E+09	1.84E+09	2.48E+11	76 (0-429542)	90.286
**K26K-UVAXJ4_buffy-coat2**	1.86E+09	1.66E+09	2.23E+11	78 (0-187356)	90.4217
**K26K-UVAXJ4_metastasis11**	1.8E+09	1.62E+09	2.18E+11	71 (0-151928)	90.3876
**K26K-UWDBJ5_buffy-coat1-01**	2.01E+09	1.72E+09	2.3E+11	80 (0-445990)	90.3423
**K26K-UWDBJ5_tumor12-01**	2.05E+09	1.75E+09	2.29E+11	74 (0-1680787)	90.275
**K26K-UWRHM6_metastasis11-01**	2.02E+09	1.86E+09	2.49E+11	82 (0-639059)	90.2455
**K26K-UY56QP_buffy-coat2**	2.05E+09	1.78E+09	2.39E+11	84 (0-215050)	90.3557
**K26K-UY56QP_metastasis11**	2.08E+09	1.83E+09	2.35E+11	80 (0-1096292)	90.3357
**K26K-UYPA78_buffy-coat2-01**	2.04E+09	1.79E+09	2.41E+11	84 (0-663206)	90.3907
**K26K-UYPA78_tumor11-01**	1.9E+09	1.69E+09	2.26E+11	79 (0-233547)	90.3991
**K26K-UZ6G9R_buffy-coat1-01**	2.14E+09	1.94E+09	2.61E+11	91 (0-664689)	90.3904
**K26K-UZ6G9R_tumor11-01**	2.32E+09	2.07E+09	2.79E+11	94 (0-455131)	90.4127
**K26K-V2PF6M_buffy-coat2**	2.05E+09	1.69E+09	2.25E+11	78 (0-451252)	90.4729
**K26K-V2PF6M_metastasis12**	2.03E+09	1.7E+09	2.27E+11	71 (0-294913)	90.1255
**K26K-V3TLD6_buffy-coat2**	1.92E+09	1.72E+09	2.26E+11	79 (0-678001)	90.329
**K26K-V3TLD6_metastasis11**	1.88E+09	1.67E+09	2.24E+11	75 (0-659309)	90.3456
**K26K-VAHD5J_buffy-coat1**	1.78E+09	1.61E+09	2.14E+11	75 (0-152686)	90.4033
**K26K-VAHD5J_metastasis12**	1.78E+09	1.59E+09	2.06E+11	62 (0-154154)	90.3313
**K26K-VBGZC7_buffy-coat1-01**	2.04E+09	1.73E+09	2.35E+11	82 (0-324666)	90.437
**K26K-VBGZC7_metastasis11-01**	1.96E+09	1.68E+09	2.25E+11	76 (0-311490)	90.3902
**K26K-VDQXWG_buffy-coat1-01**	1.96E+09	1.7E+09	2.29E+11	80 (0-250091)	90.4396
**K26K-VDQXWG_tumor11-01**	1.98E+09	1.7E+09	2.3E+11	77 (0-236983)	90.431
**K26K-VDWGQA_buffy-coat1-01**	1.53E+09	1.43E+09	1.91E+11	66 (0-394582)	90.2607
**K26K-VDWGQA_metastasis12-01**	2.96E+09	2.7E+09	3.59E+11	125 (0-817873)	90.4054
**K26K-VJ58WY_buffy-coat1-01-p**	2.6E+08	2.33E+08	2.08E+10	7 (0-48755)	23.6518
**K26K-VJ58WY_metastasis13-01-p**	3.56E+08	2.71E+08	2.36E+10	6 (0-68314)	31.9511
**K26K-VLD15Q_buffy-coat2-01**	2.03E+09	1.8E+09	2.42E+11	85 (0-373389)	90.3998
**K26K-VLD15Q_metastasis11-01**	1.91E+09	1.73E+09	2.31E+11	78 (0-710530)	90.402
**K26K-VR4F9W_buffy-coat1-01-p**	3.35E+08	3.07E+08	2.74E+10	9 (0-47599)	44.8459
**K26K-VR4F9W_metastasis13-01-p**	3.2E+08	2.77E+08	2.38E+10	7 (0-56625)	31.6835
**K26K-VSQ56S_buffy-coat1-01**	2E+09	1.74E+09	2.33E+11	81 (0-317863)	90.4557

**K26K-VSQ56S_tumor11-01**	2.04E+09	1.76E+09	2.36E+11	81 (0-363435)	90.4513
**K26K-VWASDF_buffy-coat1-01**	2.02E+09	1.75E+09	2.38E+11	83 (0-384589)	90.363
**K26K-VWASDF_tumor12-01**	2.06E+09	1.79E+09	2.43E+11	84 (0-336651)	90.3598
**K26K-WAMNC8_buffy-coat1-01**	1.9E+09	1.62E+09	2.18E+11	76 (0-237828)	90.4199
**K26K-WAMNC8_metastasis12-01**	1.87E+09	1.6E+09	2.14E+11	69 (0-210270)	90.4015
**K26K-WDFWZM_buffy-coat1-01**	1.77E+09	1.61E+09	2.16E+11	75 (0-525476)	90.318
**K26K-WDFWZM_metastasis12-01**	1.82E+09	1.66E+09	2.23E+11	76 (0-415370)	90.3004
**K26K-WHAFAB_buffy-coat1**	1.92E+09	1.62E+09	2.18E+11	76 (0-195438)	90.4304
**K26K-WHAFAB_metastasis11**	1.89E+09	1.6E+09	2.14E+11	70 (0-193379)	90.4138
**K26K-WLLBUL_buffy-coat2**	1.87E+09	1.67E+09	2.25E+11	79 (0-161963)	90.4231
**K26K-WLLBUL_metastasis12**	1.83E+09	1.58E+09	2.1E+11	74 (0-133041)	90.3736
**K26K-WPZ6MB_buffy-coat2**	2.08E+09	1.77E+09	2.29E+11	80 (0-815336)	90.3451
**K26K-WPZ6MB_metastasis11**	1.93E+09	1.69E+09	2.24E+11	77 (0-420123)	90.4516
**K26K-WQ6ZNA_buffy-coat2-02**	1.74E+09	1.53E+09	2.07E+11	72 (0-360678)	90.3208
**K26K-WQ6ZNA_metastasis11-01**	1.78E+09	1.58E+09	2.14E+11	71 (0-341256)	90.2571
**K26K-WS87L3_buffy-coat2-01**	2.09E+09	1.73E+09	2.29E+11	80 (0-274563)	90.3339
**K26K-WS87L3_metastasis11-01**	2.11E+09	1.75E+09	2.24E+11	74 (0-1756217)	90.3282
**K26K-WSBAGM_buffy-coat1-01**	2.03E+09	1.79E+09	2.4E+11	84 (0-245892)	90.4419
**K26K-WSBAGM_tumor22-01**	2.07E+09	1.79E+09	2.41E+11	84 (0-280880)	90.4001
**K26K-X9NVL2_buffy-coat2-01**	2.07E+09	1.75E+09	2.29E+11	80 (0-357718)	90.283
**K26K-X9NVL2_metastasis11-01**	2.15E+09	1.78E+09	2.36E+11	73 (0-412394)	90.2566
**K26K-XAEMKV_buffy-coat2**	2.14E+09	1.84E+09	2.48E+11	87 (0-322458)	90.4939
**K26K-XAEMKV_metastasis11**	2.08E+09	1.81E+09	2.4E+11	82 (0-382834)	90.4843
**K26K-XBFHQ2_buffy-coat1-02**	2.18E+09	1.9E+09	2.56E+11	89 (0-363368)	90.3439
**K26K-XBFHQ2_metastasis12-01**	2.17E+09	1.87E+09	2.53E+11	86 (0-343698)	90.3066
**K26K-XBFN4M_buffy-coat2**	2.13E+09	1.8E+09	2.44E+11	85 (0-228434)	90.4615
**K26K-XBFN4M_tumor11**	2.18E+09	1.83E+09	2.46E+11	77 (0-203596)	90.4533
**K26K-XBX9RY_buffy-coat2**	1.9E+09	1.64E+09	2.18E+11	76 (0-233597)	90.3339
**K26K-XBX9RY_metastasis11**	2.07E+09	1.79E+09	2.33E+11	69 (0-385411)	90.2984
**K26K-XEKNVJ_buffy-coat2**	1.99E+09	1.71E+09	2.32E+11	81 (0-210727)	90.4192
**K26K-XEKNVJ_metastasis1**	2E+09	1.72E+09	2.27E+11	79 (0-197868)	90.3971
**K26K-XEU9W5_buffy-coat2-01**	2.06E+09	1.81E+09	2.43E+11	85 (0-310247)	90.4389
**K26K-XEU9W5_metastasis11-01**	1.89E+09	1.68E+09	2.26E+11	78 (0-261506)	90.3868
**K26K-XF7MPU_buffy-coat1-01**	2.13E+09	1.81E+09	2.4E+11	84 (0-401537)	90.4646
**K26K-XF7MPU_metastasis11-01**	2.1E+09	1.81E+09	2.4E+11	81 (0-311161)	90.43
**K26K-XKVM9V_buffy-coat2**	1.9E+09	1.68E+09	2.3E+11	80 (0-125332)	90.4304
**K26K-XKVM9V_metastasis11**	1.97E+09	1.75E+09	2.33E+11	81 (0-151247)	90.4543

**K26K-XS277Z_buffy-coat2**	2.16E+09	1.82E+09	2.43E+11	85 (0-1470682)	90.4832
**K26K-XS277Z_metastasis11**	2.21E+09	1.88E+09	2.48E+11	82 (0-241416)	90.4667
**K26K-XU7ND6_buffy-coat2-01**	2.12E+09	1.82E+09	2.3E+11	80 (0-246267)	90.3475
**K26K-XU7ND6_metastasis11-01**	2.09E+09	1.8E+09	2.39E+11	81 (0-259243)	90.3865
**K26K-XVLCD2_buffy-coat2-01**	2.02E+09	1.7E+09	2.26E+11	79 (0-331088)	90.3783
**K26K-XVLCD2_tumor11-01**	2.08E+09	1.76E+09	2.37E+11	74 (0-281357)	90.2999
**K26K-XXHLCZ_metastasis11-01**	2.27E+09	2.01E+09	2.7E+11	91 (0-749659)	90.3728
**K26K-Y1N726_buffy-coat1-01**	1.98E+09	1.75E+09	2.31E+11	80 (0-290905)	90.355
**K26K-Y1N726_metastasis11-01**	1.94E+09	1.65E+09	2.14E+11	65 (0-259006)	85.3249
**K26K-Y2QMVE_buffy-coat1-01**	2.91E+09	2.56E+09	3.41E+11	119 (0-586101)	90.3908
**K26K-Y2QMVE_metastasis11-01**	2.5E+09	2.23E+09	2.95E+11	95 (0-709248)	90.2999
**K26K-Y41ABR_buffy-coat1-01**	2.16E+09	1.85E+09	2.44E+11	85 (0-668606)	90.4598
**K26K-Y41ABR_metastasis11-01**	2.06E+09	1.8E+09	2.4E+11	76 (0-391045)	90.3243
**K26K-Y665TN_buffy-coat1-01**	2.14E+09	1.85E+09	2.49E+11	87 (0-331066)	90.3848
**K26K-Y665TN_tumor11-01**	2.09E+09	1.78E+09	2.35E+11	72 (0-327571)	90.3221
**K26K-Y6G3UY_buffy-coat2**	1.96E+09	1.71E+09	2.31E+11	81 (0-157731)	90.4382
**K26K-Y6G3UY_metastasis11**	1.85E+09	1.68E+09	2.24E+11	76 (0-127664)	90.4116
**K26K-Y74RYK_buffy-coat1-01**	1.96E+09	1.7E+09	2.28E+11	79 (0-331800)	90.3961
**K26K-Y74RYK_metastasis11-01**	1.99E+09	1.69E+09	2.25E+11	76 (0-315649)	90.3616
**K26K-Y7EELU_buffy-coat1-01**	1.92E+09	1.7E+09	2.22E+11	66 (0-303134)	90.3329
**K26K-Y7EELU_metastasis12-01**	2.01E+09	1.79E+09	2.18E+11	45 (0-393326)	87.2879
**K26K-Y8ATLQ_buffy-coat2**	2.08E+09	1.74E+09	2.32E+11	81 (0-614760)	90.3517
**K26K-Y8ATLQ_metastasis11**	2.07E+09	1.72E+09	2.28E+11	75 (0-547176)	90.345
**K26K-Y9BDCK_buffy-coat2**	1.9E+09	1.7E+09	2.33E+11	81 (0-159560)	90.4339
**K26K-Y9BDCK_metastasis11**	1.91E+09	1.67E+09	2.25E+11	75 (0-161328)	90.3781
**K26K-YA19QK_buffy-coat1-01**	1.94E+09	1.73E+09	2.3E+11	80 (0-194381)	90.3278
**K26K-YA19QK_metastasis11-01**	1.85E+09	1.66E+09	2.21E+11	74 (0-185903)	90.2834
**K26K-YB2V81_buffy-coat2**	1.68E+09	1.28E+09	1.69E+11	59 (0-225074)	90.3477
**K26K-YB2V81_metastasis11**	1.56E+09	1.44E+09	1.94E+11	64 (0-164149)	90.3496
**K26K-YF7GYQ_buffy-coat2-01**	2.02E+09	1.76E+09	2.33E+11	81 (0-480022)	90.3611
**K26K-YF7GYQ_tumor11-01**	2.81E+09	2.39E+09	3.17E+11	109 (0-349357)	90.4223
**K26K-YF9FHJ_buffy-coat1-01**	1.91E+09	1.69E+09	2.26E+11	79 (0-282675)	90.4397
**K26K-YF9FHJ_metastasis11-01**	1.99E+09	1.72E+09	2.3E+11	75 (0-312736)	90.4051
**K26K-YFJGXZ_buffy-coat1-01**	2.12E+09	1.71E+09	2.31E+11	80 (0-135435)	90.3718
**K26K-YFJGXZ_tumor11-01**	2.13E+09	1.78E+09	2.36E+11	78 (0-169103)	90.3525
**K26K-YJ9GCJ_buffy-coat1-01**	2.4E+09	2.19E+09	2.94E+11	103 (0-766896)	90.3971
**K26K-YJ9GCJ_metastasis11-01**	2.64E+09	2.38E+09	3.22E+11	103 (0-868169)	90.3491

**K26K-YJB543_buffy-coat2**	2.18E+09	1.87E+09	2.45E+11	86 (0-886718)	90.3742
**K26K-YJB543_metastasis11**	2.07E+09	1.78E+09	2.34E+11	80 (0-969915)	90.441
**K26K-YRWDKX_buffy-coat1-01**	2.07E+09	1.85E+09	2.41E+11	84 (0-303004)	90.4467
**K26K-YRWDKX_tumor11-01**	2.03E+09	1.78E+09	2.34E+11	69 (0-243216)	90.3214
**K26K-YU91XW_buffy-coat2**	2.12E+09	1.8E+09	2.42E+11	85 (0-318488)	90.3843
**K26K-YU91XW_metastasis11**	2.11E+09	1.83E+09	2.45E+11	82 (0-300428)	90.3631
**K26K-YXC7H4_buffy-coat2-01**	2.04E+09	1.76E+09	2.36E+11	82 (0-511531)	90.3377
**K26K-YXC7H4_tumor12-01**	2.04E+09	1.78E+09	2.4E+11	78 (0-413385)	90.3244
**K26K-YYMX7V_buffy-coat2**	2.09E+09	1.79E+09	2.44E+11	85 (0-256789)	90.4736
**K26K-YYMX7V_metastasis11**	2.13E+09	1.81E+09	2.45E+11	85 (0-246411)	90.4215
**K26K-YYP1DV_buffy-coat2-01**	2.06E+09	1.74E+09	2.3E+11	80 (0-428434)	90.3743
**K26K-YYP1DV_metastasis11-01**	2.05E+09	1.75E+09	2.34E+11	71 (0-426809)	89.9051
**K26K-Z3LWUS_buffy-coat2**	1.9E+09	1.69E+09	2.19E+11	76 (0-422816)	90.3543
**K26K-Z3LWUS_metastasis11**	1.89E+09	1.67E+09	2.21E+11	76 (0-419650)	90.3464
**K26K-Z559NG_buffy-coat1**	2.09E+09	1.8E+09	2.42E+11	85 (0-141928)	90.5005
**K26K-Z559NG_metastasis11**	2.08E+09	1.8E+09	2.36E+11	74 (0-131151)	90.439
**K26K-Z6VH73_buffy-coat2**	2.09E+09	1.66E+09	2.23E+11	78 (0-258743)	90.4651
**K26K-Z6VH73_metastasis11**	2.04E+09	1.63E+09	2.2E+11	76 (0-195533)	90.4566
**K26K-Z8XJY9_buffy-coat2-01**	2.13E+09	1.78E+09	2.41E+11	84 (0-682765)	90.3561
**K26K-Z8XJY9_metastasis11-01**	2.14E+09	1.85E+09	2.54E+11	76 (0-149777)	90.2314
**K26K-Z8Z4HX_buffy-coat1**	1.86E+09	1.67E+09	2.27E+11	79 (0-100473)	90.4136
**K26K-Z8Z4HX_metastasis11**	1.78E+09	1.6E+09	2.15E+11	73 (0-95841)	90.3946
**K26K-ZCXQU4_buffy-coat2-01**	2.12E+09	1.83E+09	2.44E+11	85 (0-358168)	90.3578
**K26K-ZCXQU4_metastasis12-01**	2.04E+09	1.77E+09	2.36E+11	79 (0-363963)	90.33
**K26K-ZDRXB2_buffy-coat2**	1.96E+09	1.69E+09	2.3E+11	80 (0-229731)	90.4076
**K26K-ZDRXB2_tumor2**	1.96E+09	1.68E+09	2.27E+11	78 (0-223765)	90.3942
**K26K-ZDUSLY_buffy-coat1-01**	2.25E+09	2.04E+09	2.74E+11	95 (0-664447)	90.3936
**K26K-ZDUSLY_metastasis12-01**	2.07E+09	1.88E+09	2.52E+11	84 (0-605388)	90.3426
**K26K-ZFF7VY_buffy-coat2**	2.07E+09	1.77E+09	2.4E+11	84 (0-235808)	90.3725
**K26K-ZFF7VY_tumor16**	2.03E+09	1.78E+09	2.38E+11	81 (0-528136)	90.3694
**K26K-ZH5GAP_buffy-coat2-01**	2.08E+09	1.87E+09	2.49E+11	87 (0-240541)	90.3912
**K26K-ZH5GAP_tumor11-01**	1.9E+09	1.71E+09	2.24E+11	73 (0-227560)	90.3277
**K26K-ZUWXZY_buffy-coat2**	1.86E+09	1.68E+09	2.25E+11	79 (0-283377)	90.4297
**K26K-ZUWXZY_metastasis11**	1.88E+09	1.68E+09	2.24E+11	77 (0-320084)	90.423
**K26K-ZWJDB4_buffy-coat1-01-p**	1.97E+08	1.75E+08	1.56E+10	5 (0-41718)	9.64719
**K26K-ZWJDB4_metastasis23-01-p**	2.97E+08	1.75E+08	1.39E+10	4 (0-164533)	9.7285
**K26K-ZZHJUA_buffy-coat1-01**	2.16E+09	1.85E+09	2.47E+11	86 (0-402241)	90.359
**K26K-ZZHJUA_metastasis11-01**	2.13E+09	1.86E+09	2.47E+11	82 (0-380608)	90.3446

Whole exome sequencing

For the exome enrichment we used Agilent7 Sureselect Human All Exon without UTRs (35804808) and Agilent Sureselect Human All Exon V5+UTR (74569526).

Sample ID	Total number of sequenced reads	Total number of uniquely mapped non duplicate reads	Total number of covered bases	Median coverage (and range) per base	Percentage of targeted bases with coverage >=10
**K26K-1F29VR_buffy-coat1-01-p**	3.2E+08	2.36E+08	74569526	167 (0.0-10064.0)	98.83
**K26K-1F29VR_metastasis13-01-p**	4.95E+08	2.7E+08	74569526	176 (0.0-25291.0)	98.75
**K26K-5M96VM_buffy-coat1-01-p**	2.9E+08	2.39E+08	74569526	166 (0.0-10583.0)	98.9
**K26K-5M96VM_metastasis13-01-p**	5.57E+08	3.28E+08	74569526	209 (0.0-16550.0)	99.01
**K26K-8S2LMY_buffy-coat1-01-p**	2.86E+08	2.05E+08	74569526	142 (0.0-15846.0)	98.82
**K26K-8S2LMY_metastasis13-01-p**	7.23E+08	3.95E+08	74569526	236 (0.0-26195.0)	99.12
**K26K-8VYLK7_buffy-coat1-01-p**	4.47E+08	3.37E+08	74569526	235 (0.0-18176.0)	99.11
**K26K-8VYLK7_metastasis11-01-p**	4.39E+08	1.85E+08	74569526	127 (0.0-18897.0)	98.63
**K26K-AT59U7_buffy-coat1-01-p**	3.46E+08	2.5E+08	74569526	173 (0.0-8961.0)	99.03
**K26K-AT59U7_metastasis13-01-p**	4.97E+08	2.85E+08	74569526	166 (0.0-13561.0)	99
**K26K-B21ADR_buffy-coat1-01-p**	4.02E+08	2.92E+08	74569526	200 (0.0-13613.0)	99.07
**K26K-B21ADR_tumor11-01-p**	4.59E+08	3.02E+08	74569526	180 (0.0-18259.0)	98.37
**K26K-B5D94J_buffy-coat1-01-p**	2.71E+08	2.17E+08	74569526	144 (0.0-11420.0)	98.71
**K26K-B5D94J_metastasis11-01-p**	6.02E+08	3.2E+08	74569526	214 (0.0-15195.0)	99
**K26K-BF6J54_buffy-coat1-01-p**	3.69E+08	2.42E+08	74569526	170 (0.0-10727.0)	98.77
**K26K-BF6J54_tumor12-01-p**	5.12E+08	2.83E+08	74569526	130 (0.0-22470.0)	98.74
**K26K-C34LTG_buffy-coat1-01-p**	2.65E+08	2.08E+08	74569526	144 (0.0-14684.0)	98.78
**K26K-C34LTG_metastasis12-01-p**	4.58E+08	3.54E+08	74569526	243 (0.0-21183.0)	99.12

**K26K-CRA69B_buffy-coat1-01-p**	2.94E+08	2.14E+08	74569526	153 (0.0-10489.0)	98.95
**K26K-CRA69B_metastasis13-01-p**	6.56E+08	2.62E+08	74569526	160 (0.0-14421.0)	98.99
**K26K-CVQCDD_buffy-coat1-01-p**	1.88E+08	1.57E+08	35804808	165 (0.0-5849.0)	97.22
**K26K-CVQCDD_metastasis11-01-p**	2.07E+08	1.65E+08	35804808	161 (0.0-6201.0)	97.07
**K26K-FHFEVH_buffy-coat2-01-p**	4.79E+08	3.65E+08	74569526	251 (0.0-12004.0)	99.03
**K26K-FHFEVH_tumor11-01-p**	5.54E+08	3.07E+08	74569526	216 (0.0-8933.0)	98.91
**K26K-J1JGYX_buffy-coat1-01-p2**	5.83E+08	4.39E+08	74569526	292 (0.0-18525.0)	99.09
**K26K-J1JGYX_metastasis1-01-p2**	8.16E+08	4.88E+08	74569526	334 (0.0-18741.0)	99.05
**K26K-JHFXVG_buffy-coat2-01-p**	4.07E+08	2.89E+08	74569526	204 (0.0-13387.0)	99.04
**K26K-JHFXVG_metastasis13-01-p**	4.49E+08	1.69E+08	74569526	109 (0.0-11169.0)	98.53
**K26K-JSP6N7_buffy-coat2-01-p**	1.63E+08	1.28E+08	35804808	137 (0.0-4842.0)	97.25
**K26K-JSP6N7_metastasis11-01-p**	2.72E+08	1.58E+08	35804808	140 (0.0-4091.0)	97.14
**K26K-KN2BCS_buffy-coat1-01-p**	3E+08	2.31E+08	74569526	159 (0.0-8339.0)	98.98
**K26K-KN2BCS_metastasis13-01-p**	6.25E+08	3.89E+08	74569526	236 (0.0-19442.0)	99.13
**K26K-L3MWGQ_buffy-coat2-01-p**	3.67E+08	2.54E+08	74569526	174 (0.0-13627.0)	98.91
**K26K-L3MWGQ_metastasis11-01-p**	4.71E+08	2.26E+08	74569526	116 (0.0-24698.0)	98.54
**K26K-LA3ZXV_buffy-coat1-01-p**	4.03E+08	3.03E+08	74569526	208 (0.0-14944.0)	99.09
**K26K-LA3ZXV_tumor13-01-p**	4.85E+08	3.39E+08	74569526	223 (0.0-14240.0)	99.11
**K26K-MLPAW4_buffy-coat2-01-p**	1.77E+08	1.42E+08	35804808	156 (0.0-5340.0)	97.14
**K26K-MLPAW4_tumor11-01-p**	2.41E+08	1.77E+08	35804808	146 (0.0-8332.0)	97.08
**K26K-MMXGSX_buffy-coat1-01-p**	4.63E+08	3.14E+08	74569526	222 (0.0-18827.0)	99.05
**K26K-MMXGSX_metastasis13-01-p**	3.71E+08	2.5E+08	74569526	167 (0.0-15302.0)	98.93
**K26K-Q39GUH_buffy-coat1**	1.22E+08	97242976	74569526	69 (0.0-8021.0)	97.9

**K26K-Q39GUH_metastasis11**	2.29E+08	1.61E+08	74569526	113 (0.0-10724.0)	98.48
**K26K-Q7QMGK_buffy-coat1-01-p**	5.38E+08	4.02E+08	74569526	272 (0.0-16243.0)	99.08
**K26K-Q7QMGK_metastasis13-01-p**	5.55E+08	1.84E+08	74569526	110 (0.0-15935.0)	98.04
**K26K-QRC8PF_buffy-coat1-01-p**	3.92E+08	2.93E+08	74569526	196 (0.0-15339.0)	99.06
**K26K-QRC8PF_tumor3-01-p**	3.76E+08	2.62E+08	74569526	174 (0.0-8975.0)	98.98
**K26K-S3CZ8N_buffy-coat1-01-p**	3.32E+08	2.53E+08	74569526	178 (0.0-12379.0)	99.02
**K26K-S3FTVP_buffy-coat1-01-p**	3.36E+08	2.42E+08	74569526	168 (0.0-15232.0)	98.7
**K26K-S3FTVP_tumor21-01-p**	7.27E+08	2.56E+08	74569526	125 (0.0-21784.0)	98.65
**K26K-SKGL7S_buffy-coat2-01-p**	4.4E+08	3.45E+08	74569526	241 (0.0-15294.0)	99.08
**K26K-SKGL7S_metastasis13-01-p**	4.61E+08	3.05E+08	74569526	202 (0.0-18088.0)	99.02
**K26K-U9L51W_buffy-coat1-01-p**	4.11E+08	2.92E+08	74569526	207 (0.0-15678.0)	99.07
**K26K-U9L51W_metastasis13-01-p**	4.29E+08	2.7E+08	74569526	187 (0.0-14352.0)	99.09
**K26K-VJ58WY_buffy-coat1-01-p**	3.04E+08	2.35E+08	74569526	169 (0.0-17168.0)	98.93
**K26K-VJ58WY_metastasis13-01-p**	6.19E+08	3.25E+08	74569526	218 (0.0-28262.0)	98.61
**K26K-VR4F9W_buffy-coat1-01-p**	5.91E+08	4.85E+08	74569526	335 (0.0-13285.0)	99.16
**K26K-VR4F9W_metastasis13-01-p**	6E+08	4.24E+08	74569526	272 (0.0-17683.0)	99.08
**K26K-ZWJDB4_buffy-coat1-01-p**	2.95E+08	2.19E+08	74569526	158 (0.0-9868.0)	98.91
**K26K-ZWJDB4_metastasis23-01-p**	2.62E+08	85057757	74569526	48 (0.0-8804.0)	95.67

RNA sequencing

Sample.ID.	Total number of sequenced reads	Total number of uniquely mapped reads	RNA integrity number RIN	Ratio of all reads aligned to rRNA regions to total uniquely mapped reads rRNA rate	Ratio of exon mapped reads to total uniquely mapped reads Expression Profile Efficiency	Total number of detected transcripts with reads >=1
**K26K-11UKEH_tumor12**	2.4E+08	2.19E+08	7.7	0.027403	0.872797	142802
**K26K-13FTNS_metastasis12**	1.83E+08	1.71E+08	9.4	0.012742	0.885755	142291
**K26K-19AFN7_tumor13**	2.58E+08	2.46E+08	9.5	0.002467	0.893091	146103
**K26K-1ER5BG_metastasis11**	2.77E+08	2.58E+08	9.7	0.002876	0.86961	153509
**K26K-1F29VR_metastasis13**	1.99E+08	1.52E+08	3.1	0.025458	0.666525	147165
**K26K-1F29VR_metastasis21**	1.65E+08	1.33E+08	7.3	0.018292	0.476966	135701
**K26K-1GUTR3_metastasis11**	2.58E+08	2.43E+08	9.1	0.002464	0.887622	147938
**K26K-1K6GZP_metastasis11**	1.88E+08	1.77E+08	8.4	0.006763	0.879013	146333
**K26K-1MDU5E_metastasis11**	3.08E+08	2.8E+08	7.5	0.019525	0.857693	145906
**K26K-1MDU5E_metastasis21**	3.77E+08	3.4E+08	7.2	0.02008	0.832763	146961
**K26K-1NV3L2_metastasis11**	2.19E+08	2.04E+08	7.6	0.010503	0.870964	148466
**K26K-1UGE3K_metastasis11**	2.01E+08	1.84E+08	8.3	0.009842	0.859604	143855
**K26K-1YV1NR_metastasis11**	2.28E+08	1.54E+08	7.9	0.012784	0.630726	138191
**K26K-1YXDJQ_metastasis11**	2.48E+08	2.26E+08	9.1	0.004081	0.845906	153371
**K26K-1YXDJQ_metastasis12**	1.08E+09	9.36E+08	9,4	0.003541	0.770902	162271
**K26K-25C1GA_metastasis11**	2.22E+08	2.05E+08	7.5	0.003708	0.849904	148835
**K26K-2ALFPG_metastasis12**	2.56E+08	2.38E+08	7.5	0.013587	0.867122	148300

**K26K-2ALFPG_metastasis21**	1.3E+08	1.13E+08	9.5	0.009726	0.768275	139701
**K26K-2ALFPG_tumor11**	1.9E+08	1.27E+08	6.8	0.019177	0.610614	139137
**K26K-2HQFG6_metastasis12**	2.52E+08	2.39E+08	7.2	0.012912	0.894183	149593
**K26K-2LSYUD_tumor11**	1.02E+09	7.28E+08	4.4	0.025269	0.640294	135799
**K26K-2WDLYE_tumor11**	4.07E+08	3.83E+08	8.1	0.02735	0.848263	155196
**K26K-372K7Q_metastasis11**	3.04E+08	2.77E+08	8	0.011542	0.853397	147706
**K26K-38J27P_metastasis11**	1.07E+09	9.57E+08	8.6	0.011393	0.813926	157576
**K26K-3F54SR_metastasis11**	2.12E+08	1.95E+08	9.1	0.009416	0.864675	151127
**K26K-3GWRN9_metastasis11**	1.97E+08	1.83E+08	7.4	0.003143	0.844177	147585
**K26K-3GWRN9_tumor21**	2.27E+08	2E+08	7.7	0.021042	0.683203	145324
**K26K-3J6HU5_tumor11**	2.36E+08	2.11E+08	8.20	0.026721	0.828175	147612
**K26K-3SMV2Y_metastasis11**	3.67E+08	3.4E+08	9.4	0.002931	0.844568	151386
**K26K-3SV5GJ_metastasis11**	2.58E+08	2.42E+08	7.9	0.016443	0.883256	149685
**K26K-3TN2B7_tumor11**	1.98E+08	1.79E+08	9.2	0.002271	0.833671	145216
**K26K-3UL22G_metastasis11**	2.77E+08	2.6E+08	8.4	0.013968	0.882779	145033
**K26K-3VGWL4_metastasis11**	2.77E+08	2.53E+08	6.5	0.010269	0.827021	150421
**K26K-3W9ZP5_metastasis43**	2.63E+08	2.4E+08	9.4	0.020608	0.852961	143862
**K26K-3Y6UFG_metastasis11**	1.06E+09	9.2E+08	7.5	0.09455	0.697957	158367
**K26K-43JZGQ_tumor12**	2.53E+08	2.36E+08	8.4	0.017843	0.890355	145277
**K26K-49T9ZE_metastasis11**	2.48E+08	2.34E+08	8.3	0.012394	0.895982	148838
**K26K-4AUP7Y_tumor11**	2.38E+08	1.86E+08	9.2	0.006542	0.745135	140275
**K26K-4JNSU8_metastasis11**	2.81E+08	2.45E+08	8	0.010007	0.820028	142305
**K26K-4JNSU8_metastasis22**	2.63E+08	2.47E+08	9.2	0.008394	0.871862	149338
**K26K-4L12DA_metastasis11**	2.18E+08	2.02E+08	6.9	0.016647	0.887807	138456

**K26K-4MUH3K_metastasis12**	1.9E+08	1.71E+08	7.8	0.017668	0.864214	140395
**K26K-4PEUSL_metastasis11**	1.02E+09	8.91E+08	9.1	0.003828	0.785076	157308
**K26K-4RJ5UK_metastasis12**	2.94E+08	2.74E+08	9.6	0.006323	0.853845	152447
**K26K-4UEVHA_tumor12**	1.97E+08	1.81E+08	8	0.005371	0.880143	141433
**K26K-4VP5ZM_metastasis22**	1.85E+08	1.66E+08	6.7	0.010801	0.72367	142590
**K26K-52RKM3_metastasis11**	2E+08	1.78E+08	6.9	0.04635	0.847975	141179
**K26K-53KLXE_metastasis12**	2.75E+08	2.54E+08	8	0.004053	0.885755	141297
**K26K-54UFHM_metastasis12**	1.05E+09	9.34E+08	5.3	0.014737	0.802391	157971
**K26K-595J2V_metastasis12**	2.31E+08	2.18E+08	9	0.006389	0.857431	152984
**K26K-59LEYZ_metastasis11**	1.05E+09	7.25E+08	7,3	0.040099	0.596074	151371
**K26K-5AJQY4_metastasis11**	2.94E+08	2.6E+08	8.5	0.014195	0.849383	144868
**K26K-5ELXUS_metastasis11**	2.59E+08	2.43E+08	7.4	0.007241	0.885551	148179
**K26K-5NNPD2_metastasis12**	2.39E+08	2.25E+08	8.8	0.004469	0.887532	150455
**K26K-5QQNGF_metastasis22**	1.93E+08	1.74E+08	8.4	0.007615	0.77554	144671
**K26K-5QQNGF_metastasis41**	1.93E+08	1.73E+08	8.5	0.006416	0.709975	139658
**K26K-5S8AT2_metastasis11**	2.51E+08	2.26E+08	7.6	0.011225	0.869012	138173
**K26K-5XRAND_metastasis12**	2.17E+08	1.99E+08	8.9	0.01182	0.876145	148548
**K26K-62B38Y_metastasis12**	3.18E+08	2.68E+08	8.3	0.009179	0.777229	141873
**K26K-65NG9T_tumor11**	2.54E+08	1.99E+08	7.8	0.005459	0.543969	136574
**K26K-6AR69Z_metastasis12**	3.29E+08	3.05E+08	8.5	0.011573	0.874323	150891
**K26K-6DAZ5U_metastasis12**	2.02E+08	1.91E+08	8.5	0.008877	0.882484	147910
**K26K-6FAWPW_metastasis11**	9.96E+08	8.79E+08	7.9	0.011469	0.79331	150433
**K26K-6FX6FM_metastasis11**	2.36E+08	2.09E+08	8.6	0.009782	0.787691	149672

**K26K-6LFEUV_tumor12**	2.8E+08	2.62E+08	9.1	0.005085	0.87082	150328
**K26K-6PJ115_metastasis11**	2.06E+08	1.9E+08	7.1	0.013241	0.873817	139822
**K26K-6PKB4F_metastasis11**	3.24E+08	2.9E+08	6,7	0.016388	0.816888	150791
**K26K-6QT98R_metastasis11**	2.94E+08	2.77E+08	8.3	0.005373	0.888716	152553
**K26K-6S8M2L_tumor11**	2.54E+08	2.38E+08	7	0.010004	0.880637	147242
**K26K-6SJWVZ_tumor11**	3.63E+08	3.34E+08	7.5	0.008066	0.85095	149500
**K26K-6T2U2T_metastasis11**	2.81E+08	2.59E+08	8	0.002988	0.864074	151414
**K26K-6TUAKJ_tumor11**	2.31E+08	1.91E+08	9	0.013912	0.778828	133327
**K26K-6Z62PR_metastasis11**	2.4E+08	2.27E+08	8.3	0.005903	0.8757	147190
**K26K-6Z62PR_metastasis23**	2.12E+08	1.22E+08	1.9	0.031089	0.327054	139239
**K26K-7ARCJQ_metastasis12**	2.54E+08	2.17E+08	8	0.028704	0.800445	147043
**K26K-7ARCJQ_metastasis22**	1.93E+08	1.74E+08	8.5	0.016761	0.777475	145821
**K26K-7ARCJQ_metastasis31**	2.35E+08	2.08E+08	6.5	0.023788	0.805451	141501
**K26K-7BQYTL_metastasis11**	2.62E+08	2.4E+08	6.1	0.008613	0.796388	141004
**K26K-7BQYTL_metastasis21**	1.43E+08	1.24E+08	9.6	0.023058	0.685388	138419
**K26K-7FVDMP_metastasis11**	2.66E+08	2.49E+08	9.9	0.00767	0.887832	150780
**K26K-7FVDMP_metastasis21**	4.95E+08	4.66E+08	9	0.007871	0.88078	157129
**K26K-7GHTVB_metastasis11**	2.6E+08	2.46E+08	9.2	0.002173	0.907947	143394
**K26K-7GHTVB_metastasis21**	2.91E+08	2.73E+08	9.3	0.002011	0.844477	144978
**K26K-7K2ESR_metastasis12**	2.46E+08	2.23E+08	8.4	0.013684	0.869886	143442
**K26K-7Q6MXJ_tumor11**	1.77E+08	1.63E+08	7.9	0.026985	0.863456	143174
**K26K-7UH38T_metastasis21**	1.55E+08	1.25E+08	9.5	0.018039	0.713449	141496
**K26K-7UH38T_tumor11**	1.73E+08	1.62E+08	9.1	0.012601	0.856429	144881

**K26K-7VJE74_metastasis11**	2.05E+08	1.91E+08	9	0.002906	0.831671	149046
**K26K-82VGF5_metastasis12**	2.94E+08	2.62E+08	8.7	0.02375	0.826443	148104
**K26K-82VGF5_tumor11**	3.08E+08	2.9E+08		0.007386	0.868495	149232
**K26K-89CAXB_tumor11**	2.32E+08	2.17E+08	9.1	0.011634	0.892855	143388
**K26K-8SNWXL_metastasis11**	2.4E+08	2.17E+08	8.7	0.008419	0.83638	146865
**K26K-8SNWXL_metastasis21**	1.59E+08	1.42E+08	9.9	0.007171	0.807337	138073
**K26K-8SNWXL_tumor11**	2.13E+08	1.97E+08	8.2	0.006091	0.861256	144163
**K26K-92LDBN_metastasis11**	2.54E+08	2.4E+08	8.9	0.008765	0.888044	146681
**K26K-9EASMF_metastasis12**	2.5E+08	2.19E+08	7.3	0.021989	0.822907	143876
**K26K-9G8979_metastasis11**	2.67E+08	2.52E+08	9	0.008128	0.852898	149717
**K26K-9KDVFY_metastasis11**	2.67E+08	2.33E+08	7.3	0.024826	0.790894	145248
**K26K-A1FE3E_metastasis11**	2.33E+08	1.81E+08	8.9	0.018544	0.723831	143439
**K26K-A3YEMT_metastasis12**	2.13E+08	2.02E+08	7.8	0.012224	0.907979	144928
**K26K-A3YEMT_metastasis21**	1.57E+08	1.19E+08	2.1	0.025701	0.323824	124616
**K26K-A96VU1_metastasis12**	2.44E+08	2.14E+08	7.5	0.049636	0.827471	141509
**K26K-AEC16Q_metastasis12**	2.41E+08	2.19E+08	5.8	0.020352	0.855979	150551
**K26K-AHDG8P_metastasis11**	2.18E+08	1.94E+08	7.3	0.012954	0.831571	144197
**K26K-AHDG8P_metastasis21**	2.25E+08	2.09E+08	9.5	0.002204	0.855218	144474
**K26K-ALSG3K_metastasis21**	2.73E+08	2.5E+08	8.5	0.013532	0.833071	148191
**K26K-AN7W62_metastasis11**	2.07E+08	1.6E+08	8.2	0.018489	0.729115	141280
**K26K-ARB5RQ_metastasis11**	3.06E+08	2.78E+08	8	0.013217	0.874765	143682
**K26K-ARB5RQ_tumor12**	2.7E+08	2.44E+08	7.5	0.0203	0.811799	144623
**K26K-AT59U7_metastasis13**	1.7E+08	1.34E+08	3.5	0.007663	0.712688	147223
**K26K-AVQZ6J_tumor11**	2.29E+08	2.08E+08	8	0.016564	0.867099	147560

**K26K-B21ADR_tumor11**	1.74E+08	1.14E+08	3.1	0.004752	0.443991	152310
**K26K-B3P4PS_tumor12**	2.41E+08	2.11E+08	7.5	0.016354	0.8298	137445
**K26K-B5VPJQ_metastasis11**	2.98E+08	2.19E+08	7.4	0.005526	0.699989	139307
**K26K-B5VPJQ_tumor11**	1.91E+08	1.79E+08		0.002385	0.90159	142030
**K26K-B5VPJQ_tumor22**	2.89E+08	2.55E+08	9.1	0.002504	0.820119	150953
**K26K-B8MUBJ_metastasis12**	2.38E+08	2.26E+08	9.8	0.003084	0.886801	149063
**K26K-BF6J54_tumor12**	2.02E+08	1.5E+08	2.4	0.023064	0.598494	146288
**K26K-BHSR57_metastasis11**	3.25E+08	3.01E+08	8.6	0.011783	0.855967	150724
**K26K-BHSR57_metastasis22**	2.36E+08	2E+08	8.7	0.012716	0.682039	140439
**K26K-BJVSF7_metastasis11**	2.16E+08	2.01E+08	7.3	0.007349	0.87231	145896
**K26K-BQAVRQ_metastasis12**	1.25E+08	1.16E+08	6.6	0.008111	0.880998	142046
**K26K-BRET3U_metastasis11**	2.24E+08	2.03E+08	9.1	0.018511	0.838588	143745
**K26K-BUNZZM_metastasis11**	1.96E+08	1.84E+08	6.9	0.007973	0.88043	144209
**K26K-BY9U8C_metastasis11**	2.42E+08	2.09E+08	6.6	0.023865	0.789565	145030
**K26K-BZC91B_metastasis11**	3.31E+08	3.05E+08	8.8	0.012424	0.855162	152633
**K26K-C1DQWG_tumor12**	2.55E+08	2.29E+08	8	0.021667	0.697821	145640
**K26K-C34LTG_metastasis12**	77838356	73416400	7	0.008401	0.894359	142971
**K26K-CEZTWE_metastasis12**	3.01E+08	2.41E+08	7.6	0.01864	0.762053	146122
**K26K-CHHDF2_metastasis11**	2.26E+08	1.55E+08	9.1	0.017137	0.619523	139396
**K26K-CHHDF2_metastasis22**	3.81E+08	3.55E+08	7.5	0.01082	0.867142	150684
**K26K-CK5DRG_metastasis11**	2.91E+08	2.74E+08	9.1	0.011279	0.875226	151447
**K26K-CTD3KC_metastasis11**	2.12E+08	1.94E+08	8.5	0.014691	0.851018	146600
**K26K-CTD3KC_metastasis21**	1.65E+08	1.53E+08	9.1	0.003553	0.841639	145084

**K26K-CVQCDD_metastasis11**	1.24E+08	67212926	3.7	0.018472	0.427265	142192
**K26K-CYERP8_tumor11**	3.03E+08	2.85E+08	9.7	0.003539	0.848281	152020
**K26K-D63727_tumor11**	2.17E+08	1.91E+08	8.5	0.026247	0.825612	146009
**K26K-D7A6KB_metastasis11**	2.52E+08	2.34E+08	7.5	0.014838	0.852135	147613
**K26K-DA2KDN_metastasis12**	2.01E+08	1.8E+08	8.5	0.015232	0.812062	144160
**K26K-DDJVUJ_metastasis12**	3.3E+08	3.1E+08	8.1	0.011484	0.892375	148696
**K26K-DX72G5_metastasis11**	2.31E+08	2.03E+08	8.8	0.021496	0.838684	142006
**K26K-E59Y51_metastasis11**	2.1E+08	1.97E+08	9.5	0.001732	0.840531	150128
**K26K-E6Q28L_tumor11**	2.72E+08	2.47E+08	7.2	0.007477	0.83764	152066
**K26K-E6QE8X_metastasis11**	2.43E+08	2.26E+08	8.7	0.005522	0.893617	147279
**K26K-E6QE8X_metastasis22**	2.29E+08	2.11E+08	9.2	0.002442	0.86595	145218
**K26K-E84U1B_metastasis12**	2.47E+08	2.32E+08	8.4	0.010454	0.877456	145934
**K26K-EJL1KW_metastasis11**	2.99E+08	2.81E+08	9.8	0.002934	0.877285	153748
**K26K-EK5C3C_metastasis12**	2.58E+08	2.39E+08	9.4	0.005037	0.876142	145665
**K26K-ELSFBE_metastasis11**	3.24E+08	3.08E+08	8.9	0.012663	0.88393	149153
**K26K-EPTTB9_metastasis11**	3.01E+08	2.01E+08	8	0.011925	0.611784	143025
**K26K-EQ9XTQ_metastasis13**	2.92E+08	2.7E+08	8.7	0.014938	0.880865	146251
**K26K-ES3KU3_tumor11**	2.1E+08	1.9E+08	7.6	0.027005	0.842562	144798
**K26K-ETBARK_tumor11**	1.93E+08	1.77E+08	6.2	0.010109	0.826298	145710
**K26K-EUWQV4_tumor11**	1.02E+09	9.08E+08	7.3	0.007642	0.821068	148502
**K26K-EVQJ85_metastasis21**	1.63E+08	1.51E+08	9.2	0.006399	0.808773	144705
**K26K-EVQJ85_tumor11**	2E+08	1.78E+08	10	0.01269	0.835539	142452
**K26K-EVVHM1_metastasis12**	2.02E+08	1.9E+08	8	0.010352	0.888959	144748
**K26K-F4BWZA_tumor11**	2.91E+08	2.68E+08	9	0.069987	0.871271	143677

**K26K-F5V95D_metastasis12**	2.92E+08	2.71E+08	7.6	0.008281	0.867924	147572
**K26K-FAN1MB_metastasis11**	2.57E+08	2.31E+08	7.2	0.01642	0.819591	145796
**K26K-FCKMTM_metastasis12**	2.21E+08	2.01E+08	6.2	0.010389	0.849315	145738
**K26K-FDGLB6_metastasis12**	1.98E+08	1.8E+08	9.3	0.01061	0.809826	140455
**K26K-FDGLB6_metastasis21**	1.79E+08	1.47E+08	7	0.006215	0.641264	135313
**K26K-FDGLB6_tumor12**	2.4E+08	1.19E+08	8	0.007233	0.448977	139304
**K26K-FDYVED_metastasis12**	2.12E+08	1.98E+08	7.6	0.008335	0.831893	154931
**K26K-FHFEVH_tumor11**	2.91E+08	2.23E+08	3.5	0.00695	0.497316	153818
**K26K-FQZN9K_metastasis11**	2.54E+08	2.29E+08	6.9	0.014738	0.854063	146393
**K26K-FXNF6U_metastasis11**	2.85E+08	2.62E+08	8,1	0.008046	0.865789	151813
**K26K-FZ3PWP_metastasis11**	2.49E+08	2.35E+08	7.5	0.014756	0.86915	151740
**K26K-G1K1C8_metastasis11**	2.33E+08	2.12E+08	8.6	0.008806	0.875084	145393
**K26K-G7Q3UN_tumor11**	2.1E+08	1.93E+08	8.9	0.015724	0.821334	142656
**K26K-G7RGRH_metastasis11**	9.63E+08	8.47E+08	6.4	0.019338	0.803268	154445
**K26K-G8PLW1_metastasis12**	1.53E+08	1.44E+08	9.6	0.004449	0.849292	150013
**K26K-GBZY7E_metastasis12**	2.23E+08	2.02E+08	7.6	0.018489	0.859356	146267
**K26K-GE6LK5_tumor12**	2.6E+08	2.36E+08	8.3	0.004329	0.843276	150452
**K26K-GEBVMR_metastasis12**	2.01E+08	1.79E+08	6.5	0.013623	0.857035	143110
**K26K-GG93LJ_metastasis22**	3.04E+08	2.72E+08	7.4	0.028394	0.845665	142548
**K26K-GGT9WV_metastasis11**	3.2E+08	2.87E+08	8.9	0.009455	0.860038	144348
**K26K-GP1DEZ_metastasis12**	3.52E+08	3.33E+08	9.1	0.016392	0.893603	149773
**K26K-GR7U4J_metastasis11**	2.18E+08	1.95E+08	7.9	0.014272	0.851575	144600
**K26K-GRKG3V_metastasis11**	2.83E+08	2.62E+08	7.4	0.009979	0.860928	147944
**K26K-H9FT8X_metastasis12**	2.35E+08	2.14E+08	7.6	0.009908	0.854701	147654

**K26K-HANJG1_metastasis11**	3.46E+08	3.04E+08	8.6	0.015015	0.715783	142871
**K26K-HGTZ3B_metastasis11**	1.93E+08	1.73E+08	8.8	0.015809	0.857255	143281
**K26K-HGX6HZ_metastasis11**	1.24E+08	1.1E+08	9.1	0.008067	0.815051	143854
**K26K-HGXQVM_metastasis22**	3.35E+08	3.05E+08	9.1	0.0046	0.873193	149107
**K26K-HGXQVM_tumor11**	2.19E+08	2.05E+08	8.9	0.008573	0.901792	143082
**K26K-HH4Q3M_tumor12**	2.37E+08	2.16E+08	9.4	0.014213	0.872879	145987
**K26K-HJDPFX_metastasis11**	2.36E+08	2.15E+08	5.7	0.015689	0.866719	147447
**K26K-HKH6GS_metastasis11**	1.02E+09	8.85E+08	7	0.027519	0.762743	150334
**K26K-HL95W4_metastasis21**	1.38E+08	1.16E+08	8.9	0.03051	0.685683	131482
**K26K-HNE5A5_metastasis11**	3.35E+08	2.82E+08	9	0.001613	0.764553	150630
**K26K-HPGSLN_metastasis11**	2.41E+08	2.22E+08	6.6	0.018604	0.832927	142798
**K26K-HQ88ZT_metastasis11**	2.75E+08	2.5E+08	8.9	0.009718	0.831946	149951
**K26K-HQ88ZT_metastasis21**	1.83E+08	1.63E+08	8.9	0.02371	0.828028	142122
**K26K-HRURBQ_metastasis21**	3.46E+08	3.02E+08	9.4	0.018184	0.788754	144068
**K26K-HTWSX6_metastasis11**	3.25E+08	3E+08	7.7	0.006974	0.864794	148761
**K26K-HV34DF_metastasis12**	2.16E+08	41473702	8.6	0.007767	0.151169	124393
**K26K-J1JGYX_metastasis13**	1.74E+08	1.4E+08	2.7	0.008207	0.715046	145487
**K26K-J1JGYX_metastasis21**	1.52E+08	1.22E+08	1.2	0.012172	0.658272	140606
**K26K-J1JGYX_tumor11**	1.73E+08	1.36E+08	2.3	0.010874	0.667878	141639
**K26K-J1JGYX_tumor21**	1.77E+08	1.39E+08	2.7	0.01035	0.670224	141122
**K26K-J54JQM_metastasis11**	2.57E+08	2.22E+08	6.5	0.016858	0.820651	145404
**K26K-J8FA7A_metastasis11**	4.08E+08	3.73E+08	6.2	0.020916	0.870542	149930
**K26K-JCDSWD_metastasis32**	2.52E+08	2.26E+08	9	0.014054	0.785162	141801
**K26K-JCDSWD_tumor11**	3.11E+08	2.84E+08	7.8	0.011091	0.86141	148934

**K26K-JEQKXE_metastasis31**	2.23E+08	1.91E+08	7.3	0.019431	0.81716	140782
**K26K-JF2LWZ_metastasis11**	3.06E+08	2.86E+08	8.8	0.00923	0.887451	147598
**K26K-JG8K8P_metastasis11**	2.89E+08	2.66E+08	9.7	0.014131	0.86168	148142
**K26K-JHFXVG_metastasis13**	2.39E+08	1.71E+08	2.4	0.021423	0.600278	139896
**K26K-JJERC1_metastasis11**	96573322	84700744	8.6	0.013345	0.794682	134351
**K26K-JJERC1_tumor11**	1.99E+08	1.31E+08	2,9	0.02668	0.6092	141834
**K26K-JPH1C6_metastasis11**	3.17E+08	2.87E+08	8.2	0.005707	0.861352	147522
**K26K-JS49GN_metastasis11**	2.34E+08	2.07E+08	7.8	0.01227	0.772907	140313
**K26K-JS49GN_metastasis21**	3.5E+08	3.16E+08	9.2	0.003127	0.829247	150647
**K26K-KAN58L_metastasis12**	2.81E+08	2.69E+08	9	0.003619	0.887737	149870
**K26K-KBMSKG_tumor12**	2.42E+08	2.17E+08	8.7	0.036134	0.830728	145127
**K26K-KDGJQR_metastasis11**	2.5E+08	2.35E+08	8.4	0.007773	0.888146	147763
**K26K-KGYV17_metastasis11**	1.61E+08	1.45E+08	9.3	0.0689	0.867525	138101
**K26K-KM1V83_metastasis11**	1.81E+08	1.56E+08	9.3	0.017018	0.828013	140859
**K26K-KQYMSV_metastasis11**	2.51E+08	2.2E+08	8.6	0.008093	0.840047	140645
**K26K-KS8MHJ_metastasis11**	3.55E+08	3.22E+08	8.8	0.002804	0.854531	153363
**K26K-KS8MHJ_metastasis21**	1.94E+08	1.74E+08	8.7	0.005934	0.815714	145838
**K26K-KUNL2W_metastasis11**	2.27E+08	2.14E+08	9	0.00668	0.860402	148725
**K26K-KUNL2W_metastasis22**	97624574	87473326	8.2	0.013955	0.813309	139072
**K26K-KV9VSG_tumor11**	3.26E+08	3.03E+08	8.2	0.006052	0.876933	152000
**K26K-KX19MG_tumor12**	2.09E+08	1.55E+08	6.1	0.010398	0.382581	135187
**K26K-KYLKAW_metastasis12**	3.11E+08	2.47E+08	8.8	0.005772	0.710995	143401
**K26K-KYLKAW_tumor11**	2.27E+08	2.07E+08	8.1	0.00335	0.837256	139791
**K26K-L31YS4_metastasis11**	1.18E+08	1.1E+08	8.8	0.01364	0.895258	136842

**K26K-L3MWGQ_metastasis11**	2.06E+08	1.25E+08	2.9	0.009571	0.490303	150410
**K26K-L5XH9B_metastasis11**	1.8E+08	1.69E+08	9.6	0.003908	0.851522	147513
**K26K-L5Z7JE_tumor11**	2.97E+08	2.75E+08	7.8	0.008079	0.864625	150575
**K26K-LA3ZXV_tumor13**	1.85E+08	1.55E+08	2.5	0.012582	0.726534	150715
**K26K-LEVSEC_metastasis11**	2.46E+08	2.27E+08	8.1	0.015223	0.861691	146367
**K26K-LG21QX_metastasis11**	3.63E+08	2.47E+08	8.5	0.007135	0.635244	140355
**K26K-LHW5P8_metastasis12**	2.74E+08	2.37E+08	9.4	0.013762	0.818152	148537
**K26K-LKHUJ5_metastasis11**	2.27E+08	2E+08	9	0.006751	0.759288	145824
**K26K-LKHUJ5_tumor11**	3.03E+08	2.82E+08	7.4	0.011596	0.864375	148355
**K26K-LKKMR2_metastasis11**	1.95E+08	1.78E+08	9.5	0.007226	0.86751	144655
**K26K-LKP6WZ_metastasis12**	2.16E+08	1.97E+08	8.4	0.010521	0.859949	145592
**K26K-LPBX5B_metastasis12**	3.08E+08	2.8E+08	8.4	0.009489	0.862169	145544
**K26K-LUMBC5_metastasis11**	2.32E+08	2.07E+08	7.7	0.018338	0.841692	145832
**K26K-LW7WC8_metastasis13**	4.58E+08	4.33E+08	8.1	0.008754	0.891076	156626
**K26K-LXFJ6Q_tumor12**	2.27E+08	2.1E+08	8.7	0.008699	0.864905	150663
**K26K-M61EFR_metastasis11**	2.38E+08	2.2E+08	8.3	0.003616	0.862673	145595
**K26K-M61EFR_metastasis22**	2.65E+08	2.45E+08	8.5	0.010167	0.860371	145547
**K26K-MAEFZL_metastasis11**	3.77E+08	3.48E+08	8	0.01076	0.88796	147018
**K26K-MF6NDW_tumor12**	2.34E+08	2.2E+08	8.4	0.003843	0.860589	154066
**K26K-MGTYZH_metastasis11**	2.01E+08	1.83E+08	8.5	0.009646	0.851895	147671
**K26K-MLPAW4_tumor11**	1.1E+08	93850824	1.8	0.010225	0.709421	144216
**K26K-MMXGSX_metastasis13**	1.72E+08	1.38E+08	3.6	0.016556	0.677265	149195
**K26K-MMXGSX_metastasis32**	1.13E+08	1E+08	9	0.00716	0.747495	136751

**K26K-MNVK2W_metastasis12**	2.7E+08	2.52E+08	8.7	0.011112	0.885586	147406
**K26K-MXSQTG_tumor11**	1.81E+08	1.68E+08	6.2	0.012744	0.880026	143604
**K26K-MYECCH_metastasis11**	3.26E+08	2.99E+08	8,1	0.012252	0.869928	146044
**K26K-N2D9XV_metastasis11**	2.3E+08	2.15E+08	8	0.003394	0.869744	150546
**K26K-N3VNSA_metastasis11**	2.28E+08	2.01E+08	5.6	0.020942	0.8434	135145
**K26K-N422KN_metastasis11**	3.05E+08	2.88E+08	9.5	0.005946	0.870383	151718
**K26K-N9SB5N_tumor11**	3.16E+08	2.93E+08	9.1	0.005593	0.878772	150506
**K26K-N9SB5N_tumor21**	3.38E+08	3.04E+08	7.5	0.006693	0.833754	153785
**K26K-NBST9B_metastasis22**	3.54E+08	3.27E+08	8.6	0.001683	0.870044	151521
**K26K-NDDF92_metastasis11**	2.03E+08	1.89E+08	8.2	0.009094	0.883037	142233
**K26K-NDDF92_metastasis21**	2.42E+08	1.95E+08	6.9	0.011533	0.632829	144585
**K26K-NDDF92_tumor11**	1.74E+08	1.63E+08	9.7	0.004182	0.861792	149327
**K26K-NEG3MQ_metastasis12**	2.73E+08	2.52E+08	7.9	0.041145	0.814549	149924
**K26K-NKAGPR_metastasis11**	2.57E+08	2.35E+08	8.7	0.014634	0.876615	148053
**K26K-NNVS4U_tumor12**	1.97E+08	1.88E+08	9.4	0.008258	0.905744	146853
**K26K-NPC5CB_metastasis11**	3.1E+08	2.66E+08	8.3	0.003537	0.796125	145263
**K26K-NQH6T5_metastasis21**	2.8E+08	2.6E+08	7.9	0.012587	0.884769	147512
**K26K-NUUDDJ_tumor32**	2.1E+08	1.99E+08	7.3	0.007048	0.897582	149694
**K26K-NV29AR_metastasis12**	3.69E+08	3.39E+08	9.5	0.004309	0.852564	151564
**K26K-NYMP4W_metastasis12**	2.19E+08	2.04E+08	9	0.00776	0.881169	146470
**K26K-P6W7FU_metastasis11**	2.22E+08	2.1E+08	9.2	0.004006	0.887429	150112
**K26K-P76N2B_metastasis12**	2.87E+08	2.73E+08	8.6	0.010488	0.876055	150894
**K26K-P7V4JK_metastasis11**	1.06E+08	91023124	6.7	0.013156	0.757705	129742

**K26K-P7V4JK_tumor11**	2.22E+08	2.03E+08	8.5	0.006662	0.863024	145350
**K26K-P7V4JK_tumor21**	2.07E+08	1.88E+08	7.1	0.008868	0.829243	148652
**K26K-P8EEDV_tumor11**	1.04E+09	8.64E+08	8.1	0.080028	0.693867	162529
**K26K-PAZNZP_metastasis11**	2.39E+08	2.27E+08	9.7	0.004527	0.87356	149072
**K26K-PCA7BN_metastasis12**	2.31E+08	2.18E+08	8.2	0.013422	0.894951	147271
**K26K-PDJUSD_metastasis11**	3.07E+08	2.8E+08	7.6	0.009729	0.879793	142990
**K26K-PGV6SL_metastasis11**	2.13E+08	1.86E+08	7.4	0.047711	0.831533	141221
**K26K-PJV57M_metastasis11**	2.3E+08	75082984	7.6	0.004523	0.301794	120505
**K26K-PN66E8_metastasis21**	2.75E+08	2.55E+08	9.3	0.005607	0.849696	152048
**K26K-PPGH4E_metastasis12**	1.06E+09	9.09E+08	9	0.014752	0.751805	159496
**K26K-PT3E2X_metastasis11**	2.06E+08	1.89E+08	6.9	0.006801	0.870176	145223
**K26K-PYFRR7_metastasis11**	2.61E+08	2.39E+08	6.7	0.00943	0.873813	147877
**K26K-Q25AYS_metastasis11**	2.7E+08	2.55E+08	9.2	0.003166	0.831096	150187
**K26K-Q4JZ17_metastasis21**	1.86E+08	1.68E+08	7	0.01551	0.863539	143248
**K26K-Q6TE19_metastasis12**	1.62E+08	1.43E+08	8.3	0.016319	0.821292	140071
**K26K-Q99D4V_metastasis14**	2.14E+08	2.01E+08	9.2	0.007634	0.858594	149892
**K26K-QA7GAW_metastasis11**	1.05E+09	9.4E+08	7.1	0.009146	0.817131	143098
**K26K-QBQ2UL_tumor11**	2.79E+08	2.41E+08	7	0.01434	0.828582	145548
**K26K-QL2LML_metastasis21**	1.9E+08	1.72E+08	7.3	0.016003	0.856619	145439
**K26K-QRC8PF_metastasis12**	1.71E+08	1.59E+08	8.2	0.001626	0.843699	144857
**K26K-QRC8PF_tumor31**	1.73E+08	1.34E+08	4.1	0.018545	0.584204	150665
**K26K-QSDNXA_tumor11**	1.64E+08	1.52E+08	8.5	0.013969	0.889022	140144
**K26K-QXX4LD_metastasis12**	3.11E+08	2.92E+08	8.8	0.00807	0.900546	149856
**K26K-R3SK9S_metastasis11**	3.24E+08	2.63E+08	9.1	0.010209	0.767372	139200

**K26K-R4ATRT_tumor12**	2.82E+08	2.55E+08	6.8	0.007358	0.864878	143186
**K26K-R5DVUL_metastasis11**	2.35E+08	2.15E+08	7.2	0.010897	0.846311	149711
**K26K-R8A4NH_metastasis11**	2.54E+08	97367962	6.4	0.009882	0.308333	141242
**K26K-R8KRU9_metastasis11**	2.44E+08	2.32E+08	8.9	0.002753	0.882543	150383
**K26K-RA678B_metastasis11**	2.15E+08	1.96E+08	7.7	0.014079	0.873621	143329
**K26K-RCX5HZ_metastasis11**	3.88E+08	3.44E+08	6.5	0.015939	0.825914	149005
**K26K-RDH1KP_metastasis12**	2.18E+08	2.03E+08	9.1	0.012886	0.880434	145314
**K26K-RJEPF5_metastasis11**	2.58E+08	2.46E+08	8.7	0.012781	0.888162	150724
**K26K-RPNFY9_metastasis11**	4.17E+08	3.79E+08	9.4	0.003368	0.836757	151733
**K26K-RSNUH1_metastasis12**	2.38E+08	1.17E+08	6	0.012139	0.430218	140817
**K26K-RSYYVH_metastasis11**	4.22E+08	3.94E+08	9.2	0.016989	0.866846	155548
**K26K-RWBB37_metastasis11**	2.36E+08	2.22E+08	8.9	0.00948	0.888799	147280
**K26K-RX8HVN_metastasis12**	2.25E+08	1.91E+08	6.1	0.012389	0.64344	147560
**K26K-S2A6CJ_metastasis12**	3.13E+08	2.9E+08	6.8	0.020386	0.879005	152259
**K26K-S3CZ8N_tumor11**	3.24E+08	3E+08	8.9	0.012666	0.871573	154424
**K26K-S3FTVP_metastasis11**	89681600	72017478	8.1	0.01494	0.662179	123100
**K26K-SAUCL3_metastasis11**	3.49E+08	3.19E+08	10	0.007562	0.828399	149454
**K26K-SD4V5Q_tumor12**	1.64E+08	1.54E+08	7.6	0.0223	0.872654	146847
**K26K-SKGL7S_metastasis13**	1.47E+08	1.19E+08	2.5	0.022809	0.711949	147819
**K26K-SMQDUN_metastasis11**	1.94E+08	1.8E+08	9.6	0.004648	0.868126	147383
**K26K-SNG1R3_metastasis11**	3.19E+08	2.93E+08	7.3	0.01284	0.868869	149211
**K26K-SNVUBF_metastasis21**	3.37E+08	3.05E+08	7.7	0.018194	0.852107	152216
**K26K-SSBAF2_metastasis11**	9.54E+08	8.38E+08	7.4	0.013684	0.797377	153538
**K26K-SU166F_metastasis11**	2.48E+08	2.34E+08	8.4	0.010987	0.876464	151806

**K26K-SU24BN_metastasis11**	4.07E+08	3.7E+08	8.6	0.006678	0.862162	147807
**K26K-SU24BN_tumor12**	2.1E+08	1.91E+08	8.1	0.005533	0.811775	147649
**K26K-SV7281_metastasis11**	2.01E+08	1.88E+08	5,8	0.005796	0.864185	148467
**K26K-SWYTUR_metastasis11**	3.25E+08	2.9E+08	6.4	0.019397	0.803227	149230
**K26K-SXFM78_metastasis12**	1.73E+08	1.62E+08	7.9	0.015973	0.878256	145079
**K26K-T7EFJK_metastasis11**	3.08E+08	2.35E+08	6.5	0.01848	0.64864	137452
**K26K-T7EFJK_metastasis21**	3.75E+08	3.47E+08	8.7	0.035501	0.835059	152830
**K26K-TABQ88_metastasis11**	2.11E+08	1.87E+08	6.5	0.012686	0.856196	137649
**K26K-TBG1FN_metastasis11**	2.68E+08	2.51E+08	9	0.014489	0.883857	148517
**K26K-TG5T5N_metastasis11**	2.73E+08	2.46E+08	8.4	0.016757	0.866034	144206
**K26K-TL7317_metastasis11**	4.01E+08	3.67E+08	8,5	0.015077	0.879386	145384
**K26K-TMX9UN_metastasis12**	2E+08	1.85E+08	8.8	0.007927	0.861539	144207
**K26K-TMX9UN_tumor11**	2.13E+08	1.93E+08	6.8	0.014249	0.860598	142478
**K26K-TTTSQQ_metastasis11**	1.97E+08	1.81E+08	9.1	0.008491	0.88655	142617
**K26K-U13K48_metastasis11**	2.47E+08	2.34E+08	9	0.001969	0.868654	153598
**K26K-U1CUNE_metastasis11**	2.1E+08	1.18E+08	6,3	0.009354	0.540551	133831
**K26K-U3ERTU_metastasis11**	2.84E+08	2.71E+08	8.8	0.007727	0.869732	151459
**K26K-U48257_tumor11**	2.77E+08	2.66E+08	9.7	0.004159	0.891469	147592
**K26K-U9L51W_metastasis13**	1.9E+08	1.32E+08	1.9	0.013951	0.535121	148532
**K26K-UGZH2U_metastasis11**	3.09E+08	2.91E+08	8.5	0.004146	0.852272	155140
**K26K-ULPXJ3_metastasis21**	1.26E+08	1.13E+08	6.1	0.018794	0.81487	132462
**K26K-UVAXJ4_metastasis11**	2.52E+08	2.35E+08	8.7	0.006068	0.854347	147939
**K26K-UWDBJ5_tumor12**	2.98E+08	2.77E+08	8.4	0.023373	0.886847	146082
**K26K-UWRHM6_metastasis11**	2.72E+08	2.24E+08	9.5	0.003753	0.79942	137025

**K26K-UY56QP_metastasis11**	2.03E+08	1.86E+08	8.1	0.014577	0.882519	141525
**K26K-UYPA78_metastasis12**	1.87E+08	1.62E+08	7.5	0.01553	0.760312	138956
**K26K-UYPA78_tumor11**	4.3E+08	3.87E+08	8,6	0.010073	0.834749	154434
**K26K-UZ6G9R_tumor11**	3.12E+08	2.68E+08	5.3	0.009319	0.663972	150079
**K26K-V2PF6M_metastasis12**	2.68E+08	2.52E+08	8.7	0.007865	0.865901	149065
**K26K-V3TLD6_metastasis11**	1.78E+08	1.53E+08	7.5	0.014384	0.826827	138277
**K26K-VAHD5J_metastasis12**	2.44E+08	2.26E+08	8.5	0.030511	0.880246	147605
**K26K-VBGZC7_metastasis11**	3.01E+08	2.84E+08	8.1	0.005327	0.847065	151292
**K26K-VBGZC7_metastasis21**	2.73E+08	2.59E+08	8.7	0.004872	0.872727	149371
**K26K-VBGZC7_metastasis32**	2.51E+08	2.02E+08	7.2	0.022685	0.602761	137572
**K26K-VDQXWG_tumor11**	1.96E+08	1.79E+08	7.2	0.009115	0.849137	147822
**K26K-VDWGQA_metastasis12**	3.55E+08	3.13E+08	8.3	0.008917	0.806828	153018
**K26K-VJ58WY_metastasis41**	2.02E+08	1.7E+08	7.8	0.008743	0.511218	139973
**K26K-VR4F9W_metastasis13**	3.09E+08	2.18E+08	3	0.010727	0.414751	155636
**K26K-VSQ56S_tumor11**	3.31E+08	3E+08	6.2	0.007423	0.851061	149084
**K26K-VWASDF_metastasis21**	3.01E+08	1.91E+08	3.1	0.019074	0.372114	151006
**K26K-VWASDF_tumor12**	3.48E+08	3.09E+08	8.5	0.005797	0.816658	151192
**K26K-VWASDF_tumor21**	1.08E+08	95927690	9	0.006113	0.814245	138630
**K26K-WAMNC8_metastasis12**	2.22E+08	2.04E+08	8.6	0.015993	0.874816	146037
**K26K-WDFWZM_metastasis12**	2.76E+08	2.54E+08	8.3	0.009791	0.857382	149777
**K26K-WHAFAB_metastasis11**	2.3E+08	2.15E+08	9.4	0.003999	0.872621	148135
**K26K-WHAFAB_metastasis21**	3.99E+08	3.5E+08	8.6	0.006398	0.830714	145548
**K26K-WHAFAB_metastasis31**	1.32E+08	1.05E+08	9.8	0.006342	0.688722	138635

**K26K-WLLBUL_metastasis12**	2.74E+08	2.57E+08	9.3	0.007976	0.869179	146063
**K26K-WPZ6MB_metastasis11**	3.29E+08	3.04E+08	9.7	0.004336	0.856319	155004
**K26K-WQ6ZNA_metastasis11**	1.96E+08	1.86E+08	8.8	0.007955	0.891971	147094
**K26K-WS87L3_metastasis11**	2.61E+08	2.15E+08	8.6	0.012549	0.784883	140858
**K26K-WSBAGM_tumor22**	3.33E+08	2.86E+08	8.9	0.011491	0.826621	139761
**K26K-XBFHQ2_metastasis21**	2.32E+08	1.89E+08	8.7	0.006674	0.59306	131560
**K26K-XBFN4M_tumor11**	2.53E+08	2.42E+08	9.2	0.00451	0.887397	152703
**K26K-XBX9RY_metastasis11**	2.78E+08	2.61E+08	7.9	0.013507	0.88982	144581
**K26K-XEKNVJ_metastasis11**	2.72E+08	2.5E+08	6.4	0.038263	0.867753	148272
**K26K-XEU9W5_metastasis11**	2.32E+08	2.18E+08	9.1	0.004449	0.865177	150011
**K26K-XF7MPU_metastasis11**	2.45E+08	2.22E+08	7.7	0.012079	0.864826	145864
**K26K-XU7ND6_metastasis11**	2E+08	1.53E+08	8.4	0.010442	0.730331	138806
**K26K-XVLCD2_tumor11**	1.74E+08	1.65E+08	8.3	0.015624	0.898593	142046
**K26K-XXHLCZ_metastasis11**	3.05E+08	2.67E+08	7.4	0.012463	0.784196	151751
**K26K-Y1N726_metastasis11**	1.91E+08	1.78E+08	7.2	0.006272	0.848773	143535
**K26K-Y2QMVE_metastasis11**	3.13E+08	2.92E+08	9.4	0.017966	0.848861	149463
**K26K-Y2QMVE_metastasis22**	2.31E+08	2.05E+08	8.7	0.021182	0.802352	146940
**K26K-Y41ABR_metastasis11**	3.13E+08	2.86E+08	8.2	0.007542	0.84051	154003
**K26K-Y665TN_tumor11**	2.54E+08	2.37E+08	7.3	0.022386	0.887343	144717
**K26K-Y74RYK_metastasis11**	2.76E+08	2.57E+08	8.7	0.006182	0.890317	147735
**K26K-Y7EELU_metastasis12**	2.79E+08	2.58E+08	8.2	0.012822	0.865824	147866
**K26K-Y8ATLQ_metastasis12**	2.78E+08	2.61E+08	8.9	0.011796	0.892197	149891
**K26K-Y9BDCK_metastasis11**	2.66E+08	2.42E+08	9.4	0.005398	0.869846	145288
**K26K-Y9BDCK_metastasis21**	2.15E+08	1.98E+08	6	0.011383	0.816672	143919

**K26K-YA19QK_metastasis11**	3.34E+08	3.06E+08	7.8	0.023727	0.863883	150144
**K26K-YA19QK_metastasis21**	3.02E+08	2.69E+08	6.6	0.049046	0.813459	147202
**K26K-YA19QK_metastasis41**	1.75E+08	1.55E+08	6	0.147492	0.797192	142192
**K26K-YB2V81_metastasis11**	2.81E+08	2.59E+08	9.1	0.002479	0.862271	149995
**K26K-YF7GYQ_metastasis13**	1.84E+08	1.38E+08	4.3	0.013006	0.605411	150824
**K26K-YF7GYQ_tumor11**	2.07E+08	1.92E+08	7.7	0.046406	0.857652	145989
**K26K-YF9FHJ_metastasis11**	2.54E+08	2.25E+08	7.9	0.021894	0.835013	146794
**K26K-YFJGXZ_tumor11**	3.62E+08	3.36E+08	7.4	0.011789	0.867842	150549
**K26K-YJ9GCJ_metastasis11**	2.66E+08	2.43E+08	8.6	0.007904	0.852161	149659
**K26K-YJ9GCJ_tumor12**	1.74E+08	1.59E+08	7.2	0.006787	0.821347	143711
**K26K-YJB543_metastasis12**	3.02E+08	2.78E+08	7.2	0.01245	0.873901	145908
**K26K-YRWDKX_metastasis11**	1.8E+08	1.19E+08	3.8	0.036632	0.421534	144476
**K26K-YRWDKX_tumor11**	2.86E+08	2.47E+08	9.6	0.038282	0.836379	142556
**K26K-YU91XW_metastasis11**	2.39E+08	1.74E+08	8.5	0.018435	0.688691	139640
**K26K-YXC7H4_tumor12**	2.61E+08	2.45E+08	5.7	0.019516	0.863044	149650
**K26K-YYMX7V_metastasis11**	2.14E+08	2.03E+08	9.7	0.006641	0.881604	143186
**K26K-YYMX7V_metastasis22**	2.56E+08	2.3E+08	8.8	0.009469	0.864546	145826
**K26K-YYP1DV_metastasis11**	2.57E+08	2.41E+08	8.6	0.031445	0.877767	147122
**K26K-Z3LWUS_metastasis11**	2.03E+08	1.88E+08	9.6	0.007641	0.855405	147680
**K26K-Z559NG_metastasis12**	1.03E+09	9.17E+08	8	0.008767	0.817851	151038
**K26K-Z6VH73_metastasis11**	2.39E+08	2.22E+08	7.6	0.006795	0.854075	152772
**K26K-Z8XJY9_metastasis11**	2.87E+08	2.59E+08	8.8	0.014441	0.829757	149219
**K26K-Z8Z4HX_metastasis11**	6.6E+08	5E+08	8,5	0.016359	0.700909	150617

**K26K-ZDRXB2_tumor11**	2.62E+08	2.47E+08	6.8	0.004501	0.787769	154039
**K26K-ZDUSLY_metastasis12**	2.61E+08	2.29E+08	6.9	0.0149	0.737675	146476
**K26K-ZH5GAP_tumor11**	1.55E+08	1.4E+08	8.5	0.017582	0.842238	144593
**K26K-ZUWXZY_metastasis22**	2.63E+08	2.41E+08	7.9	0.004852	0.833491	148397
**K26K-ZWJDB4_metastasis41**	1.08E+08	96560686	9.7	0.008767	0.800994	134382
**K26K-ZZHJUA_metastasis11**	2.71E+08	2.49E+08	7.4	0.007857	0.851213	150515
